# Does metabolism constrain bird and mammal ranges and predict shifts in response to climate change?

**DOI:** 10.1002/ece3.4537

**Published:** 2018-12-10

**Authors:** Lauren B. Buckley, Imran Khaliq, David L. Swanson, Christian Hof

**Affiliations:** ^1^ Department of Biology University of Washington Seattle Washington; ^2^ Zoology Department Ghazi University Punjab Pakistan; ^3^ Department of Biology University of South Dakota Vermillion South Dakota; ^4^ Terrestrial Ecology Research Group Department of Ecology and Ecosystem Management School of Life Sciences Weihenstephan Technical University of Munich Freising Germany

**Keywords:** distribution, endotherm, metabolic expansibility, metabolic scope, range limit, thermal neutral zone

## Abstract

Mechanistic approaches for predicting the ranges of endotherms are needed to forecast their responses to environmental change. We test whether physiological constraints on maximum metabolic rate and the factor by which endotherms can elevate their metabolism (metabolic expansibility) influence cold range limits for mammal and bird species. We examine metabolic expansibility at the cold range boundary (ME_CRB_) and whether species’ traits can predict variability in ME_CRB_ and then use ME_CRB_ as an initial approach to project range shifts for 210 mammal and 61 bird species. We find evidence for metabolic constraints: the distributions of metabolic expansibility at the cold range boundary peak at similar values for birds (2.7) and mammals (3.2). The right skewed distributions suggest some species have adapted to elevate or evade metabolic constraints. Mammals exhibit greater skew than birds, consistent with their diverse thermoregulatory adaptations and behaviors. Mammal and bird species that are small and occupy low trophic levels exhibit high levels of ME_CRB_. Mammals with high ME_CRB_ tend to hibernate or use torpor. Predicted metabolic rates at the cold range boundaries represent large energetic expenditures (>50% of maximum metabolic rates). We project species to shift their cold range boundaries poleward by an average of 3.9° latitude by 2070 if metabolic constraints remain constant. Our analysis suggests that metabolic constraints provide a viable mechanism for initial projections of the cold range boundaries for endotherms. However, errors and approximations in estimating metabolic constraints (e.g., acclimation responses) and evasion of these constraints (e.g., torpor/hibernation, microclimate selection) highlight the need for more detailed, taxa‐specific mechanistic models. Even coarse considerations of metabolism will likely lead to improved predictions over exclusively considering thermal tolerance for endotherms.

## 1 INTRODUCTION

Environmental temperatures govern the performance and energy use, and ultimately the abundance and distribution, of animals (Bozinovic, Calosi, & Spicer, 2011). Performance and energetic constraints provide a powerful basis for projecting responses to climate change because the constraints should extrapolate better into novel environments than statistical correlations (Radeloff et al., 2015). Models using heat budgets to translate environmental conditions into the body temperatures of ectotherms and quantifying limitations on performance and activity durations can robustly predict patterns of abundance and distribution (Buckley et al., 2010; Kearney & Porter, 2009). The translation is more complex for endothermic animals because they can use endogenous heat production to maintain their body temperatures under a wide range of environmental thermal conditions if available resources and physiological capacities are sufficient (Boyles, Seebacher, Smit, & McKechnie, 2011; Buckley, Hurlbert, & Jetz, 2012; McNab, 2012). Thus, few mechanistic approaches predict endotherm distributions (but see examples reviewed in Boyles et al., 2011). Many attempts to predict endotherm distributions are based on air temperature without considering capacity for endogenous heat production (Fuller, Mitchell, Maloney, & Hetem, 2016). Mitchell et al. (2018) review misconceptions of thermal physiology that plague predictive models of mammalian responses to climate change. Several recent examples employ biophysical models to estimate metabolic constraints, activity limitations, and water balance for focal endotherms (Kearney, Porter, & Murphy, 2016; Mathewson et al., 2017), but can these approaches be generalized?

Fundamental physiological constraints on metabolic systems, including to the mobilization, transport and use of oxygen and substrates, limit maximum metabolic rate and the factor by which endotherms can elevate their metabolism (Humphries, Umbanhowar, & McCann, 2004; Stager et al., 2015). An initial test of metabolic constraints (Root, 1988) suggested that the cold range boundaries of passerine birds in North America coincided with winter metabolic rates at the cold range boundary being elevated by a factor of 2.5 over basal metabolic rates (BMR), but subsequent analyses (Canterbury, 2002; Repasky, 1991) have questioned the generality of metabolic constraints due to the limited biological, distributional, and environmental data available or poor fit between range boundaries and temperature isotherms.

Physiological measurements indicate metabolic constraints and adaptations. Maximum cold‐induced metabolic rate (summit metabolism, M_sum_) is greater in cold environments (Wiersma, Muñoz‐Garcia, Walker, & Williams, 2007) and is phylogenetically conserved (Stager et al., 2015; Swanson & Garland, 2009). Observations that metabolic scope, the extent to which M_sum_ is elevated over BMR, increases poleward are explained by two related hypotheses: The Climate Variability Hypothesis (Ghalambor, Huey, Martin, Tewksbury, & Wang, 2006; Janzen, 1967; Stevens, 1989) proposes that variable climates at high latitudes and altitudes select for greater flexibility in metabolic rate. The Cold Adaptation Hypothesis (Swanson & Garland, 2009) proposes that extreme winter temperatures in cold climates select for high M_sum_. Extensions of classic work on adaptations to regulate heat (Scholander, 1955; Scholander, Hock, Walters, Johnson, & Irving, 1950) find that adaptation to environmental conditions, including adjustments to insulation, alters both basal metabolic rate (BMR) and heat conductance in birds and mammals (Fristoe et al., 2015). Birds and mammals with more poleward range limits that experience colder minimum temperatures can tolerate colder temperatures without elevating metabolism (Khaliq et al., 2015).

Here we leverage extensive metabolic, distribution, and phylogenetic datasets (Fristoe et al., 2015; Khaliq, Hof, Prinzinger, Böhning‐Gaese, & Pfenninger, 2014) to test the viability of using metabolic constraints to project bird and mammal distributions. Specifically, we estimate the factor by which metabolism is elevated at the cold range boundaries (metabolic expansibility, ME_CRB_). We expect the distribution of ME_CRB _to be normal and strongly peaked if the cold range edges of birds and mammals are limited by the capacity of their metabolic systems to maintain approximate temperature homeostasis. A peaked distribution would indicate similar limits to ME_CRB _across birds and mammals that differ substantially in geographic distribution, habitat, traits, and life history. However, skew in the distribution could reflect either species that are metabolically adapted to or able to evade cold conditions (positive skew) or species that are particularly sensitive to cold (negative skew). Species may evade extreme temperatures by adjusting activity times (e.g., diurnality) or the maintenance of body temperatures (e.g., use of hibernation or torpor) or by selecting favorable microclimates or using behavioral thermoregulation (e.g., communal roosting). Because mammals use strategies to evade full exposure to winter cold (e.g., hibernation, use of subnivean space) to a much greater degree than birds (Ruf & Geiser, 2015; Swanson, 2010; Williams, Henry, & Sinclair, 2014), we expect that mammals will exhibit more cases with high ME_CRB_ values and estimated range boundary metabolic rates approaching or exceeding M_sum_ than birds.

We test whether physiological, behavioral, and ecological traits (body size, nocturnality, torpor use, diet) associated with adaptation or evasion correspond to higher ME_CRB_ values. Evasion would result in high ME_CRB_ values due to CRB temperatures being colder than those actually experienced by the animals, resulting in overestimation of heating requirements. Body size influences the ability to use potential microclimates as well as metabolic rates and thermal inertia (Mitchell et al., 2018). Trophic levels influence the seasonal availability of food and metabolic rate (McNab, 2008, 2009 ). We also examine the conservatism of traits and metabolic expansibility at the cold range boundary (ME_CRB_) across the phylogeny. Finally, evidence for metabolic constraints suggests that (in the absence of adaptation or acclimation) species will follow thermal isoclines through climate change. We thus use ME_CRB_ as an initial approach to project ranges and range shifts in response to predicted climate change.

## 2 MATERIALS AND METHODS

We used the Scholander‐Irving model of homeothermic endothermy to estimate the factor by which metabolism is elevated at the cold range boundaries (as in Root, 1988). We recognize that most species deviate from the idealized model, particularly due to widespread and frequent heterothermy and phenotypic plasticity (Fuller et al., 2016). However, we hold that the model is the most tractable and general approach to test for metabolic constraints among numerous taxonomically and physiologically diverse species. Further, we test for deviations from the idealized model due to factors such as heterothermy as discussed below. We feel that (in disagreement with some reviewers) the Scholander‐Irving model and the best tractably available data for parameterization are adequate as an initial step toward assessing the occurrence of metabolic constraints. Violations of assumptions of the Scholander‐Irving model should obscure evidence for metabolic constraints, making our test conservative.

**Figure 1:**
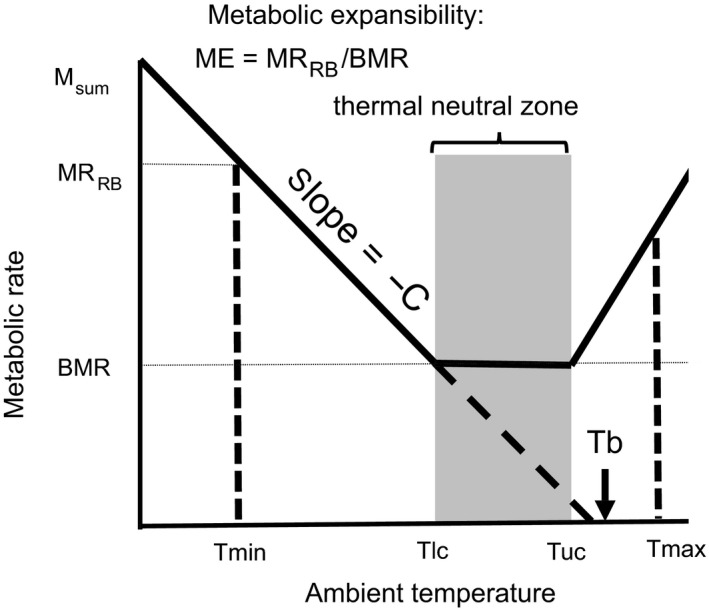
How ambient temperature governs metabolic rate. The thermal neutral zone [bounded by lower (*T*_lc_) and upper (*T*_uc_) critical temperatures] is the range of temperatures over which endotherms are able to maintain their basal metabolic rate (BMR). We use the minimum (*T*_min_) and maximum (*T*_max_) ambient temperatures across a species’ range to estimate sustained metabolic rate at the range boundary (MR_CRB_). We calculate metabolic expansibility (ME_CRB_) as MR_CRB_/BMR and depict maximum (summit) metabolic capacity (M_sum_). Thermal conductance (*C*) is calculated as the slope of the line terminating at body temperature (*T*_b_)

We estimated resting metabolic rate (ml O_2_ h^−1^) at the cold range boundary as MR_CRB_ = (*T*_lc_ − *T*_min_)*C* + BMR, where *T*_lc_ is the lower critical temperatures bounding the lower limit of the thermal neutral zone (TNZ); *T*_min_ is the coldest winter environmental temperatures at the cold range boundary; BMR is basal metabolic rate (ml O_2_ h^−1^), and *C* is thermal conductance (ml O_2_ h^−1^ °C^−1^) (Figure 1, see Section 2.1 for details on parameterization). We calculated metabolic expansibility at the cold range boundary as ME_CRB_ = MRCRB/BMR. An alternative to cold environments selecting for increased ME_CRB_ is selection for increased metabolic scope and M_sum_ (M_sum_ = BMR + metabolic scope). We thus also examine MR_CRB_/M_sum_ as a cold boundary constraint. We conducted a coarse analysis of warm range boundaries following analogous methods and reported in the discussion, but we focus on cold range boundaries because they are more likely governed by metabolic constraints than are warm range boundaries. At warm range boundaries, the capacity for evaporative cooling may be more limiting than the associated metabolic costs and minimal endogenous heating is favored (McKechnie, Whitfield, et al., 2016; Tieleman & Williams, 2000). Our estimates of ME_CRB_ are approximate (see Discussion) in that they do not account for additional factors such as use of solar radiation, convective heat loss, microclimate variation, microhabitat selection, and behavioral thermoregulation (Mitchell et al., 2018; Porter & Kearney, 2009).

### 2.1 Data

We restricted our analysis to resident (nonmigratory) species. We omitted species on islands and those with latitudinal range limits constrained by continental boundaries. We additionally restricted our analysis to cold range boundaries with temperatures below the *T*_lc_ (omitted 1% of species). Accounting for these constraints and limitations on available physiological data, we analyzed 210 and 61 cold range boundaries for mammal and bird species, respectively. The species are broadly distributed across latitudes: the mean and median of the absolute latitude of the poleward range limits are 39.7° and 35.2° degrees, respectively.

For species distribution data, we used the IUCN range maps for mammals (Patterson et al., 2005) and the BirdLife range maps for birds (BirdLife International & NatureServe, 2014). We calculated temperatures at the range boundaries (*T*_min_ and *T*_max_) using BIO5 (max daily temperature of warmest month) and BIO6 (min daily temperature of coldest month) at five‐minute resolution from the WorldClim dataset (Hijmans, Cameron, Parra, Jones, & Jarvis, 2005). Data are interpolated from air temperature at weather stations (generally 2 m high). Trait data are insufficient to account for microhabitat use (e.g., burrows or under snow), but our trait analysis does provide some indication of exposure to air temperatures. We extracted the grid cells at the northern and southern extremes of the species’ distribution for each 5‐minute longitudinal band. We quantified the degree to which range boundaries follow temperature isoclines as the standard deviation and median absolute deviation (mad, R function mad) of cells along the range boundaries. Subsequently, we estimated *T*_min_ and *T*_max_ as the median of the grid cells along the cold and warm range boundaries, respectively. We checked that minimum and maximum temperatures were sufficiently constant across the range boundaries for our results to be robust to our selection of the median (Figure S1). Current data are normals for 1950–2000, and future data are downscaled global climate model (GCM) projections from CMIP5 (IPCC Fifth Assessment) averaged over 2061–2080. We examined output from both the HadGEM2‐AO and CCSM4 models assuming a midrange greenhouse gas concentration scenario (Representative Concentration Pathway RCP6.0, indicates a 6 W/m^2^ increase in radiative forcing in 2,100 relative to pre‐industrial values, https://cmip-pcmdi.llnl.gov/cmip5/). Data were accessed using the getData function in the R package raster.

The bounds of the TNZ (*T*_lc_ and *T*_uc_, °C) and set point body temperature (*T*_b_, °C), were compiled from the literature by Khaliq et al. (2014) and Fristoe et al. (2015). We incorporated data compiled for additional species (Bozinovic, Ferri‐Yáñez, Naya, Araújo, & Naya, 2014; Canterbury, 2002; Riek & Geiser, 2013). We used BMR data from Fristoe et al. (2015) and McNab (2008, 2009 ) after assessing whether the data met criteria for data quality (see below). We extracted M_sum_ data for 20 mammal and six bird species from existing compilations (Lovegrove, 2005; Rezende, Bozinovic, & Garland, 2004; Stager et al., 2015; Swanson & Garland, 2009). Those MR values reported in watts were converted to oxygen consumption assuming a factor of 179 ml O_2_ h^−1^ W^−1^, which corresponds to lipid metabolism (Schmidt‐Nielsen, 1997). Minimum conductance was estimated as the absolute value of the slope of the line connecting *T*_lc_ at BMR to *T*_b_ when metabolic rate is 0: *C*_min_ = |(0‐BMR)/(*T*_b_−*T*_lc_)|(Fristoe et al., 2015; Scholander et al., 1950). This assumption is often violated by conductance continuing to decline below the *T*_lc_, but we feel that the estimate approach best balances accuracy and viability. Most papers lack sufficient information to estimate conductance directly from metabolic data. We use units of oxygen consumption for metabolism and conductance to align with many reported rates and previous analyses (Fristoe et al., 2015). Due to the multiple parameters required for numerous species, we were unable to control for seasonal acclimation. A surprising number of papers do not report the seasonal timing of measurements.

Analysis (McKechnie, Coe, Gerson, & Wolf, 2016; Wolf, Coe, Gerson, & McKechnie, 2017) of the quality of the data compiled in Khaliq et al. (2014) identified issues with *T*_uc_ but not *T*_lc_ (see responses by Hof, Fritz, et al., 2017; Hof, Khaliq, Prinzinger, Böhning‐Gaese, & Pfenninger, 2017). Some *T*_uc_ data are of lesser quality due to small sample sizes or weak measurement protocols, so we only use the *T*_uc_ data for a coarse analysis of warm range boundaries mentioned in our discussion. We omitted *T*_uc_ measurements that were found to be of poor quality [“No UCT” or “NA‐” categories; we kept values based on low sample sizes due to the tentative nature of our analyses].

Diet, habitat, and nocturnality data were extracted from Elton Traits (Wilman et al., 2014). Data on whether a species uses torpor or hibernation were extracted from McNab (2008, 2009 ) and Ruf and Geiser (2015). A “torpor” trait was assigned a value of 1 if the species uses either torpor or hibernation and 0 otherwise. Data on relevant thermoregulatory traits such as body shape, insulation, and fur or feather properties were inadequate to include the traits in the analysis.

### 2.2 BMR data quality

We revisited the source papers to assess whether the T_lc_ data were calculated from valid BMR measurements. Quality criteria were selected in consultation with several physiologists as those most likely to be problematic in the initial data compilations. We used the following criteria to assess data quality for BMR: Measurements were made during the rest phase on inactive individuals in a postabsorptive state. We additionally recorded whether individuals measured were field‐collected (or the first generation reared in a laboratory or zoo in a small number of cases) and the location of field collection, as individuals collected far from the range boundary may lack adaptations and acclimation present near the range boundary.

In our full dataset for mammal ME_CRB_, the following proportions of species with data met our BMR quality control criteria: 93.9% [58.6% including NA (not available) values as not meeting quality criteria] were measured during the resting phase, 70.0% (42.9% including NA values) were postabsorptive, and 81.2% were wild‐caught (70.0% including NA values) (Table S2). Of the quality criteria, only whether the mammal species was live‐trapped or captive was a significant predictor of ME_CRB_ (resting phase: *F*_[1,82]_ = 1.53, *p* = 0.22; postabsorptive: *F*_[1,82]_ = 0.04 *p* = 0.84; wild caught: *F*_[1,82]_ = 5.15, *p* < 0.05). However, restricting the dataset to wild‐caught species does not substantially alter the peak value of metabolic expansibility (peak = 3.27, mean = 4.72, median = 3.75). The trait predictors of ME_CRB_ remain similar when considering only wild‐caught individuals (Table S3).

In our full dataset for bird ME_CRB_, the following proportions of species with data met our BMR quality criteria: 93.0% (86.9% including species without data) were measured during the resting phase, 88.9% (52.4% including species without data) were postabsorptive, and 68.9% were wild‐caught (50.8% including species without data) (Table S2). Similar to mammals, of the quality criteria only whether the bird species was live‐trapped or captive was a significant predictor of ME_CRB_ (resting phase: *F*_[1,32]_ = 0.00, *p* = 0.94; postabsorptive: *F*_[1,32]_ = 0.68, *p* = 0.42; wild caught: *F*_[1,32]_ = 6.90, *p* < 0.05). However, restricting the dataset to wild‐caught species did not substantially alter the peak value of ME_CRB_ (peak = 2.60, mean = 3.07, median = 3.11). The trait predictors of ME_CRB_ remained similar, but some predictors lose significance, when considering only wild‐caught individuals (Table S3).

The measured individuals were collected throughout the species’ range with average positions near the center of the range for both mammals (median and mean from range edge: 10.3° and 13.4° latitude, 47.2% and 49.4% of the species’ latitudinal range) and birds (median and mean from range edge: 18.3° and 20.4° latitude, 50.8% and 49.9% of the species’ latitudinal range). Neither distance metric is a significant predictor of ME_CRB_ in mammals (distance: *F*_[1,142]_ = 0.27, *p* = 0.60; percent: *F*_[1,142]_ = 0.10, *p* = 0.76) or birds (distance: *F*_[1,29]_ = 0.12, *p* = 0.73; percent: F_[1,29]_=0.39 *p* = 0.54). Collection locations have a median elevation of 275 m (25th and 75th quantiles: 39 to 849 m, based on collection coordinates and Google Maps Elevation API). Thus, few of the physiological measurements reflect metabolic adaptation to high elevation.

### 2.3 Analyses

We examine the distribution of ME_CRB _estimates across bird and mammal species to assess evidence for a metabolic constraint. We assessed skewness and kurtosis of the ME_CRB_ distribution using the skewness metric and D'Agnostino skewness test and Geary metric and Bonett‐Seier test in the R moments package. We tested for unimodality in the distributions using Hartigans’ dip statistic in the R diptest package. To test whether ME_CRB_ varies systematically with *T*_min_ or *T*_max_, we constructed null models for ME_CRB_ by randomizing *T*_min_ or *T*_max_ among species and calculating the median and mean ME_CRB_ values. We repeated the randomization 1,000 times.

We then used regressions to assess whether species’ traits indicating adaptation to or evasion of cold temperatures can explain variation in ME_CRB_. We used model selection based on AICc (dredge function) and model averaging (model.avg function in R package MuMIn) to conclude that the best models omitted interactions between the predictor variables (mass, diet, nocturnality, torpor). Accounting for phylogeny did not alter our results, so we report phylogenetic analyses in Appendix S1.

We used thermal isoclines (consistent with species maintaining a constant ME_CRB_ in the absence of acclimation or adaptation) to project species’ cold range boundaries in both current and future environments. For graphical purposes, we used observed west and east longitudinal extents to depict distributions. We identified as thermally habitable all pixels with *T*_min_ warmer than the predicted physiological lower temperature limit (based on species‐specific observed ME_CRB_). We subsequently removed pixels that were geographically isolated from other thermally habitable pixels using the clump function in the R package raster. We omitted all clumps with areas less than 5% of the area of the largest clump, because the core of the predicted distribution is most representative of latitudinal extents. We further restricted our predicted distribution to clumps overlapping with the latitudinal extent of the observed species range. We then quantified the latitude of the cold range boundary as the median latitude of grid cells along the cold range edge.

## 3 RESULTS

### 3.1 Metabolic expansibility at the cold range boundary

**Figure 2:**
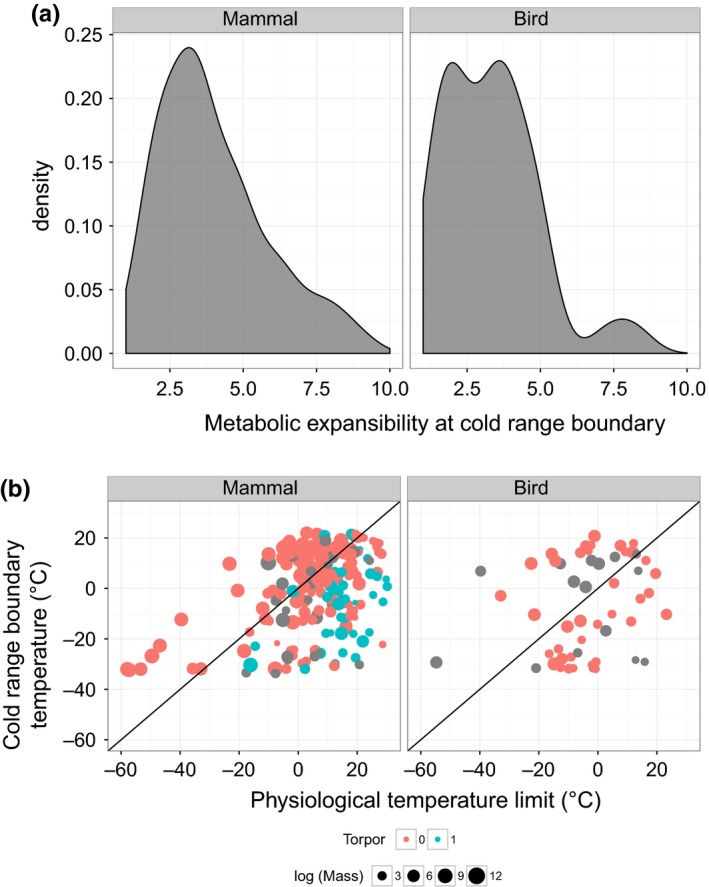
The density distribution of metabolic expansibility, ME_CRB_ (the factor by which metabolic rate at the cold range edge is elevated over basal metabolic rate) peaks at similar values for birds and mammals (a). We examine interspecific variation in ME_CRB_ by (b) plotting the physiological temperature limit predicted by assuming the mode of ME_CRB_ (*x*‐axes) and the observed temperatures at the cold range boundaries (*y*‐axes). Mammals and birds that are small (symbol size) and use torpor or hibernation (color, 1 = use, gray = no data) tend to be found in environments colder than predicted assuming the mode ME_CRB_ (i.e., they have higher ME_CRB_). The lines indicate 1:1 relationships.

Our analysis of bird and mammal species with disparate geographic distributions, habitats, traits, and life histories suggests that winter temperatures and the ability to elevate metabolism to maintain body temperatures constrain many cold range boundaries (Figure 2). Cold range boundaries of both mammals (*SD* = 4.6°, mad = 3.9° of median *T*_min_) and birds (*SD* = 4.6°, mad = 3.3° of median *T*_min_) approximately follow temperature isoclines (Figure S1 in the Supporting Information). Translating this thermal variability into metabolic consequences using the Scholander‐Irving model, the median standard deviations in cold range boundary temperatures represent a change in MR_CRB_ estimates of 12.4% ± 12.2% (mean ± *SD*) for mammals and 10.8% ± 7.8% for birds.

The distributions of metabolic expansibility, ME_CRB_, are peaked and peaks occur at similar values for birds and mammals. The bird distribution has a slight dip at the peak of the density distribution, which we attribute to limited sample size in the absence of evidence for non‐unimodality (Hartigans’ dip test: *D* = 0.05, *p* = 0.3). We thus estimate the peak value as the mean of the two subpeaks. The density distribution of ME_CRB_ peaks at 2.72 for birds (median = 3.21, mean ±*SD* = 3.28 ± 1.63) and at a somewhat higher value (3.17, median = 3.63, mean ±*SD* = 4.64 ± 3.35) for mammals. ME_CRB _values fall outside the 95% confidence intervals of the null model estimated by randomization for both mammals (median: 3.53–3.54, mean: 4.55–4.56) and birds (median: 2.63–2.64, mean: 3.16–3.17). The previous value (ME_CRB_ = 2.5) found for birds (Root, 1988) was similar to our estimate of the peak of the distribution.

**Figure 3:**
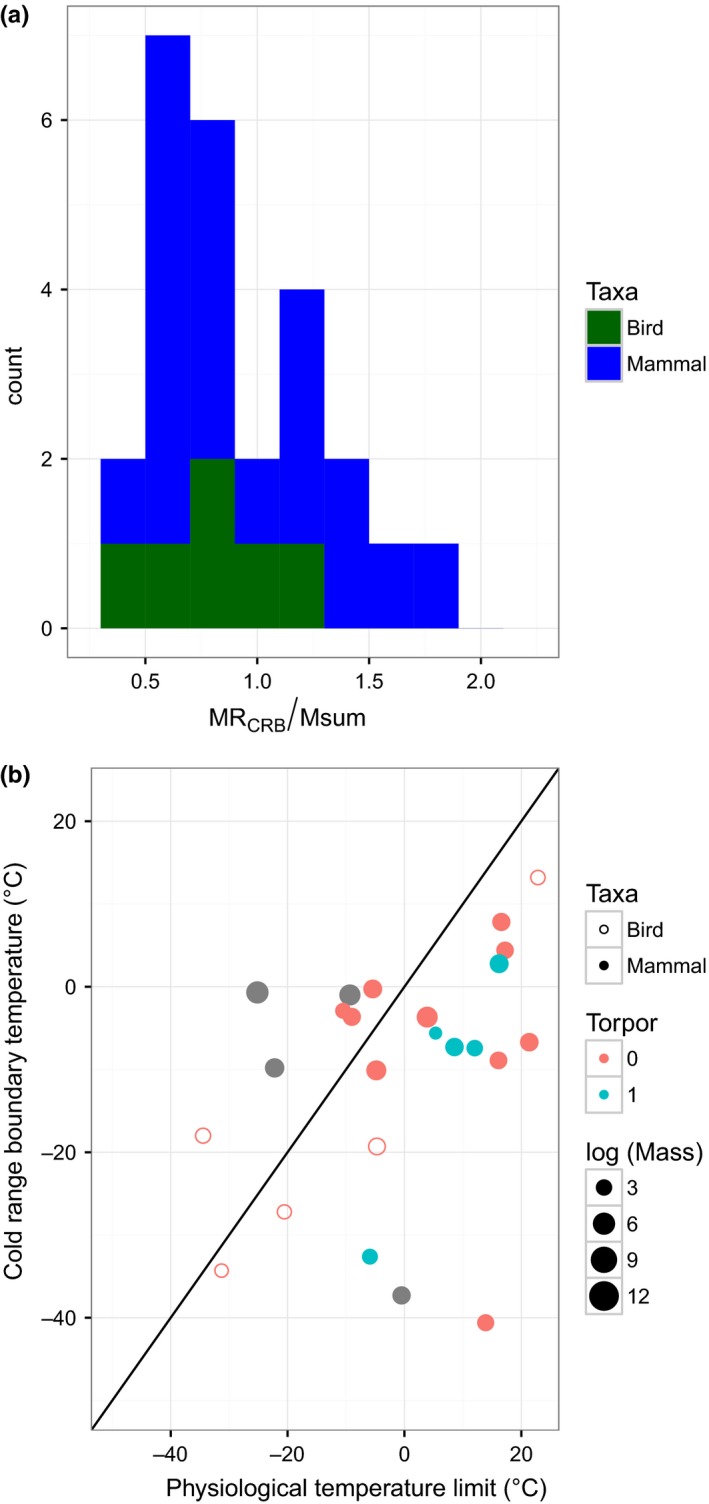
A histogram (a) of the ratio of summit metabolic capacity (M_sum_) to estimated metabolic rate at the cold range boundary (MR_CRB_) suggests the high energetic demands of thermoregulation. We examine interspecific variation in the ratio (MR_CRB_/M_sum_) by plotting the observed temperatures at the cold range boundaries against the physiological temperature limit corresponding to MR_CRB_ = 0.7 M_sum_ (b). We depict mammals (filled circles) and birds (hollow circles), mass (symbol size), and use of torpor or hibernation (color, 1 indicates use).

We assessed whether ranges may be constrained more strongly by maximum metabolic capacity (M_sum_) rather than the factorial capacity for elevating metabolism over BMR (ME_CRB_). Among the limited data available for our focal species (*N* = 20 mammal and 6 bird species), M_sum_ is on average 5.0 times BMR (median 5.4, 25th to 75th percentile = 4.0 to 6.3). The density distribution of the ratio MR_CRB_/M_sum_ peaks at 0.7 (median = 0.88, mean ± *SD* = 0.96 ± 0.44, Figure 3).

The right skewed distributions of ME_CRB_ (Figure 2) suggest that some species have evolved the capacity to maintain a higher ME_CRB_ or to evade the constraints of cold temperatures via torpor, microclimate selection, or movement. Estimate error likely also contributes to the skew, but if so, removing the error would strengthen evidence for a metabolic constraint. The distribution for mammals is more skewed (skewness = 2.54) than that for birds (skewness = 1.06), but both exhibit significant positive skew (D'Agnostino test, mammals: *z* = 9.52, *p* < 10^−15^; birds: *z* = 3.22, *p* < 0.001). Only mammals exhibit significantly more kurtosis than expected under normality (Bonett‐Seier test, mammals: Geary metric: 0.78, *z* = 10.20, *p* < 10^−15^; birds: Geary metric: 0.66, *z* = 0.78, *p* = 0.2).

We next assess whether traits that allow organisms to maintain high metabolism or evade cold temperature contribute to the skewed distribution. Mammalian traits (mass, diet, nocturnality, and use of torpor or hibernation) account for a substantial portion of variation in ME_CRB_ (*r* ^2^ = 0.29, *F*_[7,171]_ = 11.2, *p* < 0.001); mammal species that are relatively small (*t* = −4.36, *p* < 0.001) and use torpor or hibernation (*t* = 4.99, *p* < 0.001) tend to have higher ME_CRB_ (Figure 2, Table S1). Diet also significantly influences ME_CRB_, with granivores having higher ME_CRB_ than mammals consuming other diets (*F* = 2.44, *p* < 0.05, ANOVA, Table S3). Bird traits (mass, diet, and nocturnality) likewise account for a substantial portion of variation in ME_CRB_ (*r* ^2^ = 0.28, *F*_[6,54]_ = 4.9, *p* < 0.001); birds that are small (*t *= −2.82, *p* < 0.05) tend to have higher ME_CRB_. Birds that eat invertebrates or plants and seeds exhibit higher ME_CRB_ than those consuming other diets (*F* = 7.85, *p* < 0.01, ANOVA). Limited phylogenetic signal in mammal and bird ME_CRB_ (Figure S3) arises largely from conservatism of predictor traits (Appendix S1). Phylogenetic regressions do not substantially deviate from linear regressions (Table S1, Appendix S1).

### 3.2 Range shifts

**Figure 4:**
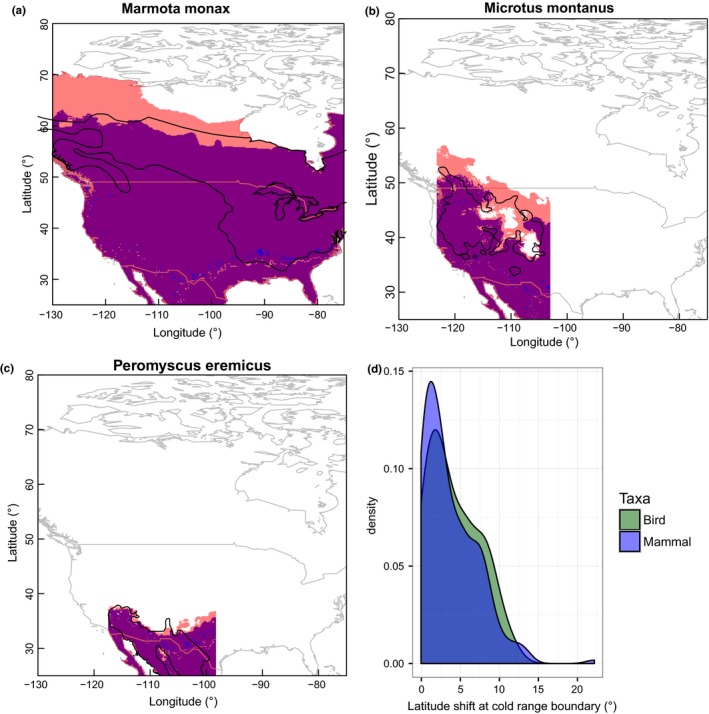
We depict observed cold range boundaries (CRB, black polygons: IUCN range maps) and those projected based on metabolic constraints for exemplar North American rodents in current (blue: 1950–2000) and predicted future (pink: 2061–2080 from HadGEM2‐AO model) climates (a–c). Purple shading indicates portions of the projected range occupancy that persists through climate warming. We note few areas of range contraction (blue) since we are only predicting CRBs (the depicted equatorward extent is not meaningful). We restrict our CRB projections to the observed longitudinal extent. The species differ in the extent of their current distribution and the projected CRB shift resulting from climate change (a, *Marmota monax*, groundhog; b, *Microtus montanus*, montane vole; and c, *Peromyscus eremicus*, cactus mouse). Projections based on metabolic constraints indicate that the majority of mammals (purple) and birds (green) will shift their CRB modestly poleward through climate changes (d). However, numerous species are projected to shift their CRB poleward by 10° latitude and some species are projected to shift by as much as 22°

We forecast potential range shifts by examining how metabolic constraints will shift through climate change. For example, North American rodent species differ in their metabolic constraints, the extents of their current distribution, and the projected range expansion as a result of climate change (Figure 4 for projections using the HadGEM2‐AO model; Figure S4 for CCSM4 model projections). The quality of the range projections varies across species (Figures S5–S8). We predict that most mammals and birds will shift their cold range boundaries poleward through climate changes (Figure 4). We project a similar magnitude of cold range boundary shifts for mammals (mean = 3.77°, median = 2.58°) and birds (mean = 4.20°, median = 3.63°). Numerous species are projected to shift their cold range boundary poleward by 6° latitude (75% quantile), and some species are predicted to shift by as much as 22° (Figure 4).

## 4 DISCUSSION

Our data are consistent with the poleward range edges of both birds and mammals being constrained by the factor by which they can elevate their metabolism above basal rates (perhaps resulting from a constraint on maximum metabolic rates). The constraint may result from either direct physiological limitations on metabolism, such as the ability to sustain high rates of thermogenesis over prolonged periods, or limitations on energy acquisition. The rates of ME_CRB_ that we find for birds (peak of distribution = 2.7) are similar to a previous value (2.5) for a more taxonomically and geographically restricted analysis (Root, 1988). We find a somewhat higher peak of the ME_CRB_ distribution for mammals (3.2). Our analysis supports metabolic constraints as a mechanism underlying observations that endotherms track thermal isotherms through climate change (Chen, Hill, Ohlemüller, Roy, & Thomas, 2011; Tingley, Monahan, Beissinger, & Moritz, 2009). However, many observed range shifts have been idiosyncratic in extent and direction (Gibson‐Reinemer & Rahel, 2015). Filtering the range shifts through the lens of metabolic constraints may resolve some discrepancies.

The distribution of ME_CRB_ is right skewed, more so for mammals than for birds. The greater skew in the mammal ME_CRB_ distribution is consistent with the prominent use of hibernation and protected microclimates (e.g., burrows, dens, subnivean space) during winter in mammals, but lesser use of these options to avoid cold thermal environments in birds (Ruf & Geiser, 2015; Swanson, 2010). These adjustments have the effect of rendering the thermal conditions encountered at the ME_CRB_ as less extreme than the actual ambient conditions, which results in an overestimation of the thermal isocline followed by the cold range boundary. In addition, differences in the mechanisms of thermoregulation between mammals and birds may contribute to the difference in ME. Cold‐adapted mammals have well developed capacities for non‐shivering thermogenesis through brown fat, but birds lack brown fat and although they may possess some muscular non‐shivering thermogenesis, muscular shivering appears to be the primary mechanism of heat production in birds (Mezentseva, Kumaratilake, & Newman, 2008).

The limited data on maximum cold‐induced metabolic capacity (M_sum_) provide additional support for a metabolic constraint. We estimate that thermoregulation at the cold range boundary requires a substantial proportion (>50%) of the potential metabolic capacity for thermogenesis of the species. This supports the existence of a metabolic constraint on range boundaries and suggests that species use a substantial portion of their maximum metabolic capacity to thermoregulate. The right skewed distribution (and instances where MR_CRB_/M_sum_ > 1) suggests that some species use torpor or hibernation or evade the coldest temperatures through habitat and microclimate selection (Figure 3). Because M_sum_ is a flexible trait correlated with environmental conditions (Rezende et al., 2004; Swanson, 2010), ratios approaching or exceeding one may also result from M_sum_ measurement occurring for populations in warmer climates than those at the cold range boundary. Correlations between M_sum_ and environmental temperatures have been previously documented for rodents (Bozinovic et al., 2011; Rezende et al., 2004) and birds (Stager et al., 2015; Swanson, 2010).

We identify traits associated with high values for ME_CRB_, which may be adaptations to or consequences of inhabiting cold environments. Body mass is an important factor that influences ME_CRB_. Smaller mammals, which tend to exhibit greater ME_CRB_, may be able to evade cold temperatures through seeking shelters or selecting favorable microclimates. Alternatively, the ability to use torpor or hibernation enables mammals to inhabit colder environments. Mammals using torpor tend to be small, which may contribute to the relationship between mass and ME_CRB_ (Ruf & Geiser, 2015). Small mammals may also be able to meet the resource requirements or store energy to maintain high metabolism through cold periods (due to the low per‐organism, or total, metabolic rate stemming from their small size) (Angilletta, Cooper, Schuler, & Boyles, 2010; Humphries et al., 2004). Mammals at lower trophic levels (herbivores and invertebrate consumers) tend to exhibit higher ME_CRB_. These species tend to have lower BMR (McNab, 2008) and their food sources may be more consistently available.

Lower mass‐specific rates of heat production and heat loss (conductance) and smaller surface area to volume ratios favor larger body sizes in colder environments (i.e., Bergmann's hypothesis, Ashton, Tracy, & Queiroz, 2000). Regardless, birds’ and mammals’ body sizes are diverse across climates (Fristoe et al., 2015). An analysis of regression residuals suggests that adaptations to cold environments in birds and mammals results in increased BMR and reduced conductance (Fristoe et al., 2015). Our analysis suggests that greater values of ME_CRB_ (perhaps associated with selection for higher M_sum_) enable small birds and mammals to inhabit cooler environments. Birds from cold climates tend to exhibit higher M_sum_ (Stager et al., 2015). We identify traits (small body size, use of torpor or hibernation, diet) that may enable the elevated ME_CRB_.

Because our analysis is motivated, in part, by a desire to develop mechanistic and general approaches to predict endotherm ranges, we discuss the limited viability of using metabolic constraints to predict warm range boundaries. We omit a full analysis of warm range boundaries because we estimated that 61% and 45% of mammal and bird species with unconstrained warm range boundaries, respectively, do not experience *T*_max_ values exceeding their *T*_uc_. We note that these values are likely an overestimate because they do not account for heat associated with solar radiation or heat extremes, but they do suggest a greater viability for using metabolic constraints to project cold range boundaries. Our estimates of metabolic expansibility at the warm range boundary (for species with *T*_max_ > *T*_uc_, following methodology for ME_CRB_) approximate 1 (Figure S2), highlighting the physiological challenges of heat dissipation (Weathers, 1981). At warm range boundaries, the capacity for evaporative cooling may be more limiting than the associated metabolic costs and minimal endogenous heating is favored (McKechnie, Whitfield, et al., 2016; Tieleman & Williams, 2000). Evaporative cooling poses a risk of dehydration in response to short term heat stress (McKechnie, Hockey, & Wolf, 2012) and presents a challenge for longer term water balances (Kearney et al., 2016). Additionally, other biotic factors such as species interactions and resource or habitat constraints often constrain warm range boundaries (Sexton, McIntyre, Angert, & Rice, 2009). Range contractions at warm range boundaries may primarily result from indirect effects (e.g., species’ interactions), which often predominate in climate change responses (Tylianakis, Didham, Bascompte, & Wardle, 2008; Walther, 2010).

Assuming species follow thermal isoclines due to metabolic constraints, we project that species will shift their cold range boundaries poleward by an average of 3.9° latitude with numerous species shifting by 6° (75% quantile). Our analyses suggest that hibernation and torpor are important determinants of cold range boundaries. Climate change will also likely alter the energetics of hibernation, which may amplify poleward range shifts (Humphries, Thomas, & Speakman, 2002). Many bird and mammal species rely on seasonal migration to obtain resources to meet seasonal energetic demands; considering the costs and benefits of such movements will be important to forecasting responses to climate change among migratory birds and mammals (which we excluded from our analysis) (Robinson et al., 2009). Shifting activity times may also function to modify estimates of range shifts (Levy, Dayan, Kronfeld‐Schor, & Porter, 2012).

Our analysis of a taxonomically and geographically diverse dataset suggests that metabolic constraints provide a viable mechanism for projecting the poleward range boundaries of endotherms. However, estimating metabolic constraints is hindered both by parameter uncertainty and by the many adaptations organisms employ to evade the constraints (Fuller et al., 2016; Mitchell et al., 2018). The Scholander‐Irving model we employ provides a tractable approximation of metabolic constraints, but we highlight ways that refining metabolic estimates could improve upon the analyses. We estimate metabolic costs assuming homeothermy, but many studies highlight that endotherms exhibit a continuum of heterothermy (Boyles et al., 2013; Levesque, Nowack, & Stawski, 2016). Consideration of the occurrence of torpor/hibernation in the present study only partially accounted for deviations from thermoregulation due to *T*_b_ variation. Many endotherms seasonally acclimatize their insulation, behavior, and physiology (Boyles et al., 2011; Bozinovic et al., 2011). Many metabolic estimates in our database are specific to the cold season, but data limitations prevented fully accounting for acclimatization. A comparison of BMR and field metabolic rates (FMR) for small mammals failed to find support for intrinsic limitations on metabolism and low FMRs in very cold climates indicated acclimatization including behavioral avoidance (Humphries et al., 2005). Over longer time periods, adaptation may alter morphology or metabolic constraints (Boyles et al., 2011). Behavioral strategies for buffering cold include sheltering, huddling, basking, and microclimate selection (Angilletta et al., 2010). Resource availability may constrain metabolism more strongly than physiology. Despite these complications that introduce variability to estimates of metabolism and should flatten out the distribution of ME_CRB_, we find peaked ME_CRB_ distributions that suggest metabolic constraints on poleward range boundaries.

Our analysis suggests that metabolic constraints can provide an initial step toward generalizable and mechanistic projections of endotherm responses to climate change. Revisiting the simple, but potentially powerful, approach of Root (1988) may improve predictive models of endotherm distributions and distribution shifts, many of which are based on inaccurately assuming the environmental niche of endotherms is bound by their TNZ (Mitchell et al., 2018). Even simple metabolic models may alleviate some misconceptions of endotherm thermal physiology underlying predictions of climate change responses (Mitchell et al., 2018) and inform the development of more sophisticated and accurate models. Our identification of traits that significantly influence ME_CRB_ estimates points to factors to include in improved models. Concerns over the quality of physiological (primarily *T*_uc_) data (McKechnie, Coe, et al., 2016; Wolf et al., 2017) highlight the need for additional physiological data collection and compilation to further model development. A wide divide currently exists between the detailed considerations physiologists employ when predicting responses to climate change for particular species and the general approaches employed by ecologists to predict responses across many endothermic species. Our analysis suggest that further consideration and testing of metabolic constraints may help close the divide.

## CONFLICT OF INTEREST

None declared.

## AUTHOR CONTRIBUTIONS

All authors designed the study and wrote the manuscript. LBB and IK compiled data. LBB performed the analyses, interpreted results, and led manuscript writing. IK led the data quality assessment.

## DATA ACCESSIBILITY

Data available from the Dryad Digital Repository: https://doi.org/10.5061/dryad.68fr52p. Data and scripts for the analysis are also available at github.com/lbuckley/tnz.
