# Supplementary material for: Does metabolism constrain bird and mammal ranges and predict shifts in response to climate change?
Source: Ecol Evol. 2018 Dec 10;8(24):12375–85. doi: 10.1002/ece3.4537 (PMC6308872; doi:10.1002/ece3.4537)
Supplement: Supplementary file 1 [file ECE3-8-12375-s001.pdf]

SUPPLEMENTAL MATERIAL

Appendix S1. Phylogenetic analysis

Methods

Phylogenetic data were compiled from published supertrees of birds and mammals (Fritz, Bininda-Emonds, & Purvis, 2009; Jetz, Thomas, Joy, Hartmann, & Mooers, 2012; Kuhn, Mooers, & Thomas, 2011). To remove polytomies in the mammal tree, we adopted an approach (Kuhn et al., 2011) which uses a birth-death model of diversification at the polytomies and generates a pseudo-posterior distribution of resolved trees. To test the robustness of the analysis across different potential phylogenetic trees compiled from the published supertrees, we repeated the analyses for 100 trees from the pseudo-posterior distribution of the mammalian as well as the avian tree. For the final analysis we generated a maximum clade credibility (MCC) tree, using TreeAnnotator (included in BEAST v.1.7.5, Drummond & Rambaut, 2007).

We quantified phylogenetic signal in ME_{CRB} and predictor traits (body mass, torpor use, nocturnality) using Blomberg's K (R function phylosignal from picante package)(Kembel et al., 2010) and pagel's λ (R function phylosig from phytools package)(Revell, 2011). We additionally assessed phylogenetic signal in the residuals of linear regressions (Revell, 2010). K is a ratio of variance among species to variance in contrasts that has an expectation of K=1 for a trait that evolved by Brownian motion. Pagel's λ scales the correlations among species to the correlation expected under Brownian motion. Values range between 0 (no correlation) to 1 (correlation corresponding to Brownian motion). We assessed significance of K by comparing the variance of independent contrasts for 1000 randomized (tip-swapped) trees with that of the observed trees. We used the contMap function from the R phytools package (Revell, 2011) to plot patterns of trait conservatism across phylogenies.

We examined the predictors of ME_{CRB} using phylogenetic generalized least squares (PGLS) with several variants of correlation matrices accounting for phylogeny (Garamszegi, 2014). We first fit λ simultaneously with the PGLS regression (Garamszegi, 2014; Revell, 2010) (R corPagel function from the ape package). We used ANOVAs to compare PGLS regressions using the fitted λ to regressions with λ=0 (phylogenetic independence) or λ=1 (trait evolution according to Brownian motion) (Garamszegi, 2014). We additionally report the results of PGLS regressions incorporating phylogenetic correlation structures assuming trait evolution under Brownian motion or Martins and Hansen's (1997) model (R corBrownian, corPagel, and corMartins functions from the ape package) (Paradis, Claude, & Strimmer, 2004).

Results

ME_{CRB} shows generally weak conservatism across the phylogenies, but the predictor traits are highly conserved (Figure S2). At the cold range boundary in mammals (N=179), we detect weak to moderate phylogenetic signal in ME_{CRB} using Blomberg's K (K=0.19, z=-1.03, P=0.06) or Pagel's λ (λ=0.56, LogLik= -468.5). The residuals of the linear regression (ME_{CRB}~ mass + diet + nocturnality + torpor) show less phylogenetic signal (K=0.13, z=-0.27, P=0.47; λ=0.09, LogLik= -444.5), consistent with the phylogenetic signal arising due to conserved traits. Mass (K=0.48, z= -0.80, P<0.05; λ= 1.0, LogLik= -1907.5), nocturnality (K=0.62, z= -3.44, P=0.001; λ= 1.0, LogLik= 14.1), and torpor (K=0.78, z= -4.66, P=0.001; λ= 1.0, LogLik= -4.5) all exhibit phylogenetic signal.

Accounting for phylogeny yields similar regression results for mammal ME_{CRB}. We estimate λ=0.28 (95% CI= -0.01 to 0.56) in the PGLS regression (table S1). Using ANOVAs to compare PGLS regressions under the range of λ assumptions rejects both phylogenetic independence (L.Ratio=5.13, p<0.05) and trait evolution under Brownian motion (L.Ratio=112.3, p<0.0001). Mass, diet, and torpor remain significant predictors of ME_{CRB} in most PGLS models (table S1).

Phylogenetic conservatism in bird ME_{CRB} arises, at least in part, from the conservatism of traits. This finding may be an artifact of small sample size (N=49). Mass (K=0.67, z= -0.60, P=0.001; λ= 1.0, LogLik= -320.9) and nocturnality (K=3.71, z= -0.64, P=0.001; λ= 1.0, LogLik= 39.0) are strongly conserved. Limited evidence of phylogenetic conservatism is found for either ME_{CRB} (K=0.38, z= -0.60, P=0.01; λ= 0.75, LogLik= -85.7) or the ME_{CRB} residuals from a linear regression (K=0.30, z=-0.49, P=0.09; λ= 0.00, LogLik= -77.0). However, fitting λ in the PGLS regression estimates λ=0.99 (95% CI= 0.96 to 1.02). ANOVAs comparing PGLS models reject both phylogenetic independence (L.Ratio 6.23, p=0.01) and trait evolution under Brownian motion (L.Ratio 6.84, p=0.009).

References

Drummond, A. J., & Rambaut, A. (2007). BEAST: Bayesian evolutionary analysis by sampling trees. *BMC Evolutionary Biology*, *7*(1), 214.

Fritz, S. A., Bininda-Emonds, O. R., & Purvis, A. (2009). Geographical variation in predictors of mammalian extinction risk: big is bad, but only in the tropics. *Ecology Letters*, *12*(6), 538–549.

Garamszegi, L. Z. (2014). Modern phylogenetic comparative methods and their application in evolutionary biology. *Concepts and Practice. London, UK: Springer*. Retrieved from http://link.springer.com/content/pdf/10.1007/978-3-662-43550-2.pdf

Jetz, W., Thomas, G. H., Joy, J. B., Hartmann, K., & Mooers, A. O. (2012). The global diversity of birds in space and time. *Nature*, *491*(7424), 444–448.

Kembel, S. W., Cowan, P. D., Helmus, M. R., Cornwell, W. K., Morlon, H., Ackerly, D. D., … Webb, C. O. (2010). Picante: R tools for integrating phylogenies and ecology. *Bioinformatics*, *26*(11), 1463–1464.

Kuhn, T. S., Mooers, A. Ø., & Thomas, G. H. (2011). A simple polytomy resolver for dated phylogenies. *Methods in Ecology and Evolution*, *2*(5), 427–436.

Martins, E. P., & Hansen, T. F. (1997). Phylogenies and the comparative method: a general approach to incorporating phylogenetic information into the analysis of interspecific data. *American Naturalist*, *149*, 646–667.

Paradis, E., Claude, J., & Strimmer, K. (2004). APE: analyses of phylogenetics and evolution in R language. *Bioinformatics*, *20*(2), 289–290.

Revell, L. J. (2010). Phylogenetic signal and linear regression on species data. *Methods in Ecology and Evolution*, *1*(4), 319–329.

Revell, L. J. (2011). phytools: an R package for phylogenetic comparative biology (and other things). *Methods in Ecology and Evolution*, *3*, 217–223.

Table S1. Results of linear (left) and phylogenetic generalized least squares (PGLS, right) regressions examining predictors of metabolic expansibility. We examine the predictive ability of traits: mass (g), diet (invertebrates; omnivores; plants and seeds; vertebrates, fish, and scavenging; estimates relative to frugivory or nectarivory), nocturnality (1 for nocturnal, 0 otherwise), and torpor / hibernation (1 for species that use torpor or hibernation, 0 otherwise). We omit torpor / hibernation for birds because no included species exhibit torpor or hibernation. The PGLS regressions incorporate the following correlation structures (left to right): Pagel's λ, trait evolution according to Brownian motion, and Martins and Hansen's (1997) model.

	Linear Model				PGLS- Pagel				PGLS-Brownian				PGLS- Martins			
	Estimate	SE	t	P	Estimate	SE	t	P	Estimate	SE	t	P	Estimate	SE	t	P
**Mammal metabolic expansibility scope at cold range boundary**																
log(mass)	-0.46	0.11	-4.36	0.000 ***	-0.57	0.13	-4.51	0.000 ***	-0.96	0.25	-3.81	0.000 ***	-0.46	0.11	-4.34	0.000 ***
diet- invertebrate	1.79	0.87	2.05	0.042 *	1.51	0.92	1.64	0.102	-0.39	1.73	-0.22	0.823	1.70	0.89	1.90	0.059
diet- omnivore	1.22	0.88	1.38	0.168	1.14	0.90	1.26	0.210	0.77	1.66	0.47	0.642	1.14	0.90	1.26	0.208
diet- plant seed	1.88	0.81	2.32	0.022 *	1.85	0.92	2.02	0.045	1.39	1.79	0.78	0.438	1.82	0.83	2.18	0.031 *
diet- vert fish scav	0.65	1.11	0.58	0.563	0.42	1.11	0.38	0.703	0.90	1.83	0.49	0.623	0.58	1.14	0.51	0.609
nocturnal	0.81	0.68	1.19	0.237	1.06	0.74	1.43	0.154	1.37	1.39	0.99	0.324	0.83	0.68	1.22	0.226
torpor / hibernation	2.83	0.57	4.99	0.000 ***	2.57	0.61	4.23	0.000 ***	1.05	1.24	0.84	0.400	2.86	0.57	5.00	0.000 ***
	AIC=907.3	r^2= 0.29	$F_{[7,171]}$=11.2	p=10^{-11}	AIC=908.2		logLik= -444		AIC=1029		logLik= -506		AIC=908.6		logLik= -444	
**Bird metabolic expansibility at cold range boundary**																
log(mass)	-0.35	0.15	-2.28	0.026 *					-0.65	0.27	-2.42	0.020 *				
diet- invertebrate	1.94	0.65	2.99	0.004 **					0.71	0.85	0.83	0.409				
diet- omnivore	1.08	0.61	1.76	0.084					0.53	0.64	0.84	0.406				
diet- plant seed	2.00	0.53	3.77	0.000 ***					1.98	0.68	2.91	0.006 **				
diet- vert fish scav	1.28	0.87	1.47	0.147					2.24	0.77	2.92	0.006 **				
nocturnal	0.30	0.73	0.41	0.687					0.42	1.78	0.23	0.816				
	AIC=220.8	r^2= 0.28	$F_{[6,54]}$=4.9	p<0.001					AIC=177.7		logLik= -80.8					

Table S2. A summary of the quality of BMR data. For mammals and birds, we present the number of species for which measurements were during the rest or active phase, measurements were taken on postabsorptive animals or not, and measurements were taken on individuals field trapped (or the first generation reared in a laboratory or zoo in a small number of cases) or captive. We present the mean and median of distances between the collection localities and the cold range boundaries in degrees (°) and as the percent of the latitudinal range extent (°). We also indicate (NA) the number of species for which we could not determine quality from the source publication.

	**Mammals**	**Birds**
active phase	8	4
rest phase	123	53
NA	81	4
postabsorptive	91	32
not postabsorptive	39	4
NA	82	25
trapped	148	31
captive	34	14
NA	30	16
from cold edge		
distance (°)		
mean	13.4	20.4
median	10.3	18.3
% of latitudinal extent		
mean	49.4	49.9
median	47.2	50.8
NA	34	12

Table S3. Results of linear generalized least squares regressions examining predictors of metabolic expansibility for live trapped mammals and birds. We examine the predictive ability of traits: mass (g), diet (invertebrates; omnivores; plants and seeds; vertebrates, fish, and scavenging; estimates relative to frugivory or nectarivory), nocturnality (1 for nocturnal, 0 otherwise), and torpor / hibernation (1 for species that use torpor or hibernation, 0 otherwise). We omit torpor / hibernation for birds because no included species exhibit torpor or hibernation.

	**Linear Model**				
**Live captured**	**Estimate**	**SE**	**t**	**P**	
**Mammal metabolic expansibility scope at cold range boundary**					
log(mass)	-0.32	0.13	-2.47	0.015	*
diet- invertebrate	1.05	0.93	1.13	0.261	
diet- omnivore	1.53	0.92	1.66	0.100	
diet- plant seed	2.45	0.89	2.75	0.007	**
diet- vert fish scav	0.99	1.18	0.84	0.404	
nocturnal	0.98	0.81	1.21	0.230	
torpor / hibernation	3.2543	0.6047	5.382	0.000	***
	AIC=622.9	r^2= 0.28	$F_{[7,119]}$=8.0	$p=10^{-7}$	
**Bird metabolic expansibility scope at cold range boundary**					
log(mass)	-0.37	0.23	-1.57	0.129	
diet- invertebrate	1.15	0.88	1.30	0.205	
diet- omnivore	0.86	0.97	0.89	0.384	
diet- plant seed	2.96	0.82	3.60	0.001	**
diet- vert fish scav	1.63	1.18	1.39	0.179	
nocturnal	1.02	0.82	1.25	0.224	
	AIC=112.7	r^2= 0.26	$F_{[6,24]}$=2.7	$p<0.05$	

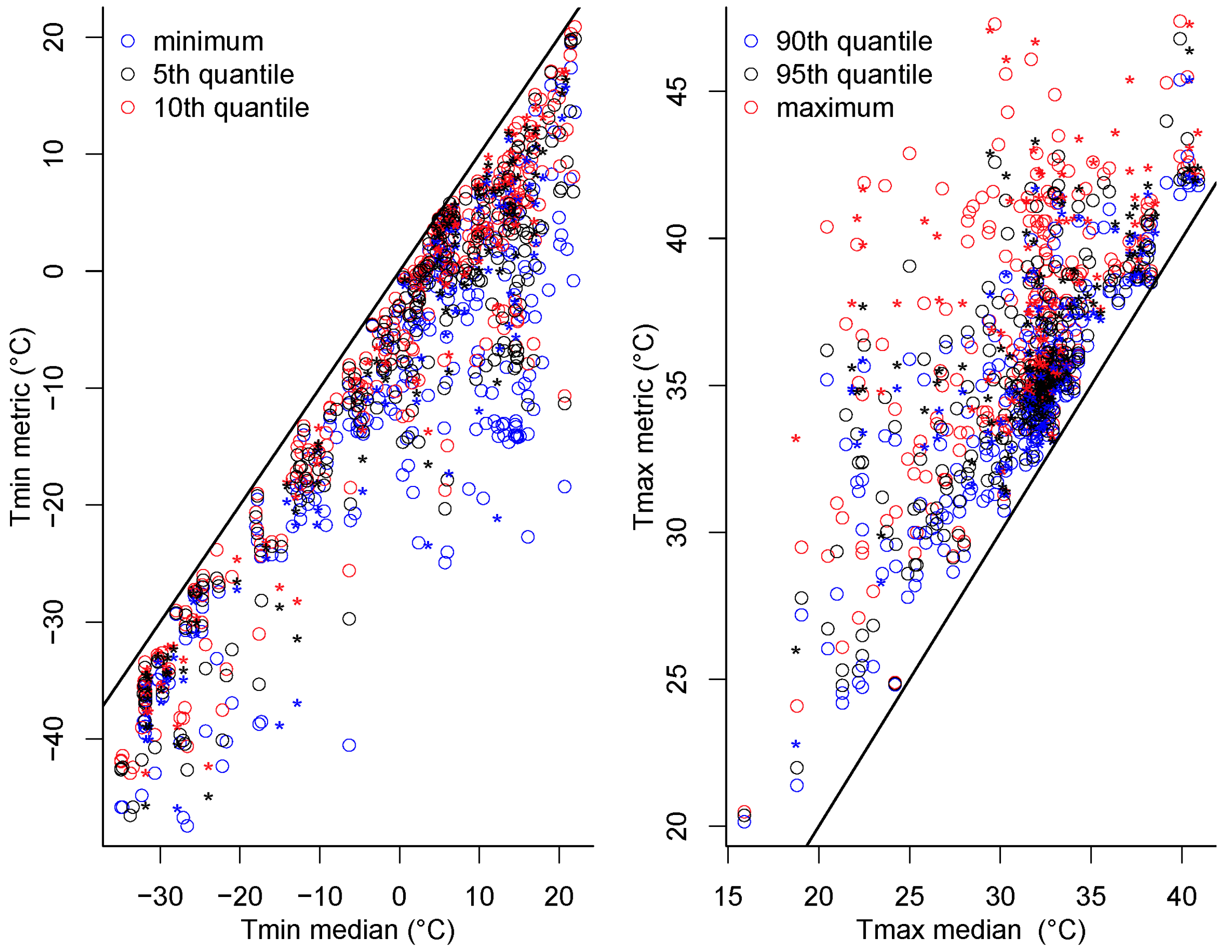

Figure S1. Cold and warm range boundaries approximately follow thermal isoclines for both mammals (o) and birds (*). We compare the minimum (Tmin) and maximum (Tmax) temperatures used in the analysis (medians) to other temperature metrics.

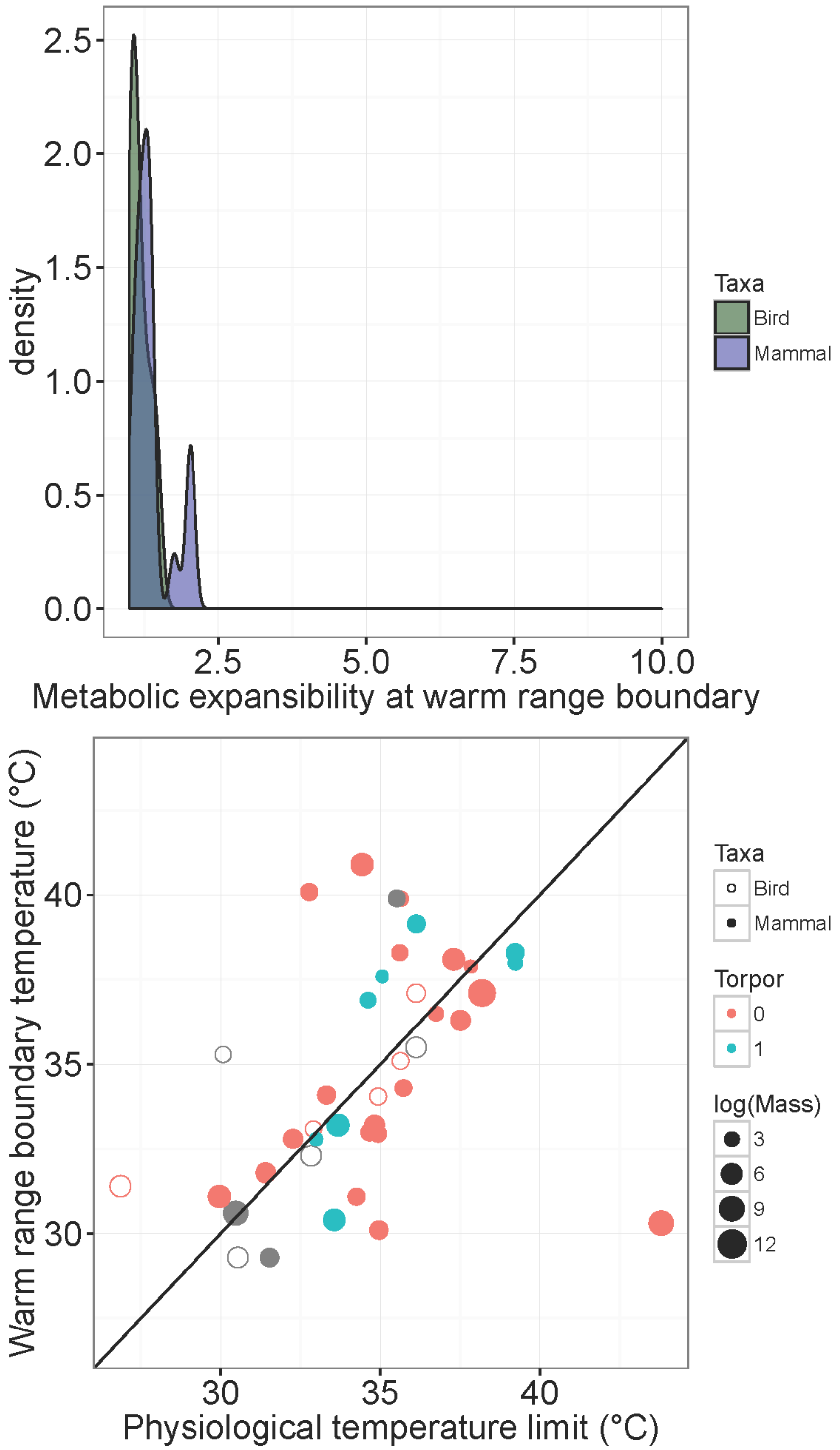

Figure S2. The density distributions of metabolic expansibility, ME_{WRB} (the factor by which metabolic rate at the warm range edge is elevated over basal metabolic rate) are narrow, peak at values near 1, and are similar for birds and mammals. We examine interspecific variation in ME_{WRB} by plotting the physiological temperature limit predicted by assuming the mode of ME and the observed temperatures at the warm range boundaries.

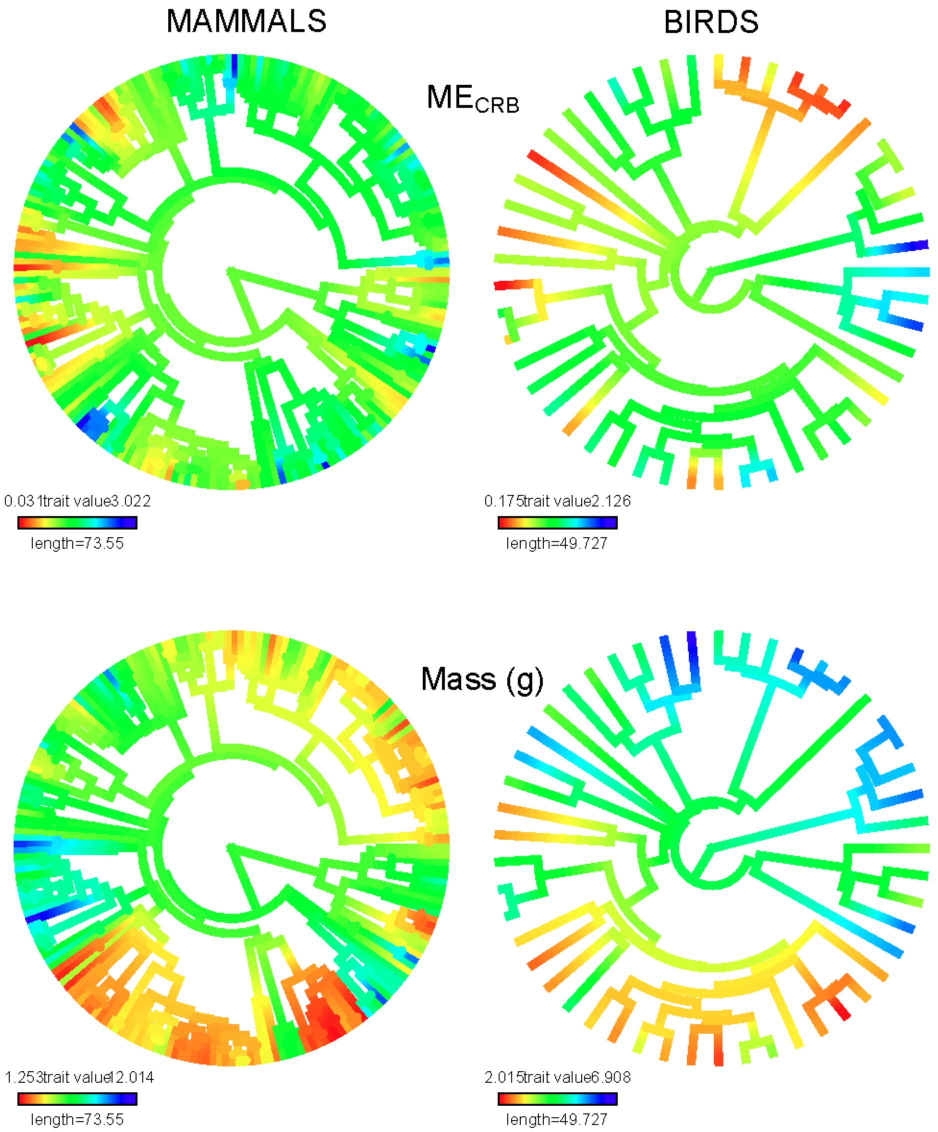

Figure S3. Phylogenetic conservatism of mammals (left) and birds (right) in metabolic expansibility at the cold range boundary (ME_{CRB}, top) and mass (g, bottom). Colors toward blue represent higher values (using a log scale).

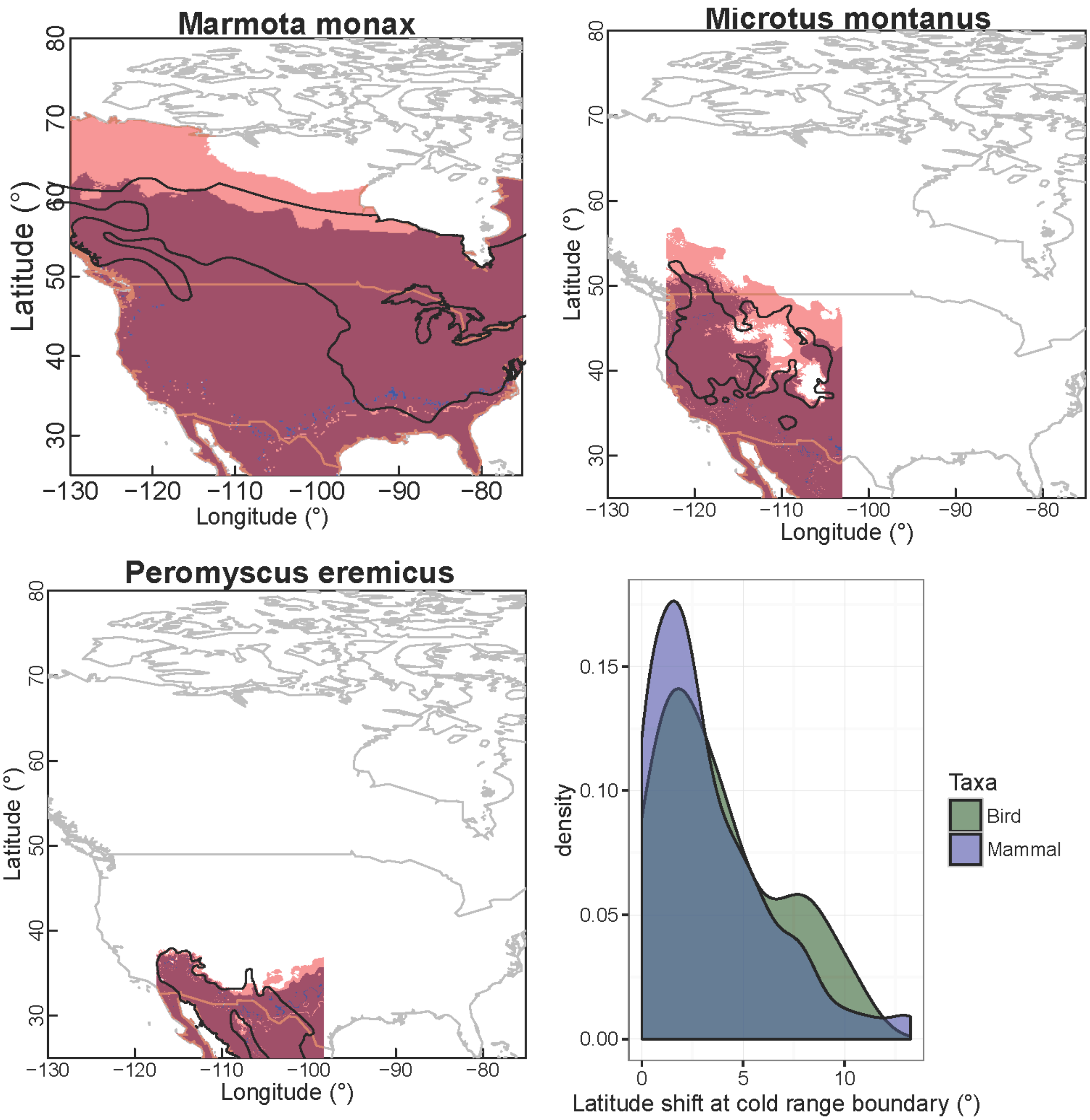

Figure S4. We depict observed cold range boundaries (CRB, black polygons: IUCN range maps) and those projected based on metabolic constraints for exemplar North American rodents in current (blue: 1950-2000) and predicted future (pink: 2061-2080 from CCSM4 model) climates (a – c). Purple shading indicates portions of the projected range occupancy that persists through climate warming. We note few areas of range contraction (blue) since we are only predicting CRBs (the depicted equatorward extent is not meaningful). We restrict our CRB projections to the observed longtitudinal extent. The species differ in the extent of their current distribution and the projected CRB shift resulting from climate change (a, *Marmota monax*, groundhog; b, *Microtus montanus*, montane vole; and c, *Peromyscus eremicus*, cactus mouse). Projections based on metabolic constraints indicate that the majority of mammals (purple) and birds (green) will shift their CRB modestly poleward through climate changes (d). However, numerous species are projected to shift their CRB poleward by 10° latitude and some species are projected to shift by as much as 30°.

Figure S5. We depict observed mammal cold range boundaries (CRB, black polygons: IUCN range maps) and those projected based on metabolic constraints in current (blue: 1950-2000) and predicted future (red: 2061-2080 from HadGEM2-AO model) climates (a – c). Purple shading indicates portions of the projected range occupancy that persists through climate warming. We note few areas of range contraction (blue) since we are only predicting CRBs (the depicted equatorward extent is not meaningful).

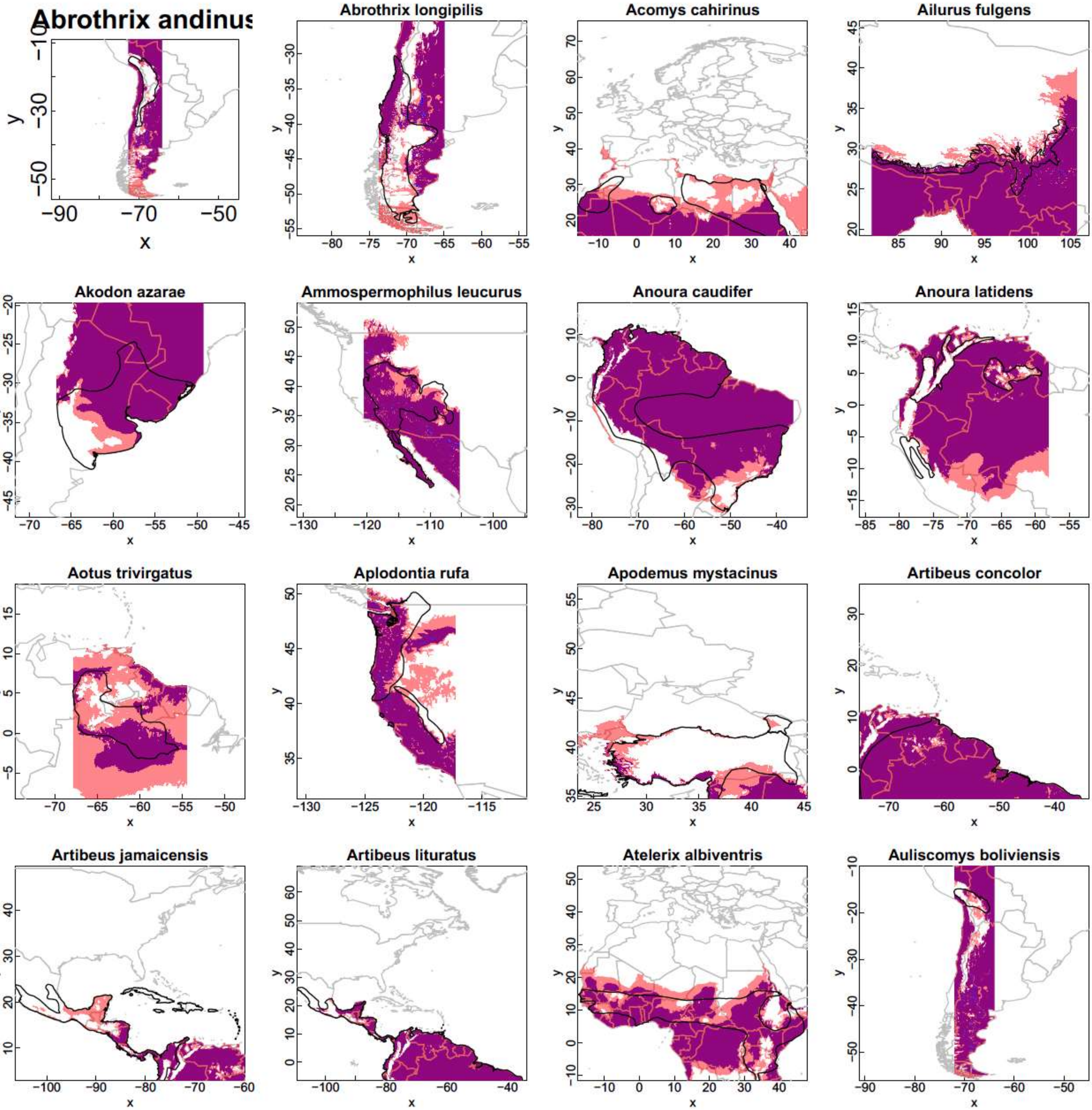

Abrothrix andinus
−10
−30
−50
y
−90
−70
−50
x
Abrothrix longipilis
−30
−35
−40
−45
−50
−55
y
−80
−75
−70
−65
−60
−55
x
Acomys cahirinus
70
60
50
40
30
20
y
−10
0
10
20
30
40
x
Ailurus fulgens
45
40
35
30
25
20
y
85
90
95
100
105
x
Akodon azarae
−20
−25
−30
−35
−40
−45
y
−70
−65
−60
−55
−50
−45
x
Ammospermophilus leucurus
50
45
40
35
30
25
20
y
−130
−120
−110
−100
x
Anoura caudifer
10
0
−10
−20
−30
y
−80
−70
−60
−50
−40
x
Anoura latidens
15
10
5
0
−5
−10
−15
y
−85
−80
−75
−70
−65
−60
−55
x
Aotus trivirgatus
15
10
5
0
−5
y
−70
−65
−60
−55
−50
x
Aplodontia rufa
50
45
40
35
y
−130
−125
−120
−115
x
Apodemus mystacinus
55
50
45
40
35
y
25
30
35
40
45
x
Artibeus concolor
30
20
10
0
y
−70
−60
−50
−40
x
Artibeus jamaicensis
40
30
20
10
y
−100
−90
−80
−70
−60
x
Artibeus lituratus
60
50
40
30
20
10
0
y
−100
−80
−60
−40
x
Atelerix albiventris
50
40
30
20
10
0
−10
y
−10
0
10
20
30
40
x
Auliscomys boliviensis
−10
−20
−30
−40
−50
y
−90
−80
−70
−60
−50
x

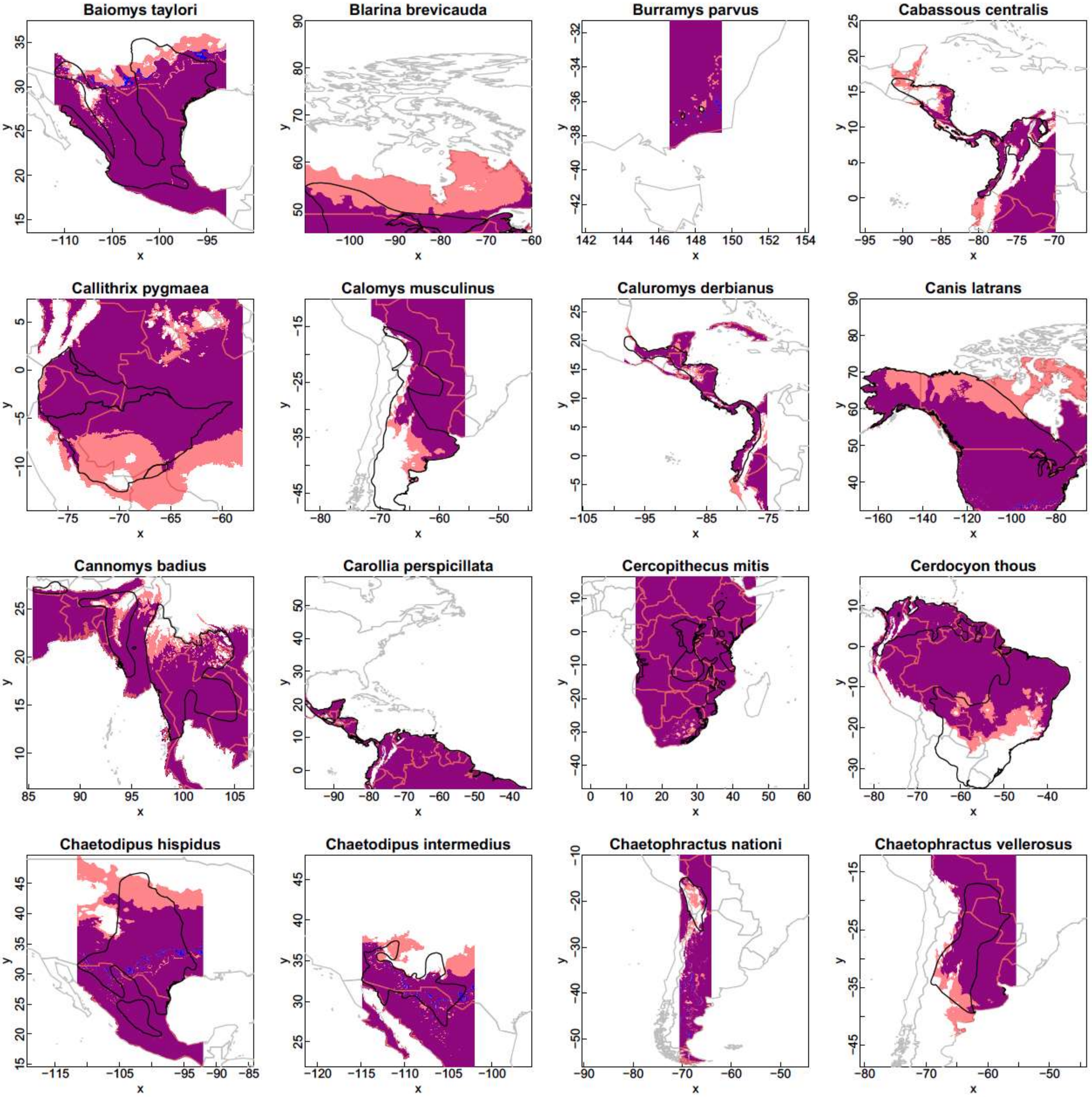
Baiomys taylori
Blarina brevicauda
Burramys parvus
Cabassous centralis
Callithrix pygmaea
Calomys musculinus
Caluromys derbianus
Canis latrans
Cannomys badius
Carollia perspicillata
Cercopithecus mitis
Cerdocyon thous
Chaetodipus hispidus
Chaetodipus intermedius
Chaetophractus nationi
Chaetophractus vellerosus

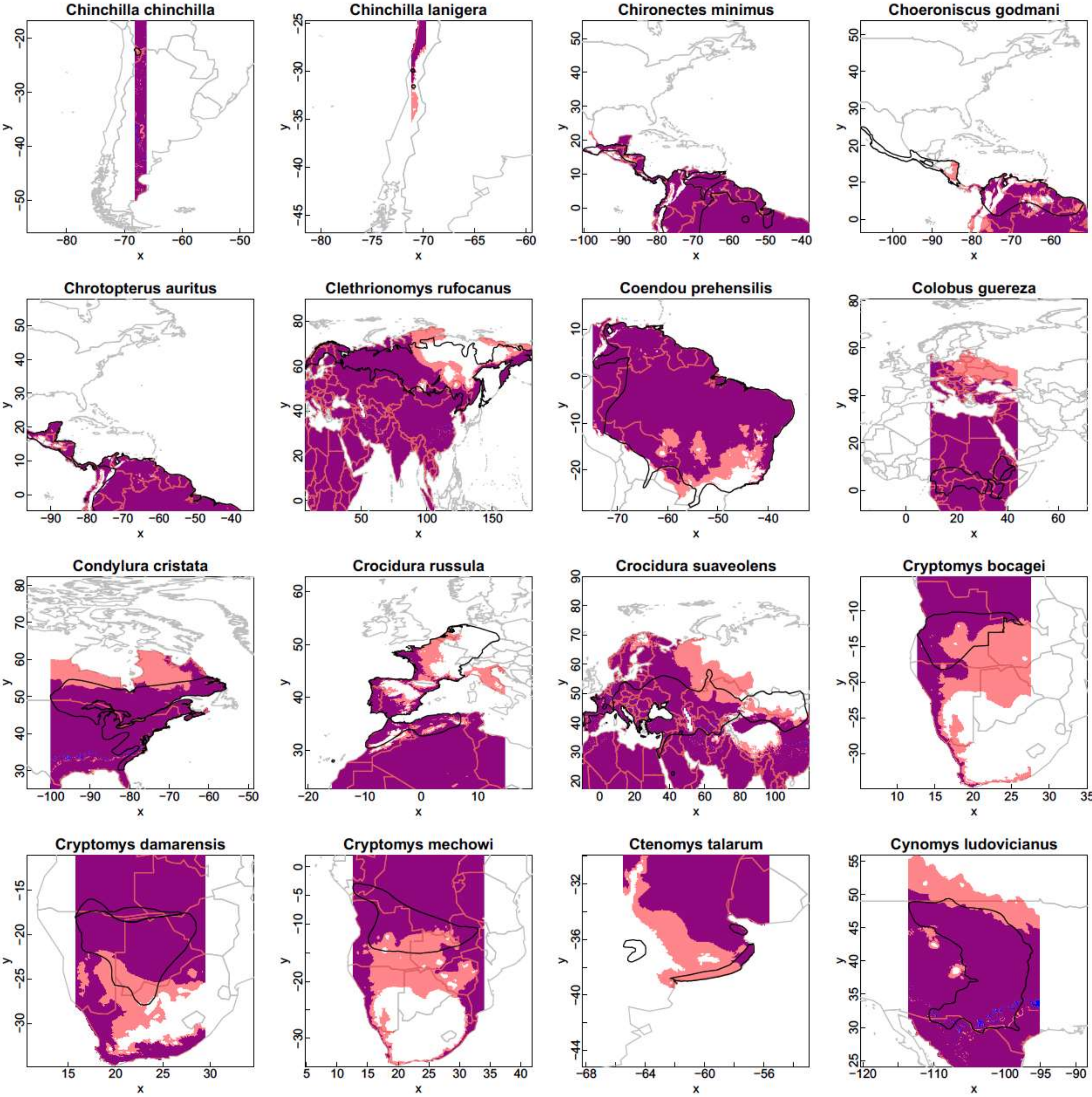
Chinchilla chinchilla
-20 -30 -40 -50
y
-80 -70 -60 -50
x
Chinchilla lanigera
-25 -30 -35 -40 -45
y
-80 -75 -70 -65 -60
x
Chironectes minimus
50 40 30 20 10 0
y
-100 -90 -80 -70 -60 -50 -40
x
Choeroniscus godmani
50 40 30 20 10 0
y
-100 -90 -80 -70 -60
x
Chrotopterus auritus
50 40 30 20 10 0
y
-90 -80 -70 -60 -50 -40
x
Clethrionomys rufocanus
80 60 40 20 0
y
50 100 150
x
Coendou prehensilis
10 0 -10 -20
y
-70 -60 -50 -40
x
Colobus guereza
80 60 40 20 0
y
0 20 40 60
x
Condylura cristata
80 70 60 50 40 30
y
-100 -90 -80 -70 -60 -50
x
Crocidura russula
60 50 40 30
y
-20 -10 0 10
x
Crocidura suaveolens
90 80 70 60 50 40 30 20
y
0 20 40 60 80 100
x
Cryptomys bocagei
-10 -15 -20 -25 -30
y
10 15 20 25 30 35
x
Cryptomys damarensis
-15 -20 -25 -30
y
15 20 25 30
x
Cryptomys mechowi
0 -5 -10 -20 -30
y
5 10 15 20 25 30 35 40
x
Ctenomys talarum
-32 -36 -40 -44
y
-68 -64 -60 -56
x
Cynomys ludovicianus
55 50 45 40 35 30 25
y
-120 -110 -100 -95 -90
x

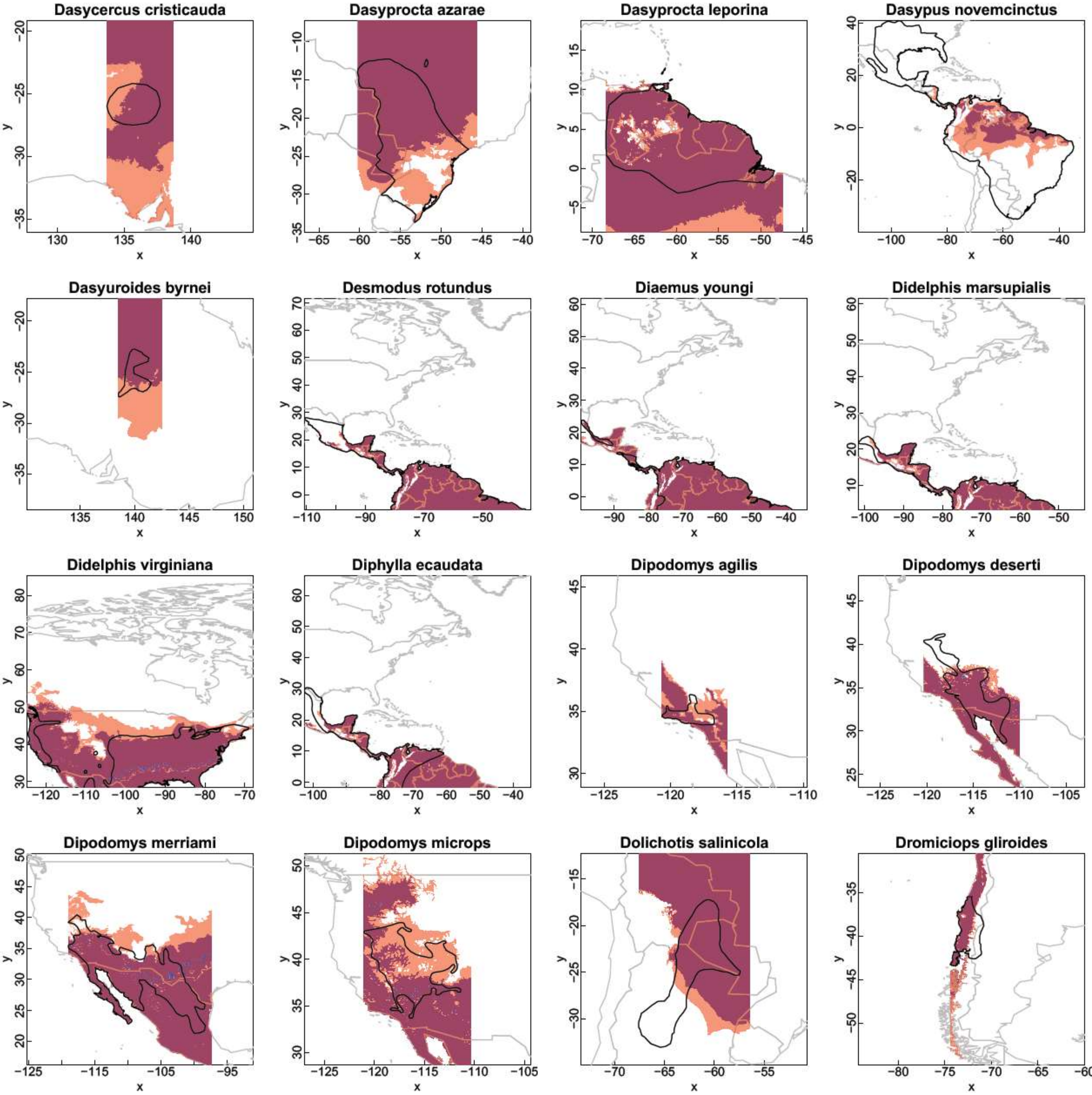
Dasycercus cristicauda
Dasyprocta azarae
Dasyprocta leporina
Dasypus novemcinctus
Dasyuroides byrnei
Desmodus rotundus
Diaemus youngi
Didelphis marsupialis
Didelphis virginiana
Diphylla ecaudata
Dipodomys agilis
Dipodomys deserti
Dipodomys merriami
Dipodomys microps
Dolichotis salinicola
Dromiciops gliroides

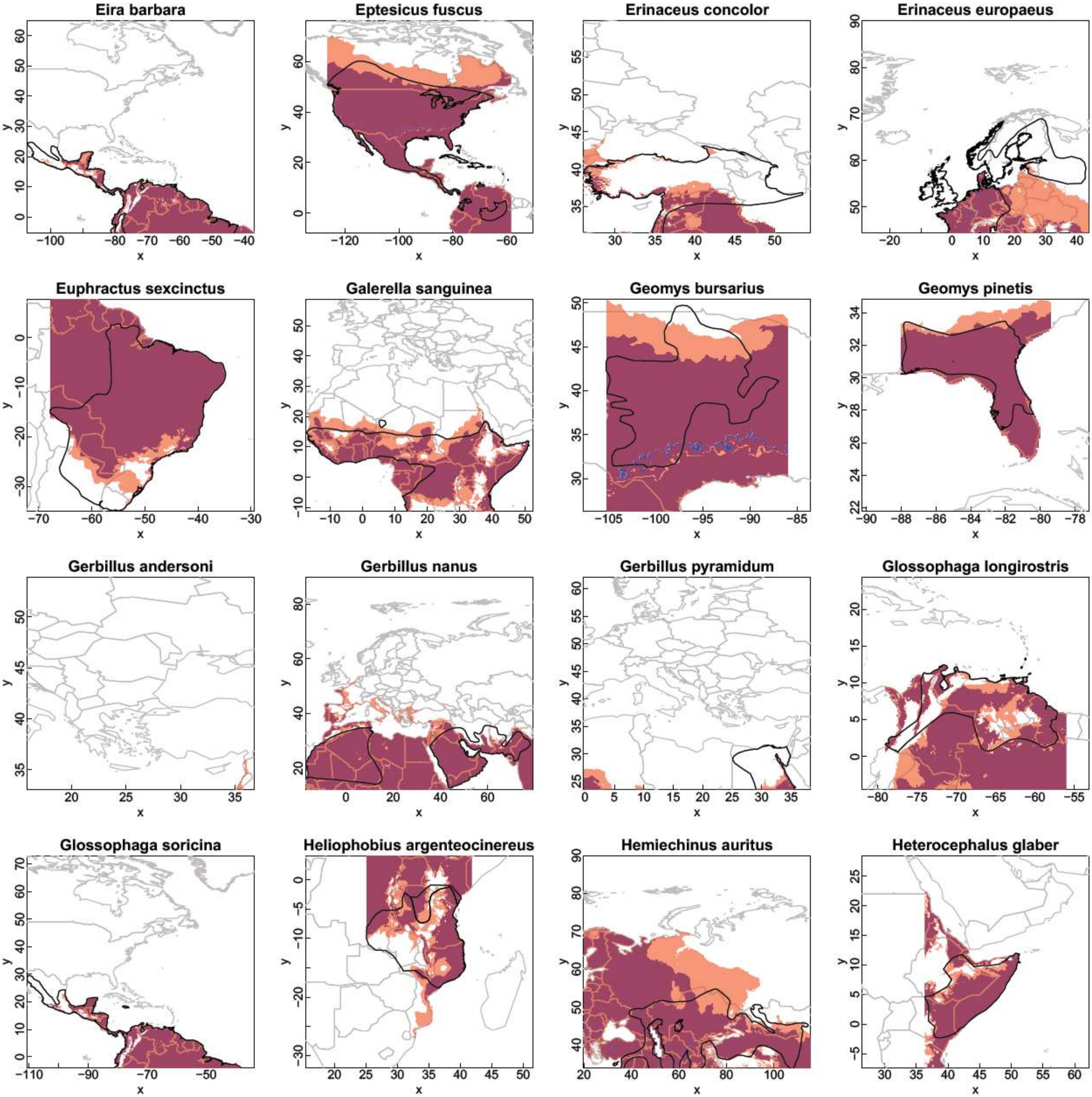

Eira barbara
Eptesicus fuscus
Erinaceus concolor
Erinaceus europaeus
Euphractus sexcinctus
Galerella sanguinea
Geomys bursarius
Geomys pinetis
Gerbillus andersoni
Gerbillus nanus
Gerbillus pyramidum
Glossophaga longirostris
Glossophaga soricina
Heliophobius argenteocinereus
Hemiechinus auritus
Heterocephalus glaber
x
y

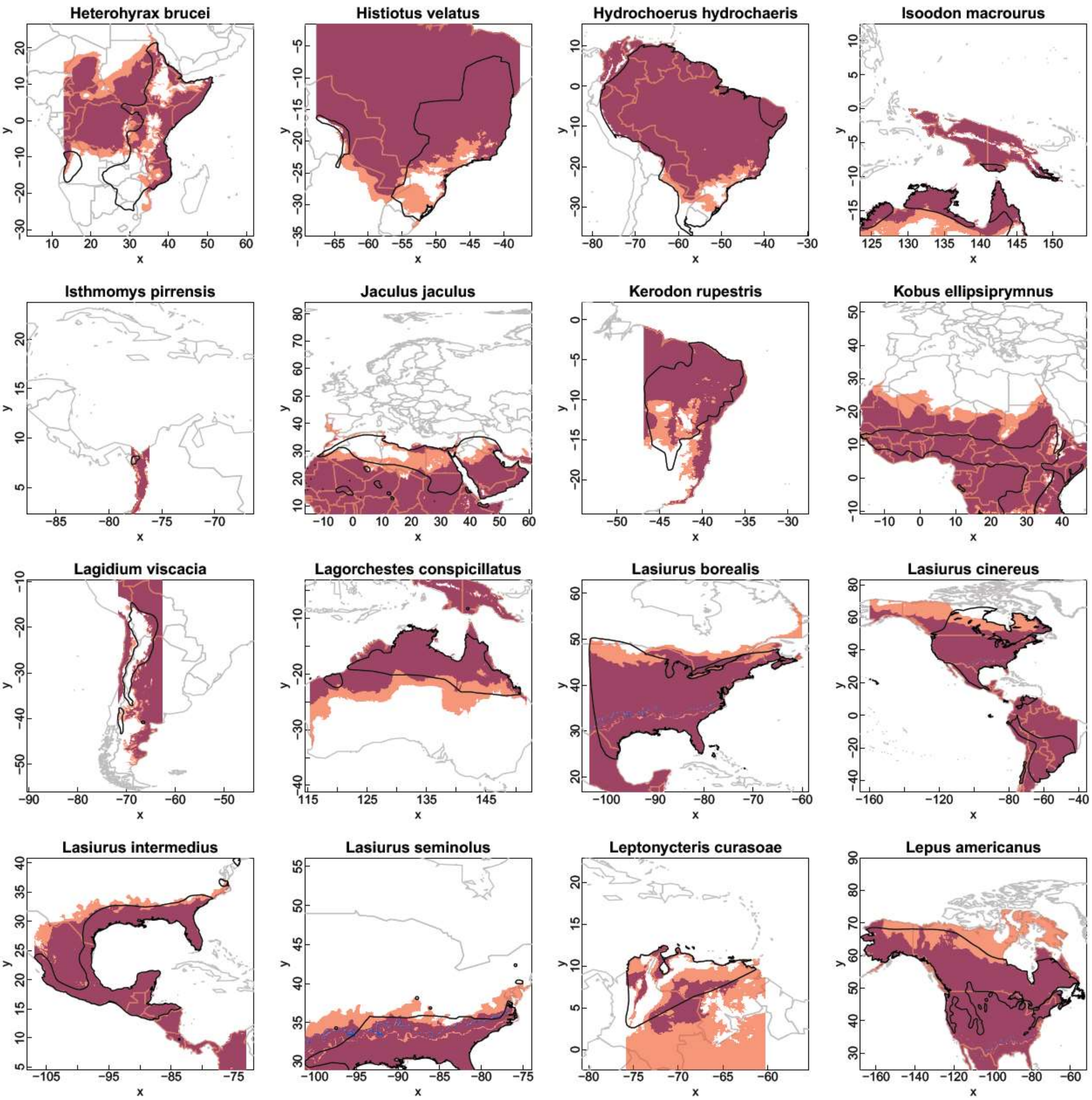
Heterohyrax brucei
Histiotus velatus
Hydrochoerus hydrochaeris
Isoodon macrourus
Isthmomys pirrensis
Jaculus jaculus
Kerodon rupestris
Kobus ellipsiprymnus
Lagidium viscacia
Lagorchestes conspicillatus
Lasiurus borealis
Lasiurus cinereus
Lasiurus intermedius
Lasiurus seminolus
Leptonycteris curasoae
Lepus americanus
x
y

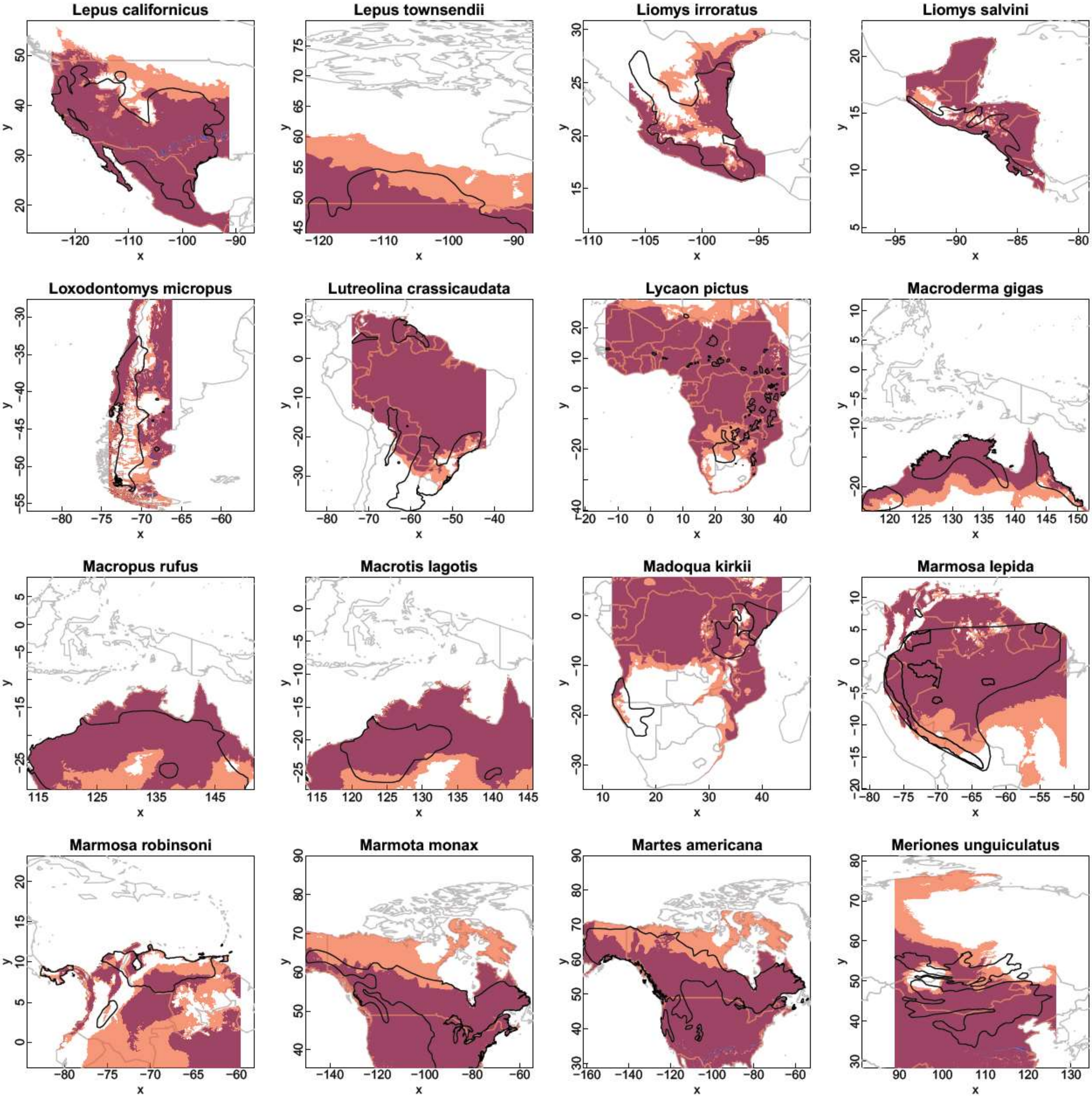
Lepus californicus
Lepus townsendii
Liomys irroratus
Liomys salvini
Loxodontomys micropus
Lutreolina crassicaudata
Lycaon pictus
Macroderma gigas
Macropus rufus
Macrotis lagotis
Madoqua kirkii
Marmosa lepida
Marmosa robinsoni
Marmota monax
Martes americana
Meriones unguiculatus
x
y

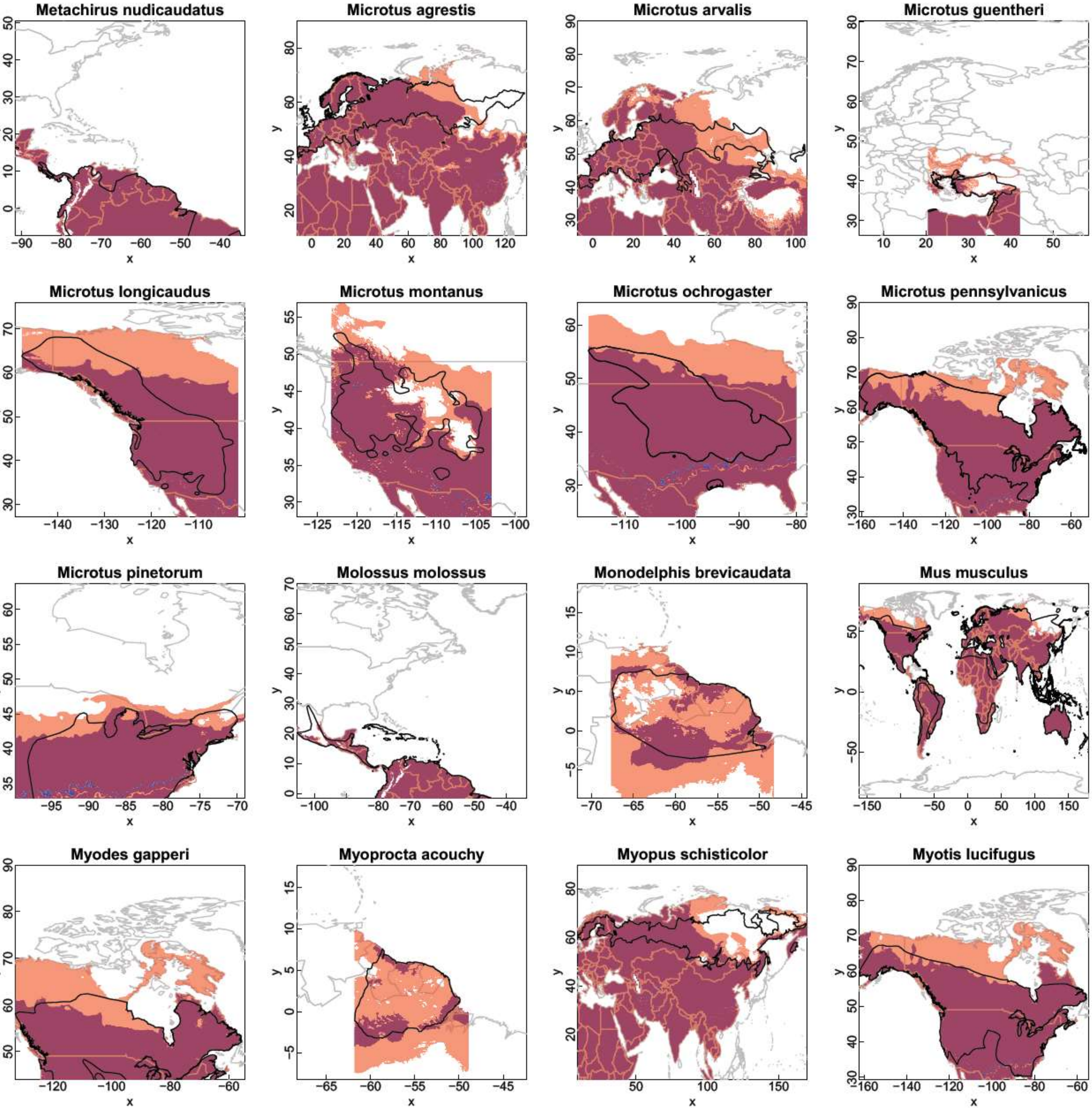
Metachirus nudicaudatus
Microtus agrestis
Microtus arvalis
Microtus guentheri
Microtus longicaudus
Microtus montanus
Microtus ochrogaster
Microtus pennsylvanicus
Microtus pinetorum
Molossus molossus
Monodelphis brevicaudata
Mus musculus
Myodes gapperi
Myoprocta acouchy
Myopus schisticolor
Myotis lucifugus

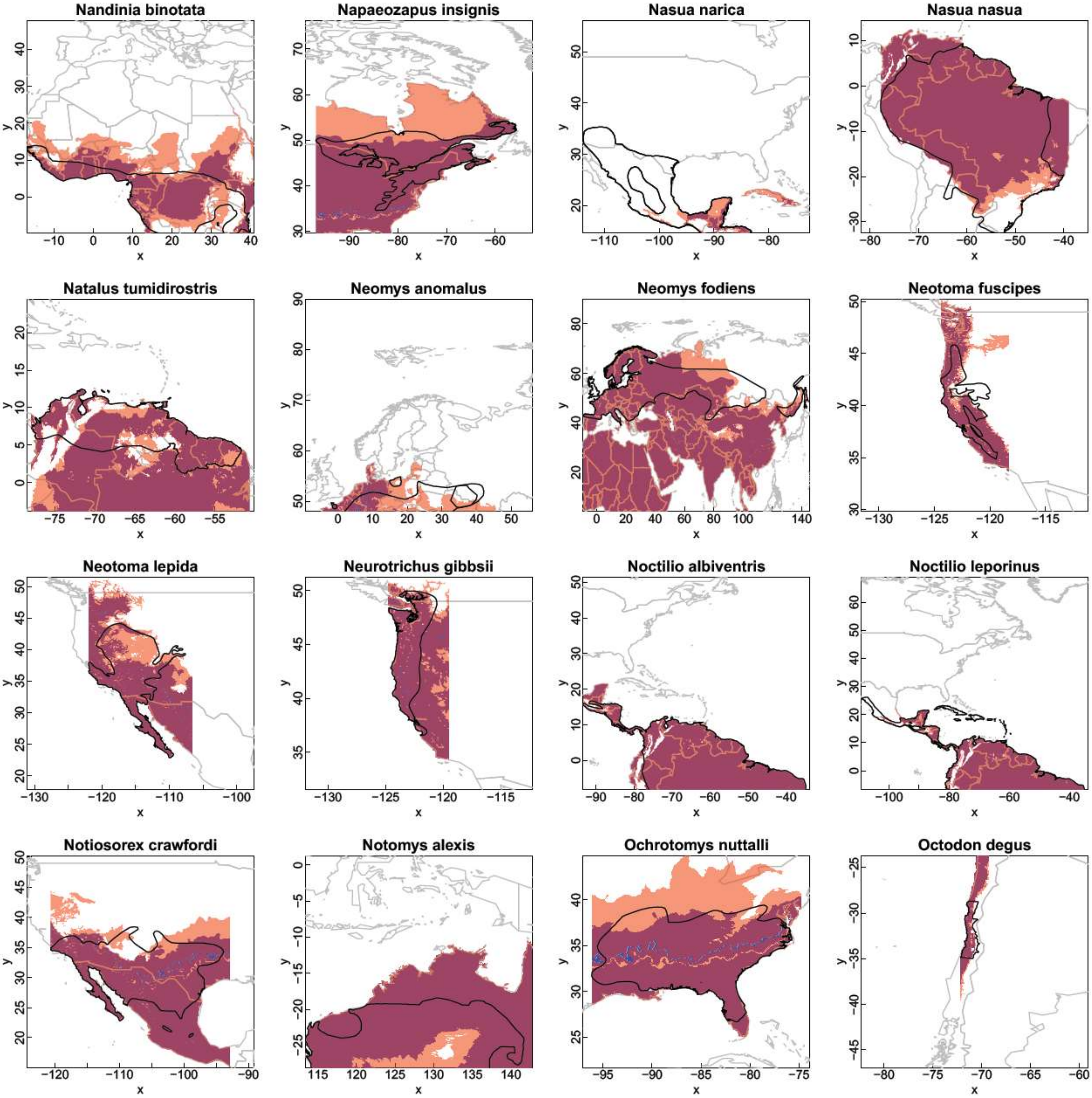
Nandinia binotata
Napaeozapus insignis
Nasua narica
Nasua nasua
Natalus tumidirostris
Neomys anomalus
Neomys fodiens
Neotoma fuscipes
Neotoma lepida
Neurotrichus gibbsii
Noctilio albiventris
Noctilio leporinus
Notiosorex crawfordi
Notomys alexis
Ochrotomys nuttalli
Octodon degus

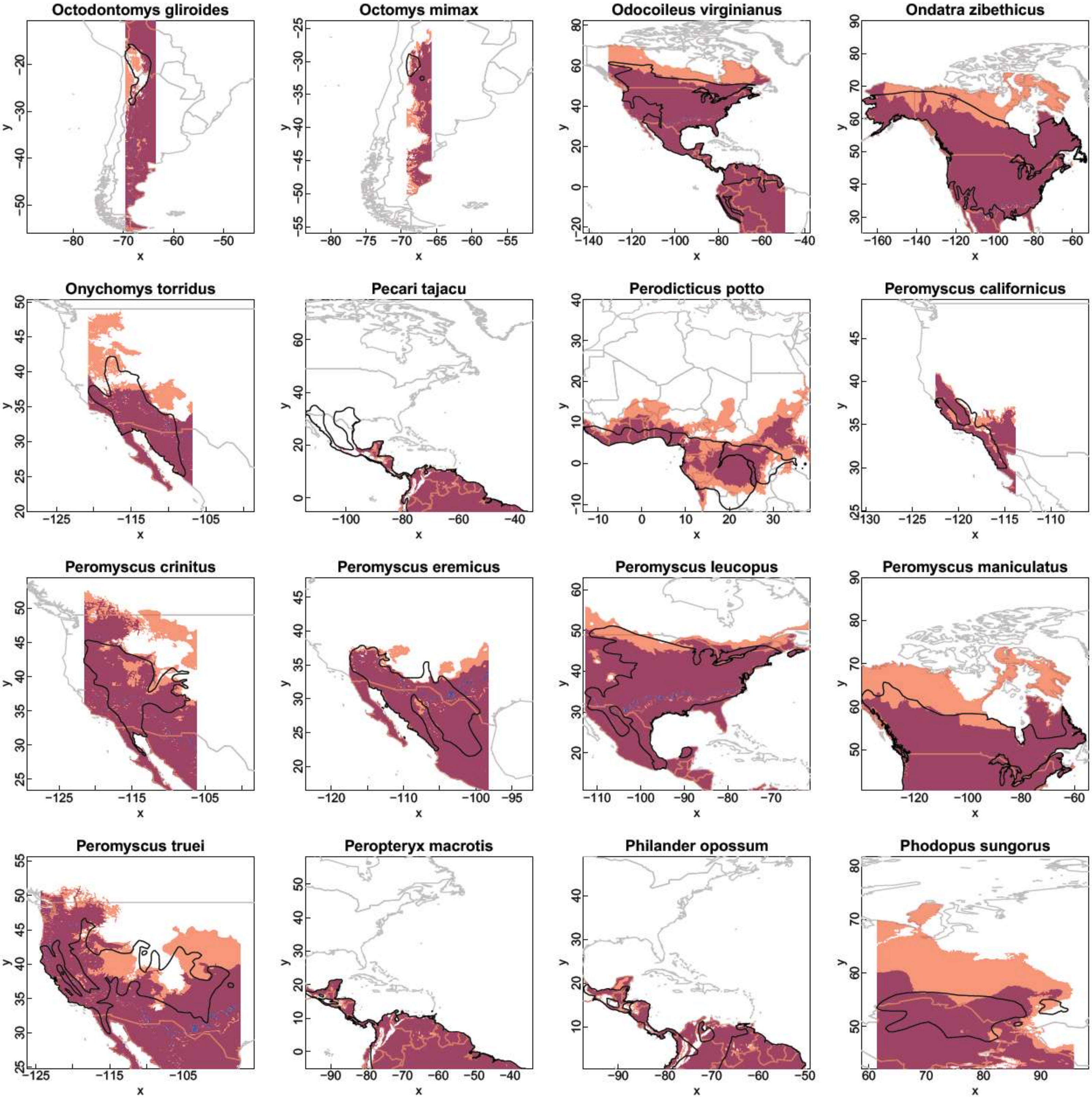
Octodontomys gliroides
Octomys mimax
Odocoileus virginianus
Ondatra zibethicus
Onychomys torridus
Pecari tajacu
Perodicticus potto
Peromyscus californicus
Peromyscus crinitus
Peromyscus eremicus
Peromyscus leucopus
Peromyscus maniculatus
Peromyscus truei
Peropteryx macrotis
Philander opossum
Phodopus sungorus
x
y

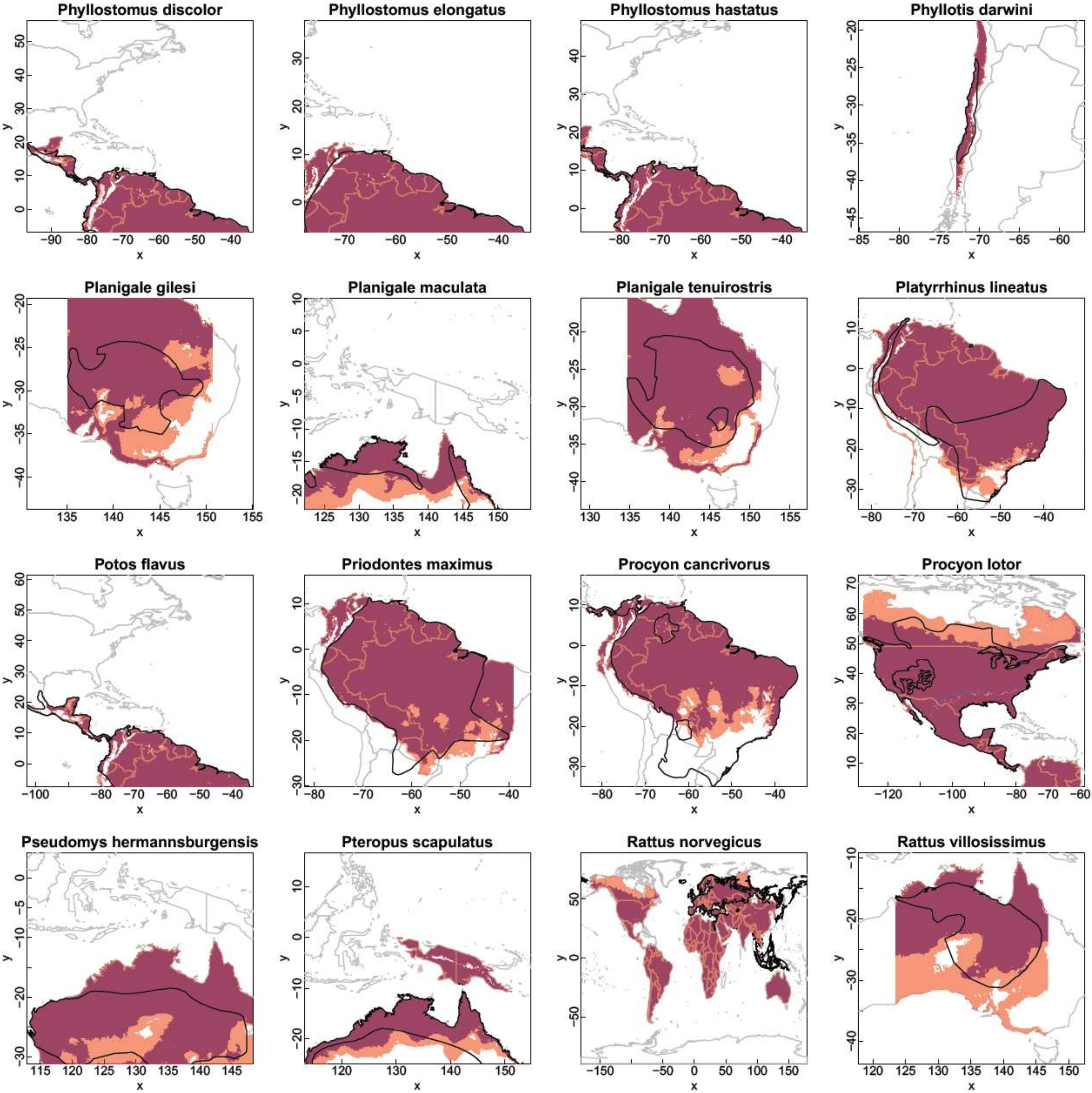
Phyllostomus discolor
Phyllostomus elongatus
Phyllostomus hastatus
Phyllotis darwini
Planigale gilesi
Planigale maculata
Planigale tenuirostris
Platyrrhinus lineatus
Potos flavus
Priodontes maximus
Procyon cancrivorus
Procyon lotor
Pseudomys hermannsburgensis
Pteropus scapulatus
Rattus norvegicus
Rattus villosissimus
x
y

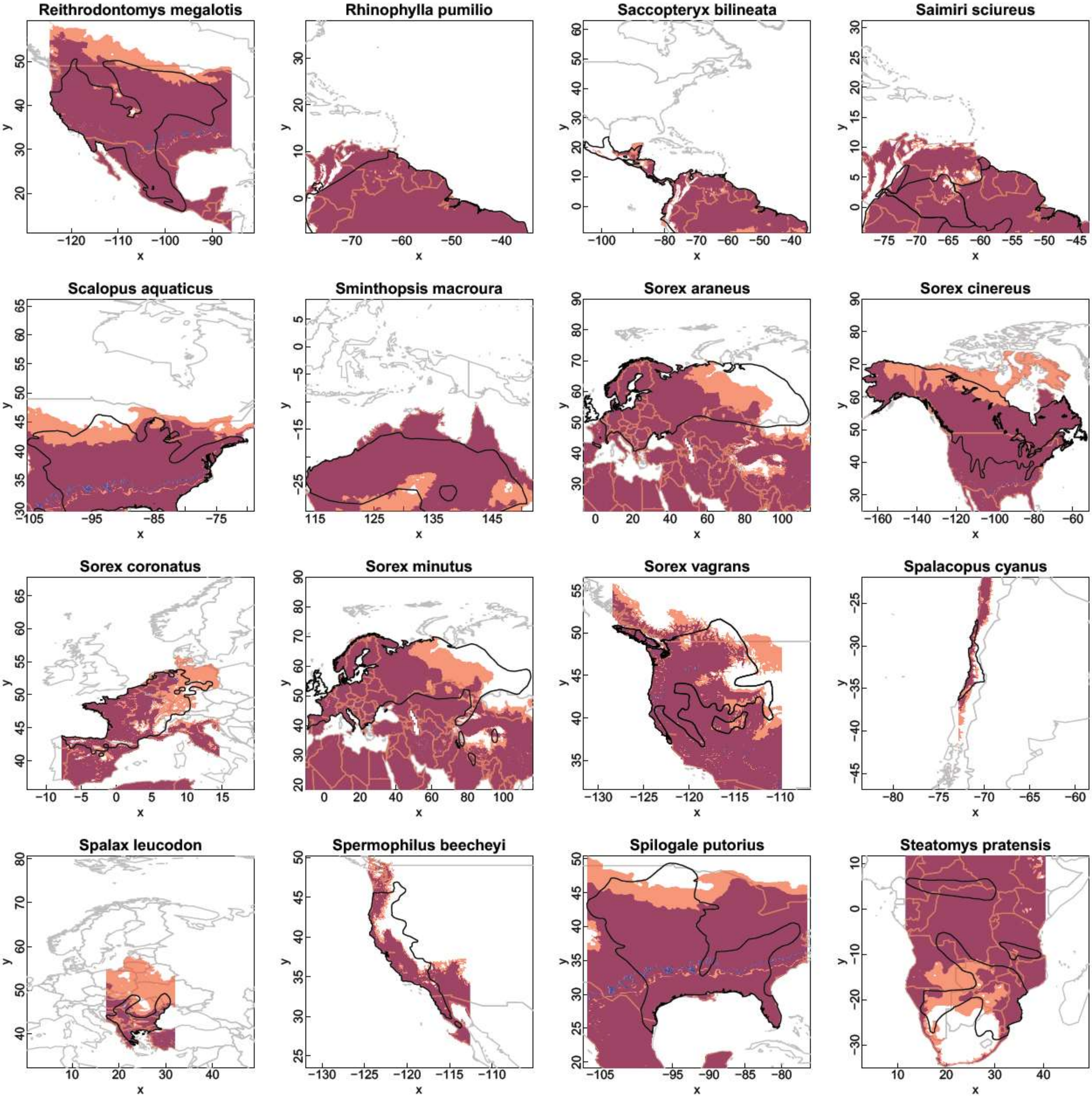

Reithrodontomys megalotis
Rhinophylla pumilio
Saccopteryx bilineata
Saimiri sciureus
Scalopus aquaticus
Sminthopsis macroura
Sorex araneus
Sorex cinereus
Sorex coronatus
Sorex minutus
Sorex vagrans
Spalacopus cyanus
Spalax leucodon
Spermophilus beecheyi
Spilogale putorius
Steatomys pratensis
x
y

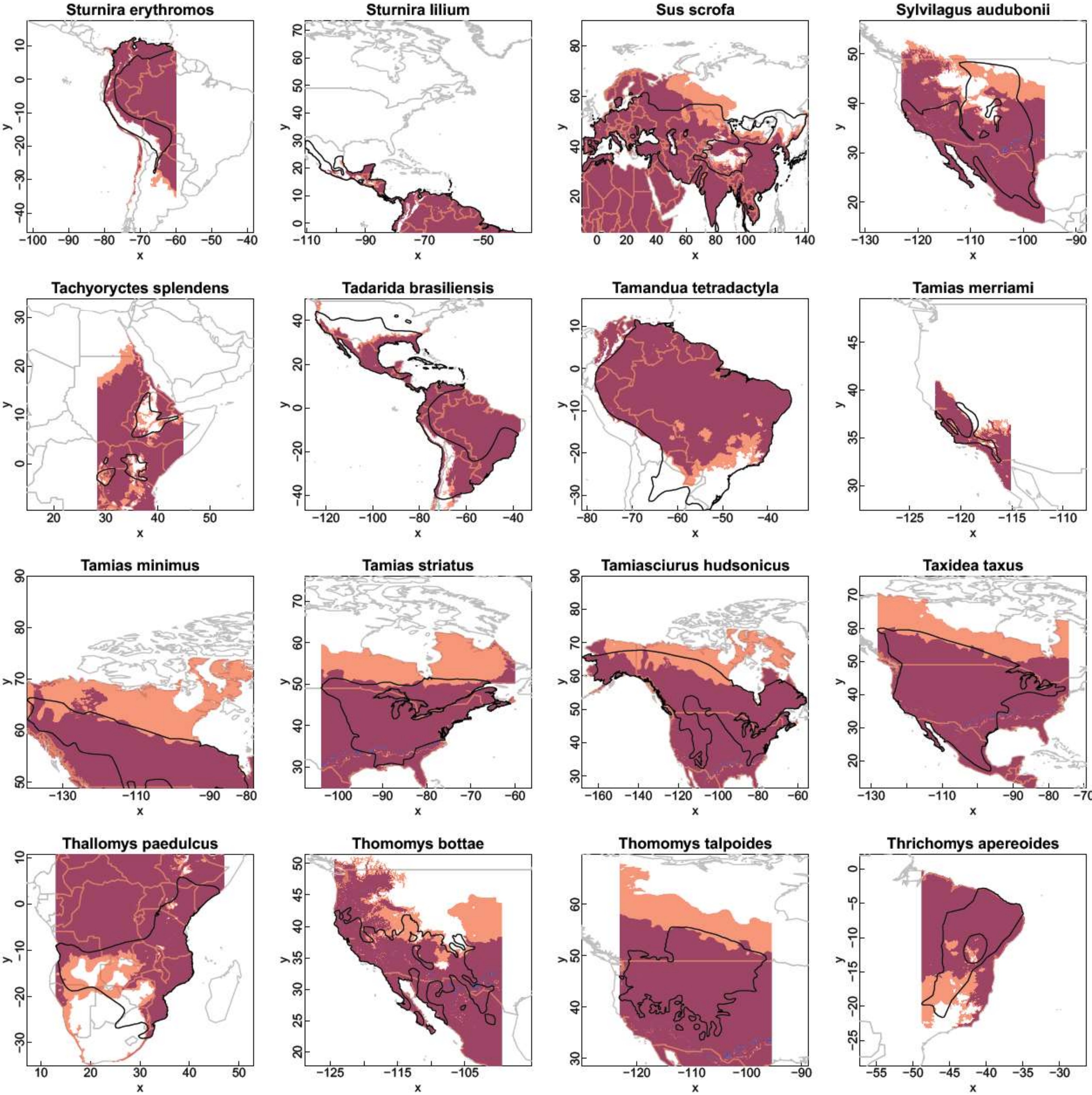
Sturnira erythromos
Sturnira lilium
Sus scrofa
Sylvilagus audubonii
Tachyoryctes splendens
Tadarida brasiliensis
Tamandua tetradactyla
Tamias merriami
Tamias minimus
Tamias striatus
Tamiasciurus hudsonicus
Taxidea taxus
Thallomys paedulcus
Thomomys bottae
Thomomys talpoides
Thrichomys apereoides
x
y

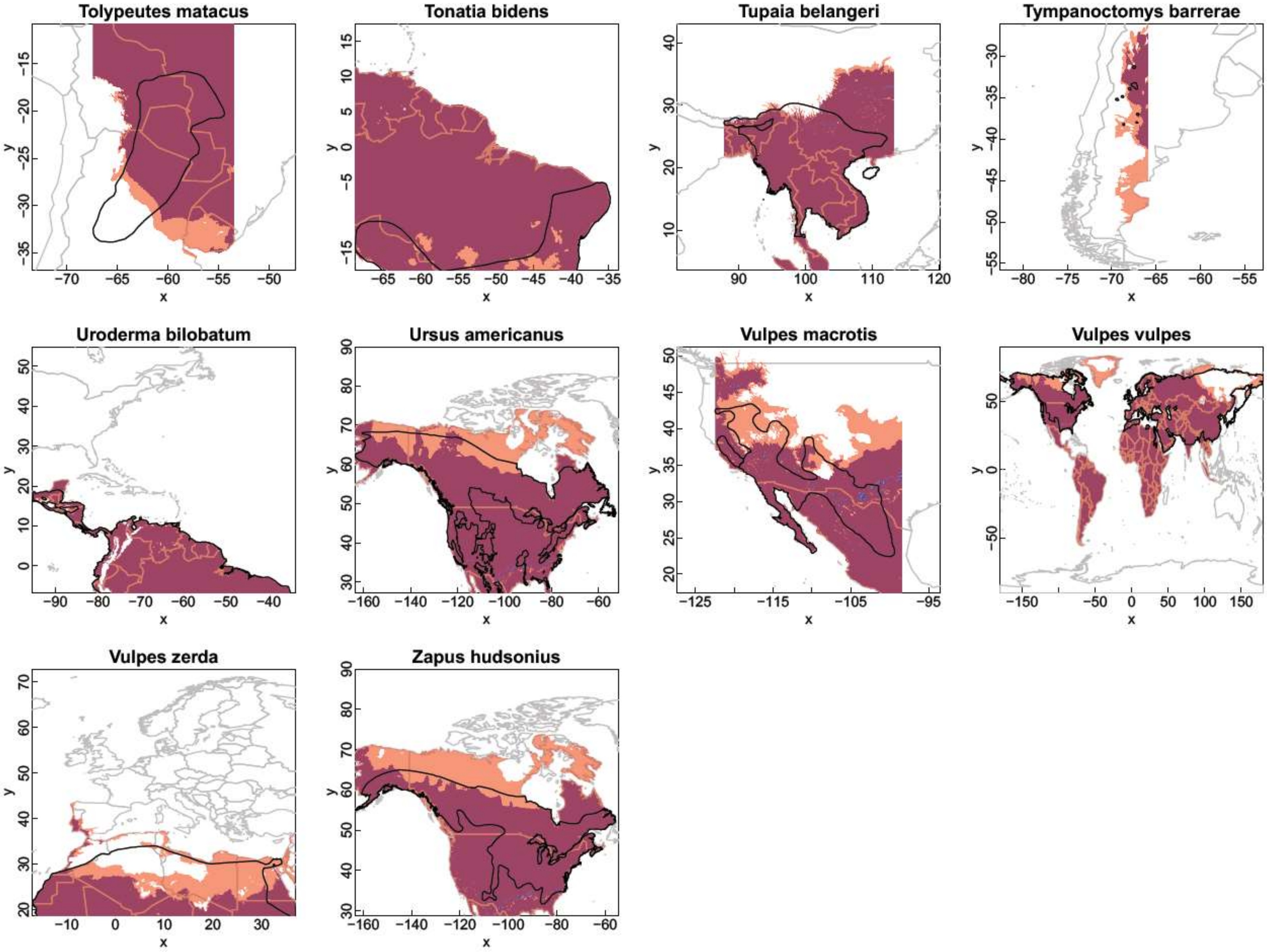
Tolypeutes matacus
−15 −20 −25 −30 −35
−70 −65 −60 −55 −50
Tonatia bidens
15 10 5 0 −5 −15
−65 −60 −55 −50 −45 −40 −35
Tupaia belangeri
40 30 20 10
90 100 110 120
Tympanoctomys barrerae
−30 −35 −40 −45 −50 −55
−80 −75 −70 −65 −60 −55
Uroderma bilobatum
50 40 30 20 10 0
−90 −80 −70 −60 −50 −40
Ursus americanus
90 80 70 60 50 40 30
−160 −140 −120 −100 −80 −60
Vulpes macrotis
50 45 40 35 30 25 20
−125 −115 −105 −95
Vulpes vulpes
50 0 −50
−150 −50 0 50 100 150
Vulpes zerda
70 60 50 40 30 20
−10 0 10 20 30
Zapus hudsonius
90 80 70 60 50 40 30
−160 −140 −120 −100 −80 −60
x
y

Figure S6. We depict observed mammal cold range boundaries (CRB, black polygons: IUCN range maps) and those projected based on metabolic constraints in current (blue: 1950-2000) and predicted future (red: 2061-2080 from CCSM4 model) climates (a – c). Purple shading indicates portions of the projected range occupancy that persists through climate warming. We note few areas of range contraction (blue) since we are only predicting CRBs (the depicted equatorward extent is not meaningful).

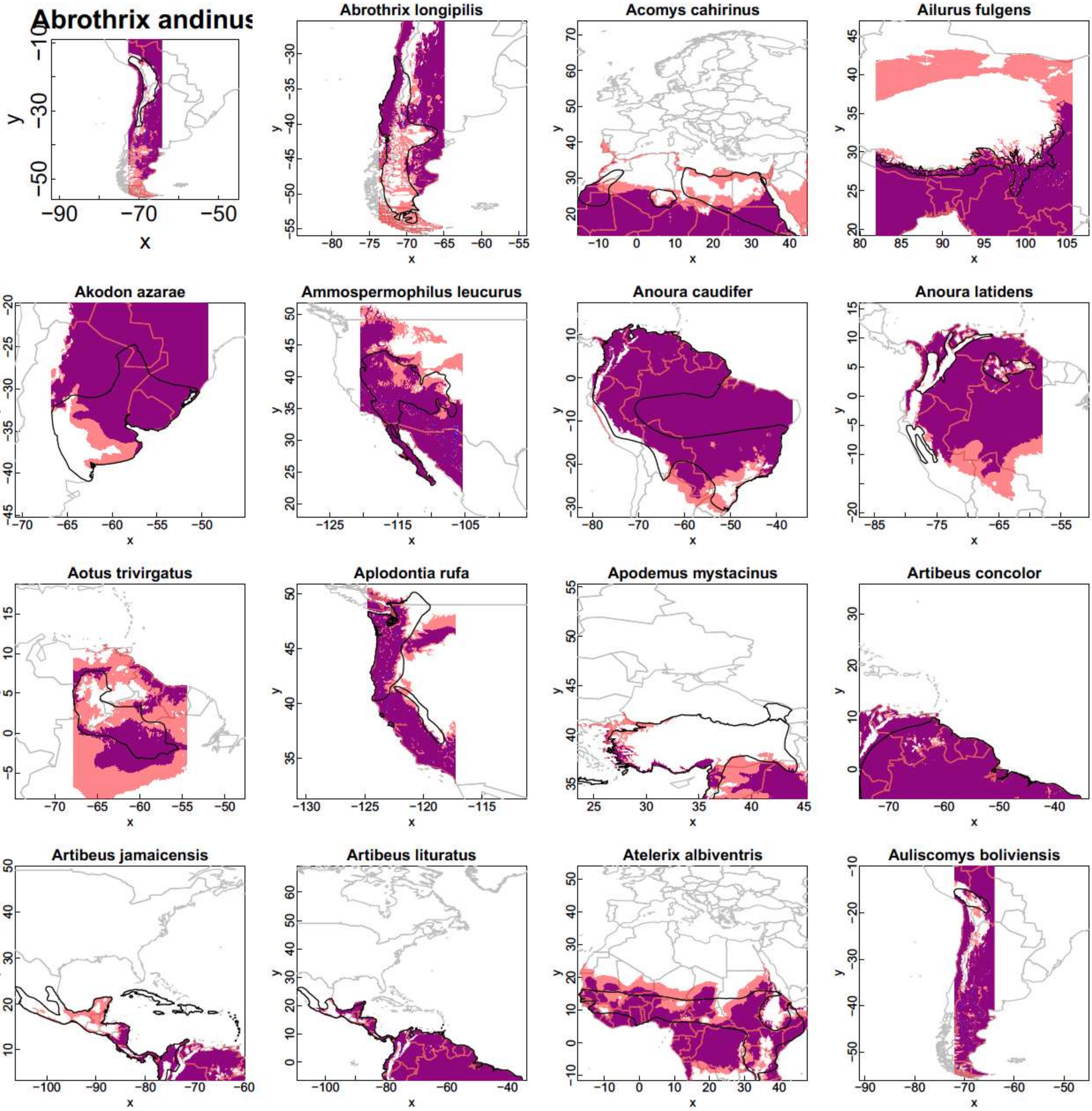
Abrothrix andinus
Abrothrix longipilis
Acomys cahirinus
Ailurus fulgens
Akodon azarae
Ammospermophilus leucurus
Anoura caudifer
Anoura latidens
Aotus trivirgatus
Aplodontia rufa
Apodemus mystacinus
Artibeus concolor
Artibeus jamaicensis
Artibeus lituratus
Atelerix albiventris
Auliscomys boliviensis
x
y

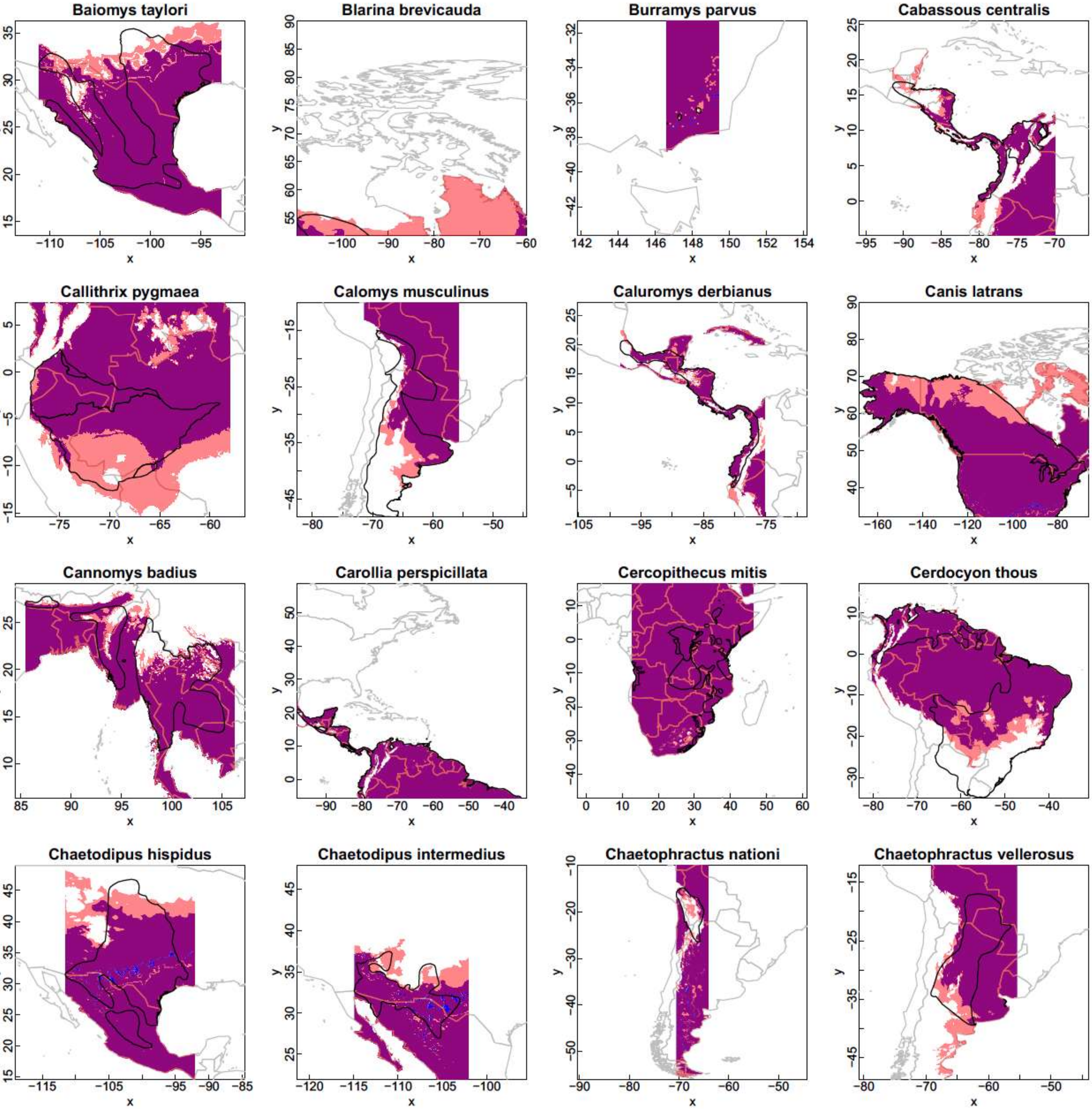
Baiomys taylori
Blarina brevicauda
Burramys parvus
Cabassous centralis
Callithrix pygmaea
Calomys musculinus
Caluromys derbianus
Canis latrans
Cannomys badius
Carollia perspicillata
Cercopithecus mitis
Cerdocyon thous
Chaetodipus hispidus
Chaetodipus intermedius
Chaetophractus nationi
Chaetophractus vellerosus

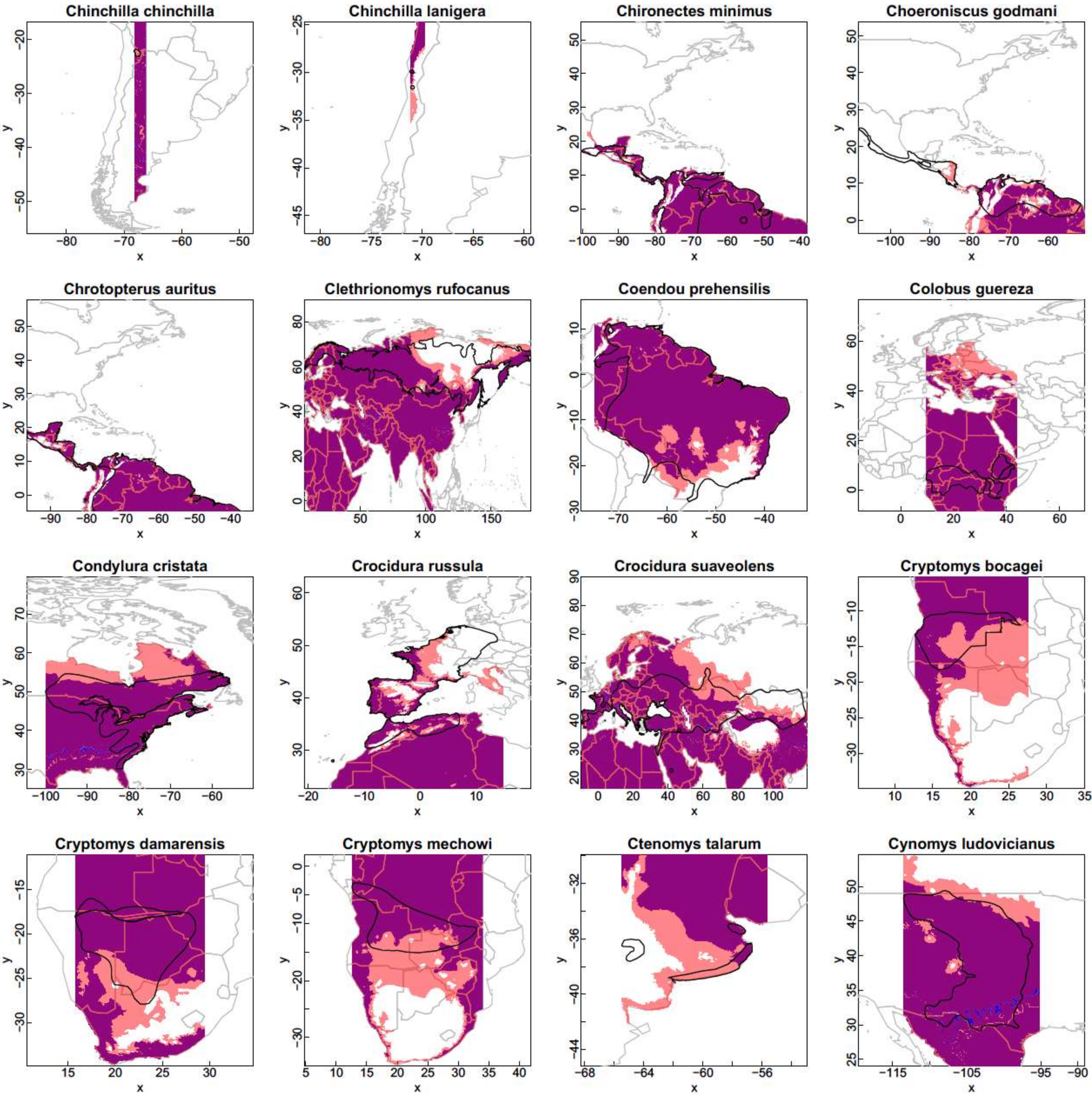
Chinchilla chinchilla
Chinchilla lanigera
Chironectes minimus
Choeroniscus godmani
Chrotopterus auritus
Clethrionomys rufocanus
Coendou prehensilis
Colobus guereza
Condylura cristata
Crocidura russula
Crocidura suaveolens
Cryptomys bocagei
Cryptomys damarensis
Cryptomys mechowi
Ctenomys talarum
Cynomys ludovicianus
x
y

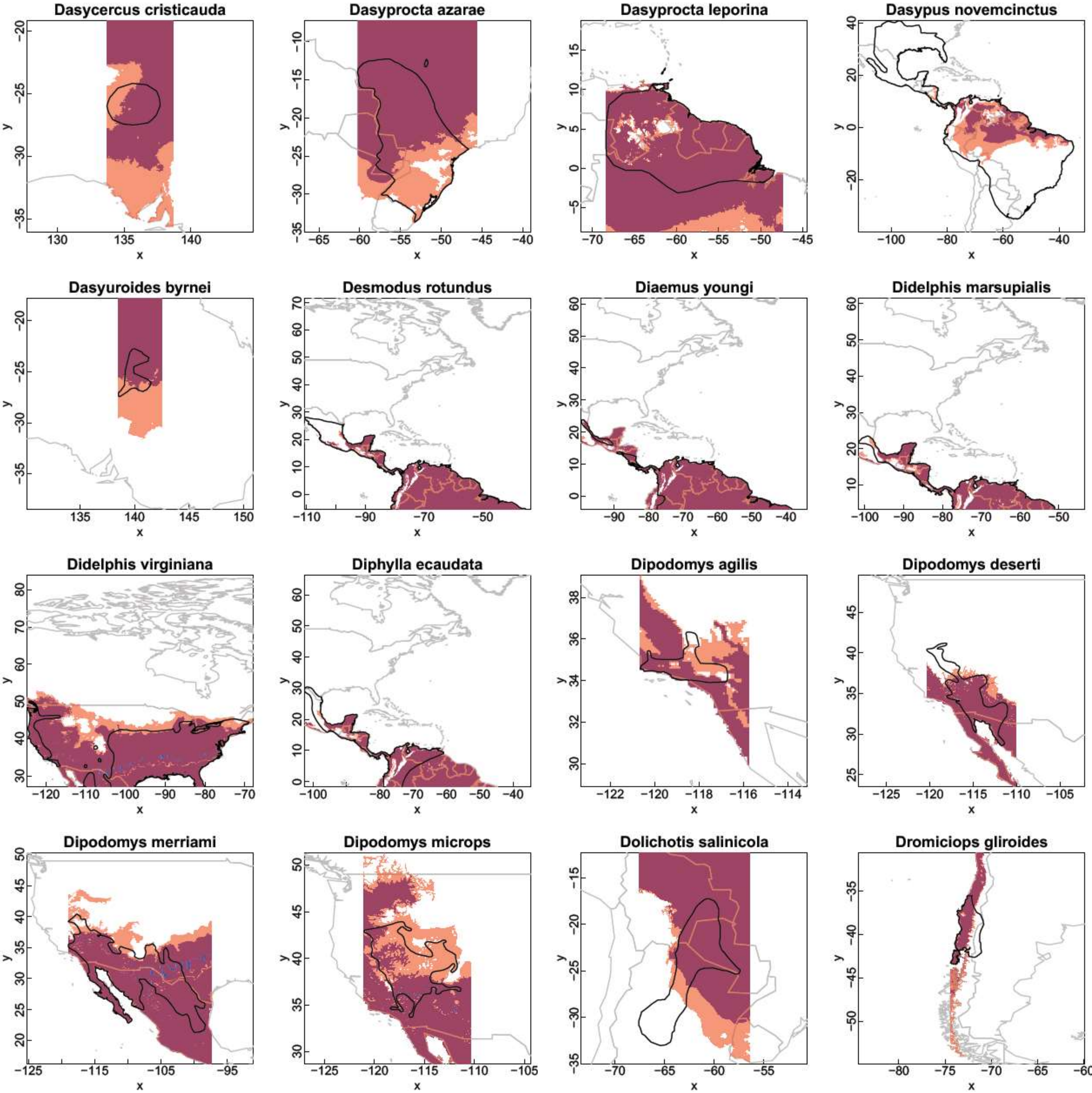
Dasycercus cristicauda
Dasyprocta azarae
Dasyprocta leporina
Dasypus novemcinctus
Dasyuroides byrnei
Desmodus rotundus
Diaemus youngi
Didelphis marsupialis
Didelphis virginiana
Diphylla ecaudata
Dipodomys agilis
Dipodomys deserti
Dipodomys merriami
Dipodomys microps
Dolichotis salinicola
Dromiciops gliroides
x
y

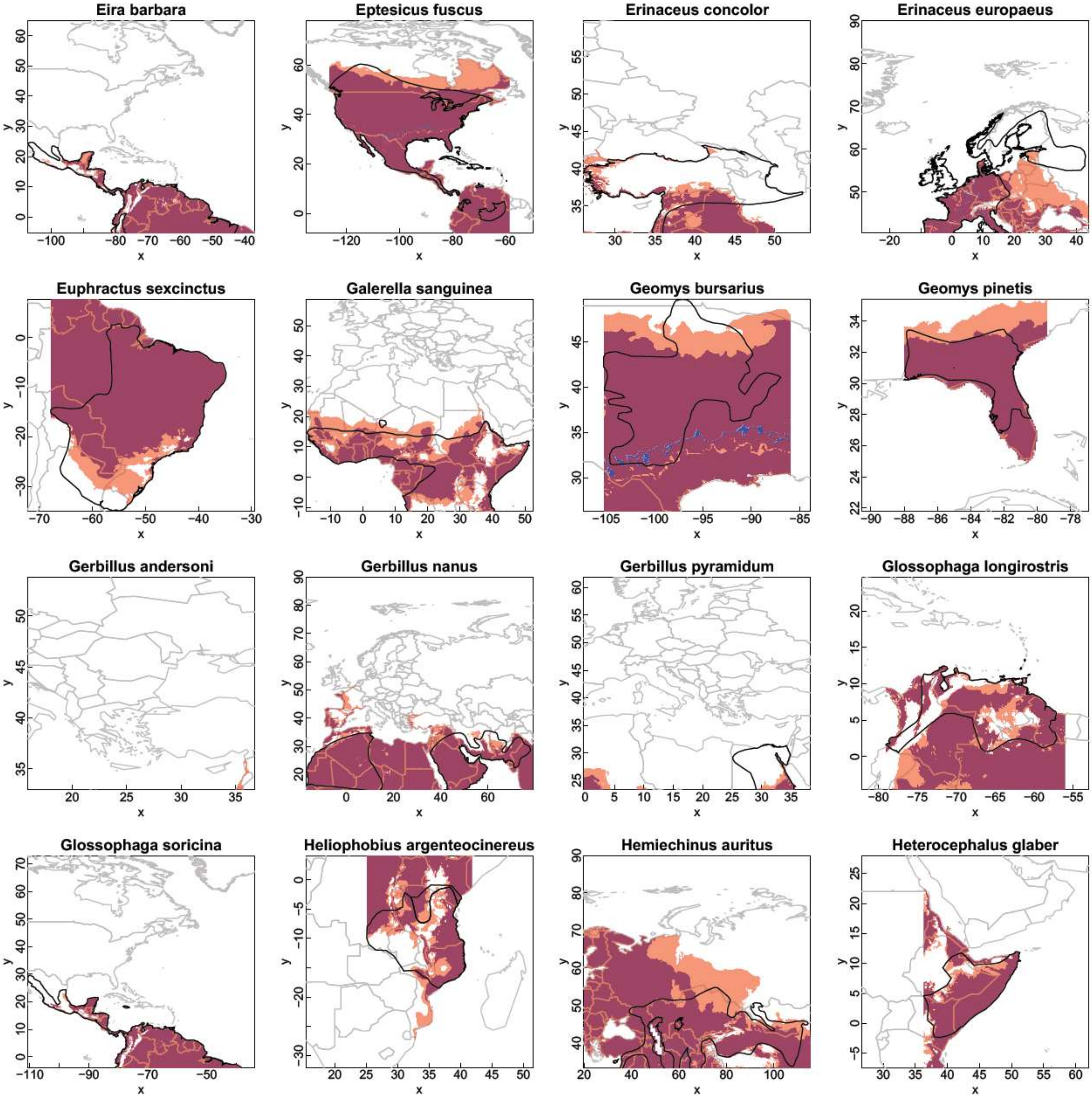
Eira barbara
Eptesicus fuscus
Erinaceus concolor
Erinaceus europaeus
Euphractus sexcinctus
Galerella sanguinea
Geomys bursarius
Geomys pinetis
Gerbillus andersoni
Gerbillus nanus
Gerbillus pyramidum
Glossophaga longirostris
Glossophaga soricina
Heliophobius argenteocinereus
Hemiechinus auritus
Heterocephalus glaber
x
y

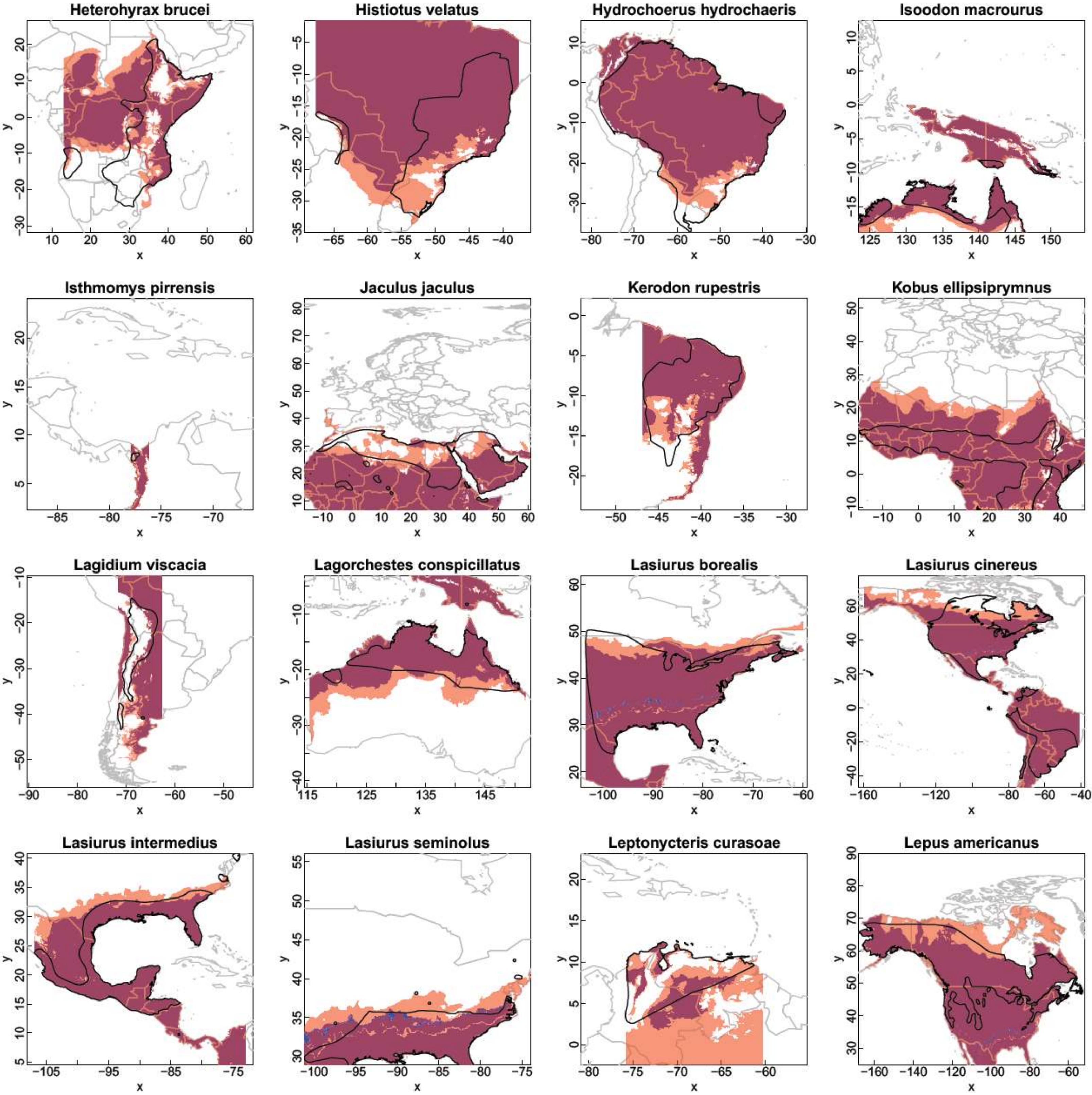
Heterohyrax brucei
Histiotus velatus
Hydrochoerus hydrochaeris
Isoodon macrourus
Isthmomys pirrensis
Jaculus jaculus
Kerodon rupestris
Kobus ellipsiprymnus
Lagidium viscacia
Lagorchestes conspicillatus
Lasiurus borealis
Lasiurus cinereus
Lasiurus intermedius
Lasiurus seminolus
Leptonycteris curasoae
Lepus americanus
x
y

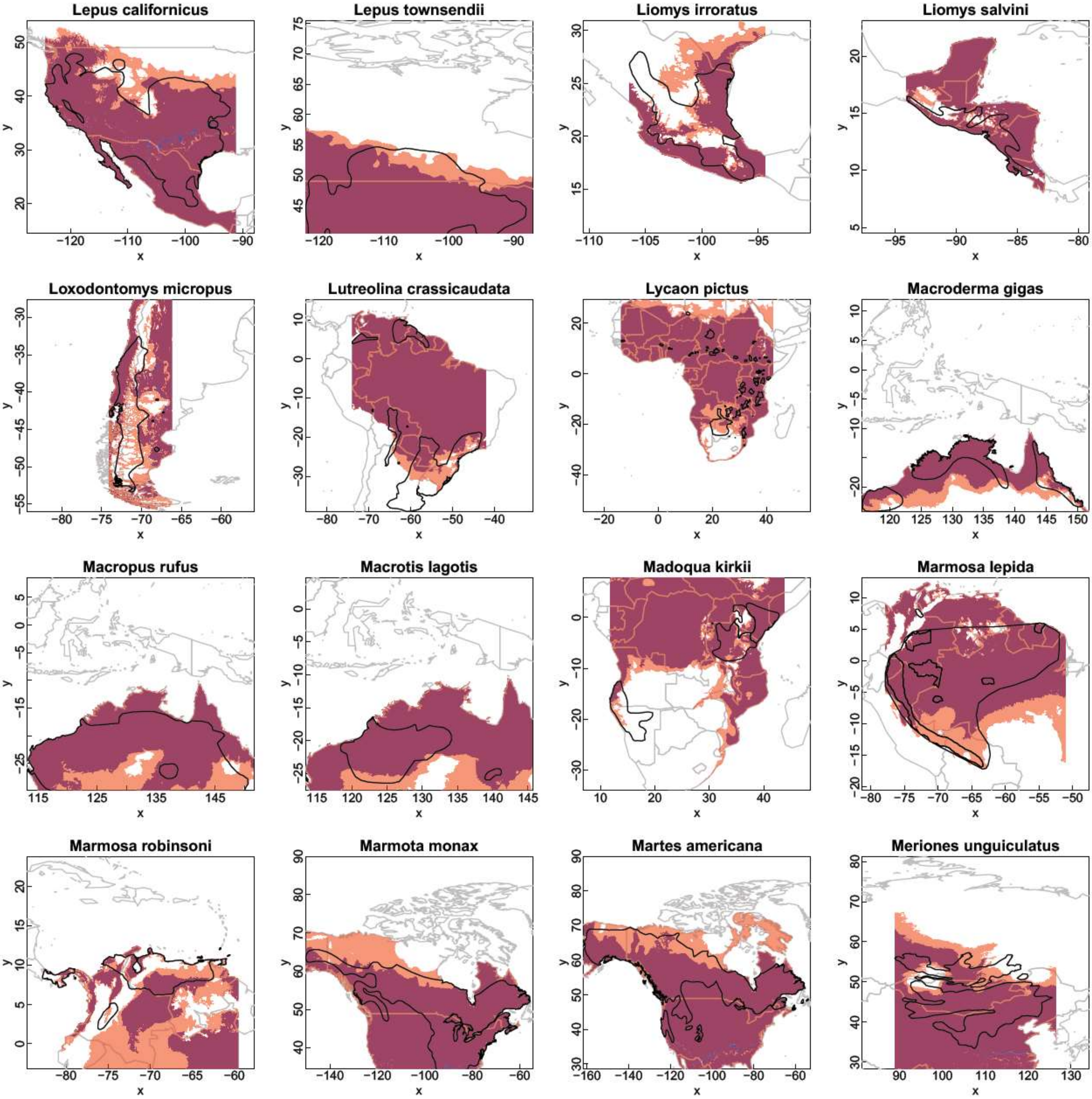
Lepus californicus
Lepus townsendii
Liomys irroratus
Liomys salvini
Loxodontomys micropus
Lutreolina crassicaudata
Lycaon pictus
Macroderma gigas
Macropus rufus
Macrotis lagotis
Madoqua kirkii
Marmosa lepida
Marmosa robinsoni
Marmota monax
Martes americana
Meriones unguiculatus
x
y

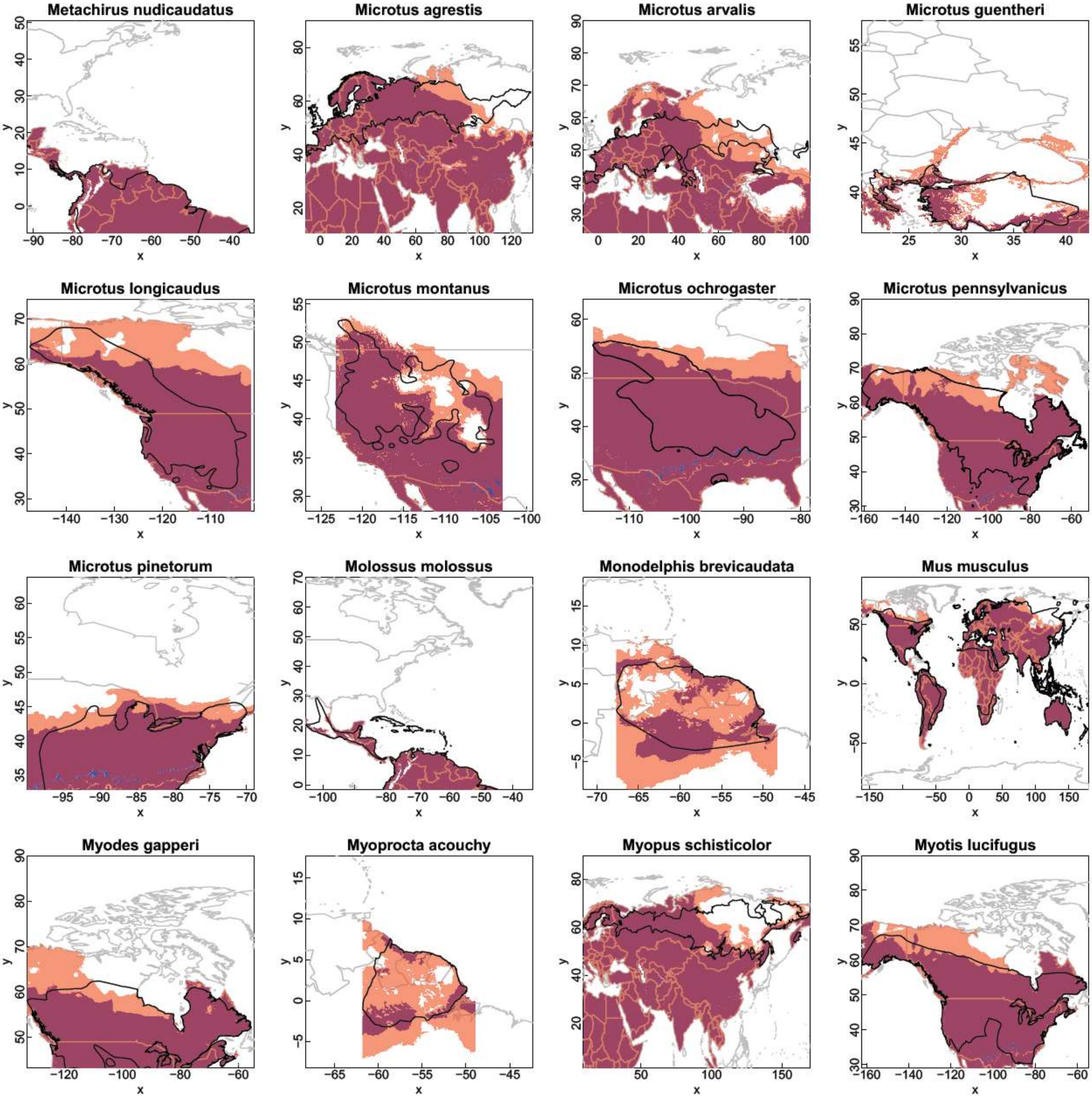
Metachirus nudicaudatus
Microtus agrestis
Microtus arvalis
Microtus guentheri
Microtus longicaudus
Microtus montanus
Microtus ochrogaster
Microtus pennsylvanicus
Microtus pinetorum
Molossus molossus
Monodelphis brevicaudata
Mus musculus
Myodes gapperi
Myoprocta acouchy
Myopus schisticolor
Myotis lucifugus

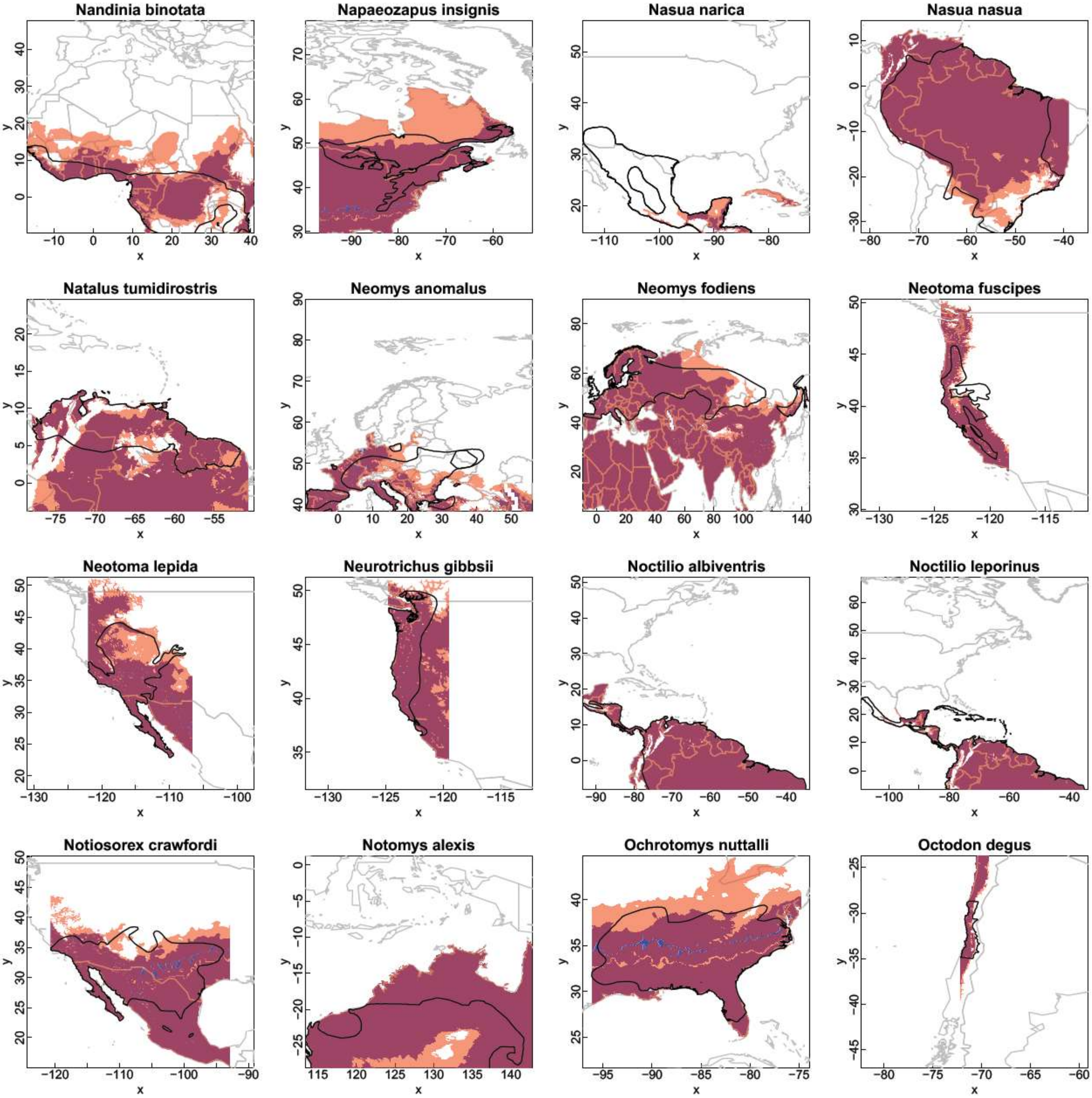
Nandinia binotata
Napaeozapus insignis
Nasua narica
Nasua nasua
Natalus tumidirostris
Neomys anomalus
Neomys fodiens
Neotoma fuscipes
Neotoma lepida
Neurotrichus gibbsii
Noctilio albiventris
Noctilio leporinus
Notiosorex crawfordi
Notomys alexis
Ochrotomys nuttalli
Octodon degus
x
y

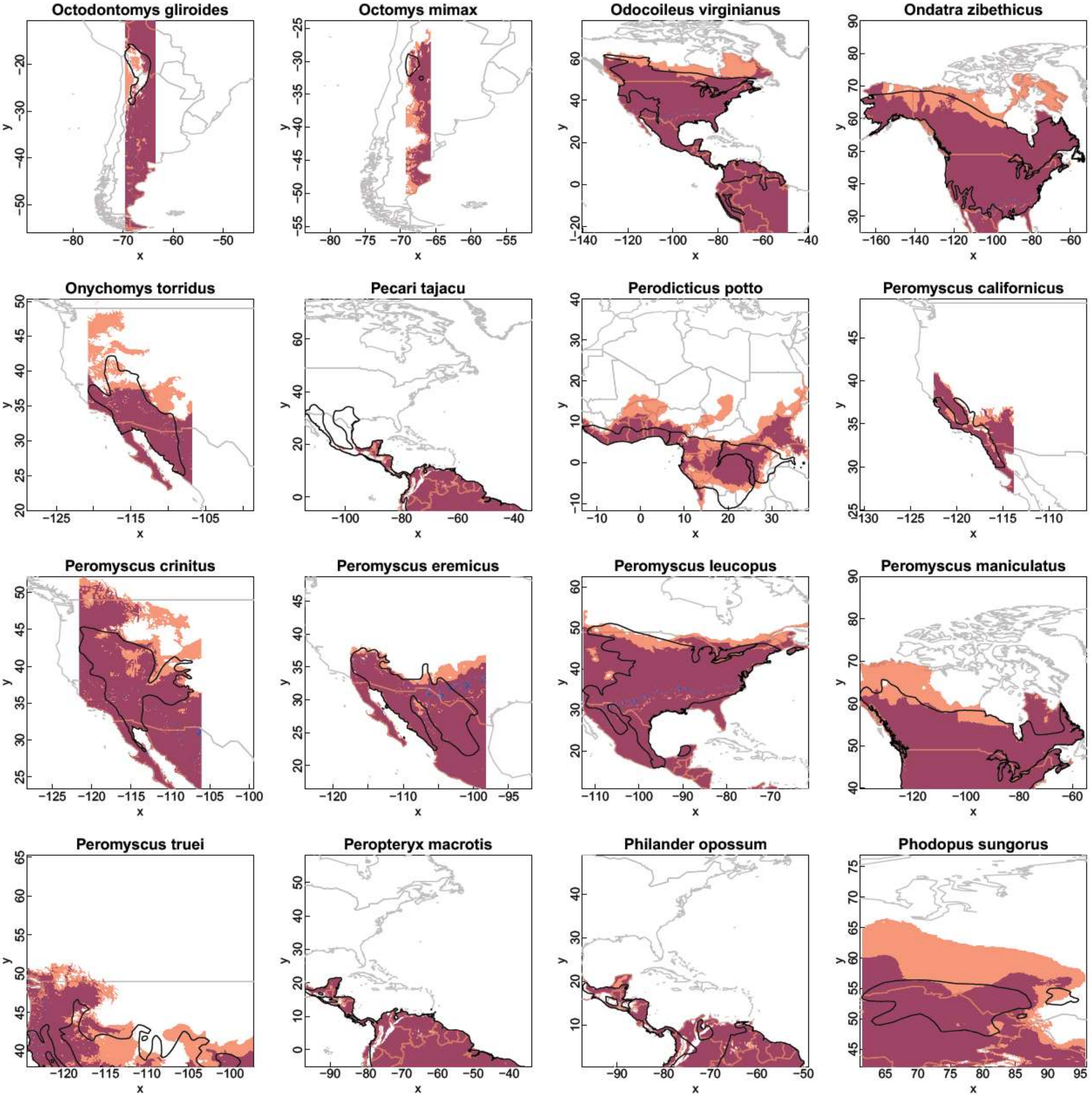
Octodontomys gliroides
Octomys mimax
Odocoileus virginianus
Ondatra zibethicus
Onychomys torridus
Pecari tajacu
Perodicticus potto
Peromyscus californicus
Peromyscus crinitus
Peromyscus eremicus
Peromyscus leucopus
Peromyscus maniculatus
Peromyscus truei
Peropteryx macrotis
Philander opossum
Phodopus sungorus
x
y

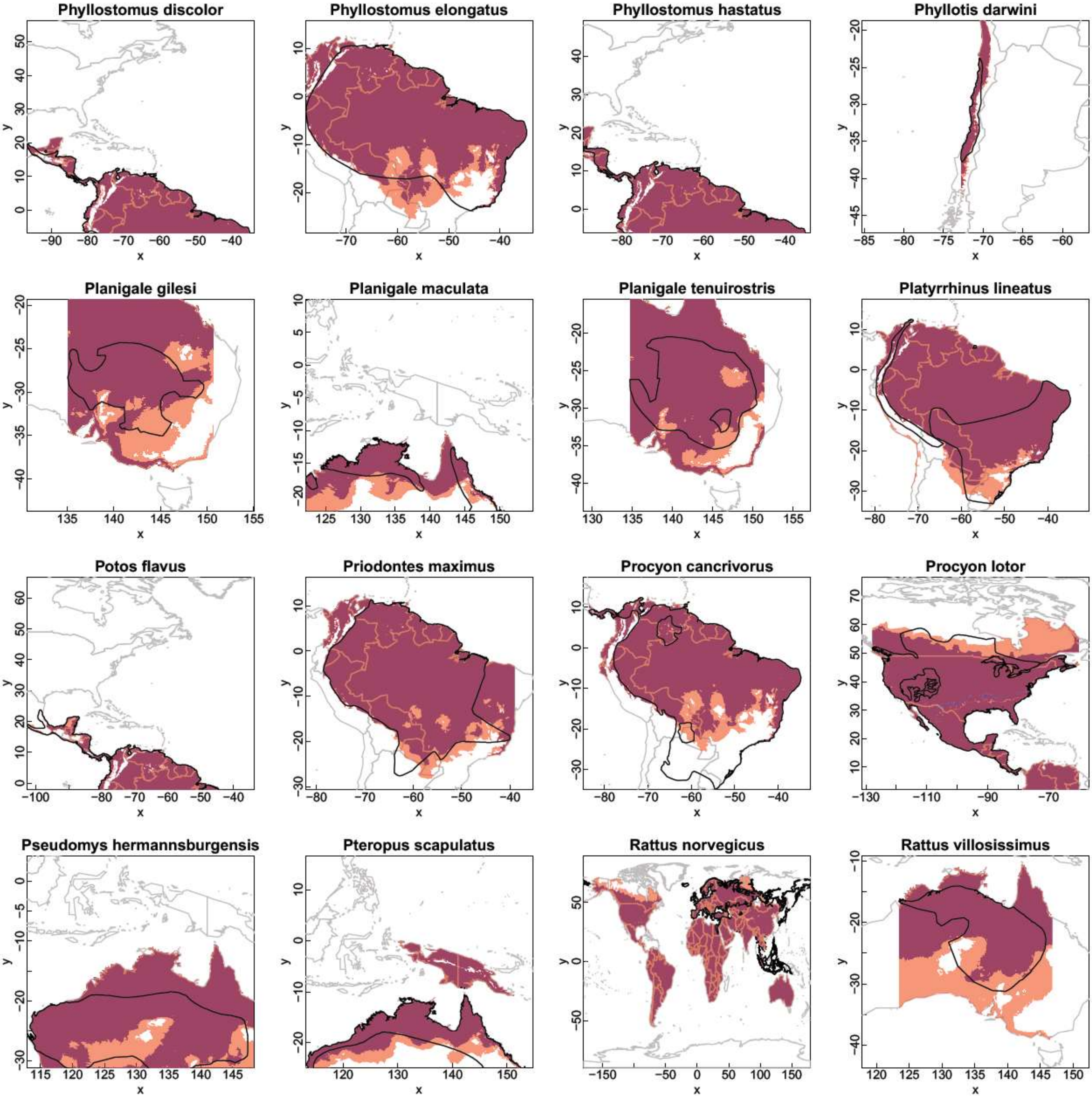
Phyllostomus discolor
Phyllostomus elongatus
Phyllostomus hastatus
Phyllotis darwini
Planigale gilesi
Planigale maculata
Planigale tenuirostris
Platyrrhinus lineatus
Potos flavus
Priodontes maximus
Procyon cancrivorus
Procyon lotor
Pseudomys hermannsburgensis
Pteropus scapulatus
Rattus norvegicus
Rattus villosissimus

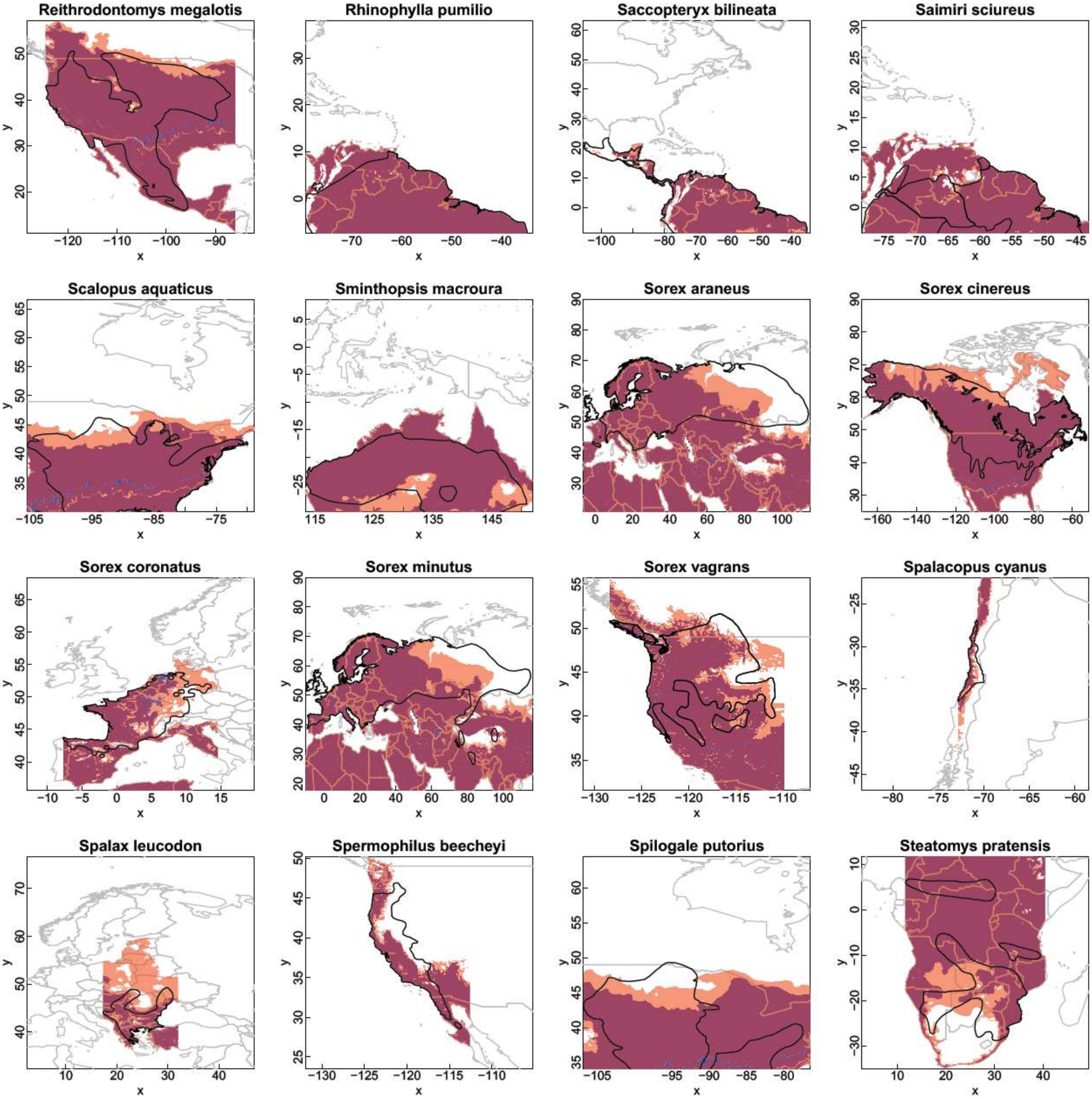
Reithrodontomys megalotis
Rhinophylla pumilio
Saccopteryx bilineata
Saimiri sciureus
Scalopus aquaticus
Sminthopsis macroura
Sorex araneus
Sorex cinereus
Sorex coronatus
Sorex minutus
Sorex vagrans
Spalacopus cyanus
Spalax leucodon
Spermophilus beecheyi
Spilogale putorius
Steatomys pratensis
x
y

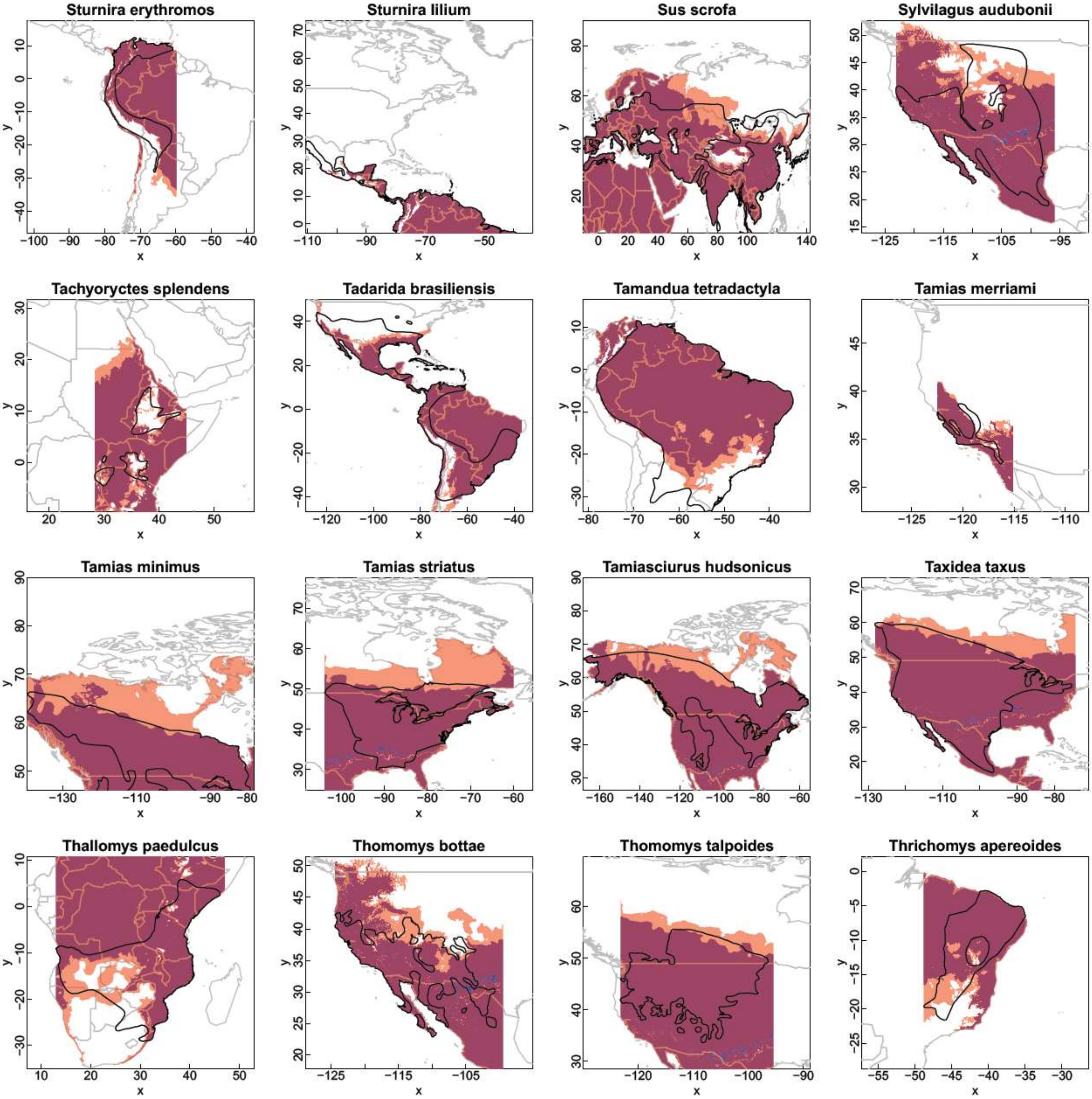
Sturnira erythromos
Sturnira lilium
Sus scrofa
Sylvilagus audubonii
Tachyoryctes splendens
Tadarida brasiliensis
Tamandua tetradactyla
Tamias merriami
Tamias minimus
Tamias striatus
Tamiasciurus hudsonicus
Taxidea taxus
Thallomys paedulcus
Thomomys bottae
Thomomys talpoides
Thrichomys apereoides
x
y

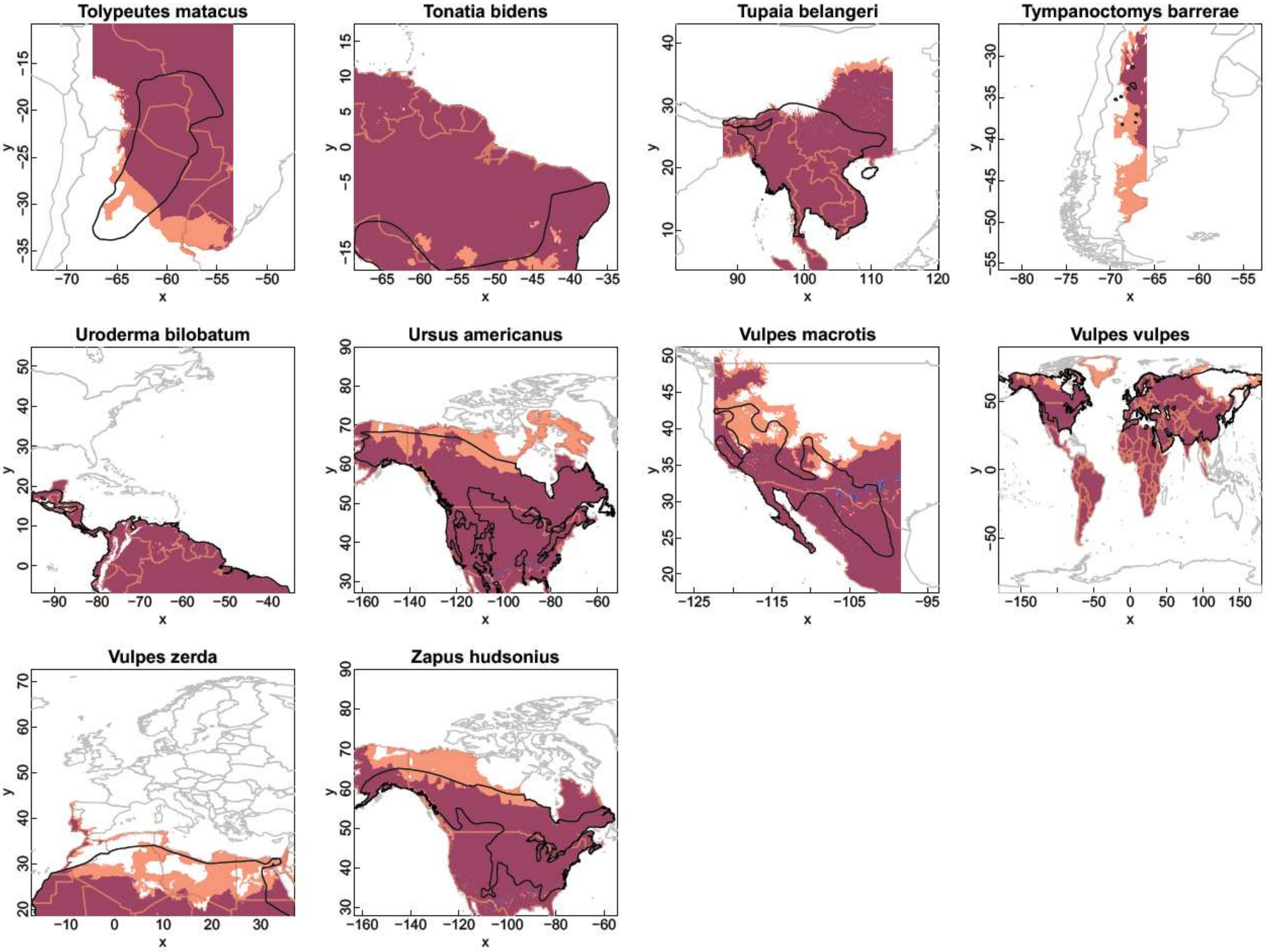

Tolypeutes matacus
−15 −20 −25 −30 −35
−70 −65 −60 −55 −50
Tonatia bidens
15 10 5 0 −5 −15
−65 −60 −55 −50 −45 −40 −35
Tupaia belangeri
40 30 20 10
90 100 110 120
Tympanoctomys barrerae
−30 −35 −40 −45 −50 −55
−80 −75 −70 −65 −60 −55
Uroderma bilobatum
50 40 30 20 10 0
−90 −80 −70 −60 −50 −40
Ursus americanus
90 80 70 60 50 40 30
−160 −140 −120 −100 −80 −60
Vulpes macrotis
50 45 40 35 30 25 20
−125 −115 −105 −95
Vulpes vulpes
50 0 −50
−150 −50 0 50 100 150
Vulpes zerda
70 60 50 40 30 20
−10 0 10 20 30
Zapus hudsonius
90 80 70 60 50 40 30
−160 −140 −120 −100 −80 −60
x
y

Figure S7. We depict observed bird cold range boundaries (CRB, black polygons: IUCN range maps) and those projected based on metabolic constraints in current (blue: 1950-2000) and predicted future (red: 2061-2080 from HadGEM2-AO model) climates (a – c). Purple shading indicates portions of the projected range occupancy that persists through climate warming. We note few areas of range contraction (blue) since we are only predicting CRBs (the depicted equatorward extent is not meaningful).

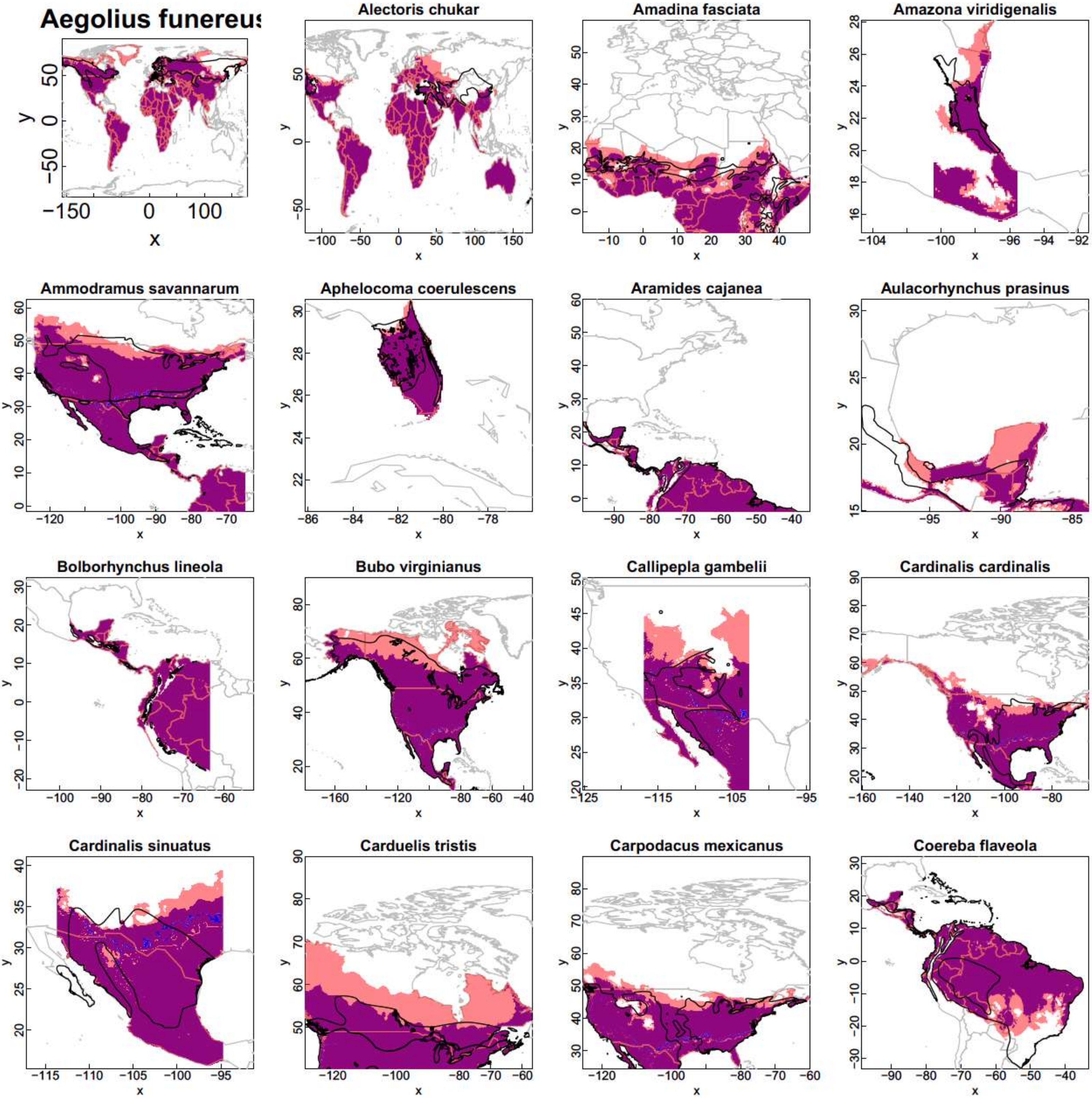
Aegolius funereus
Alectoris chukar
Amadina fasciata
Amazona viridigenalis
Ammodramus savannarum
Aphelocoma coerulescens
Aramides cajanea
Aulacorhynchus prasinus
Bolborhynchus lineola
Bubo virginianus
Callipepla gambelii
Cardinalis cardinalis
Cardinalis sinuatus
Carduelis tristis
Carpodacus mexicanus
Coereba flaveola
x
y

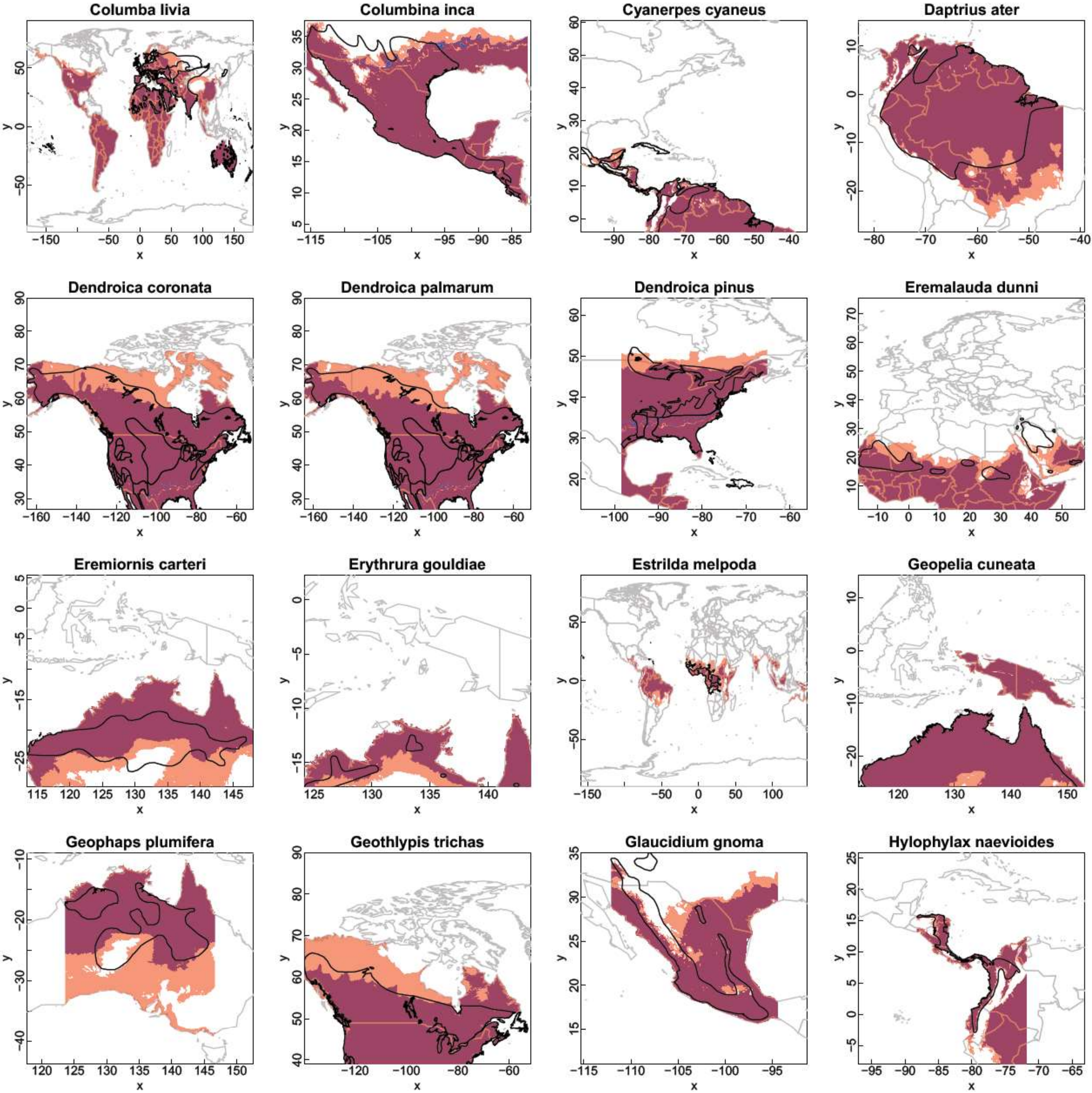
Columba livia
Columbina inca
Cyanerpes cyaneus
Daptrius ater
Dendroica coronata
Dendroica palmarum
Dendroica pinus
Eremalauda dunni
Eremiornis carteri
Erythrura gouldiae
Estrilda melpoda
Geopelia cuneata
Geophaps plumifera
Geothlypis trichas
Glaucidium gnoma
Hylophylax naevioides

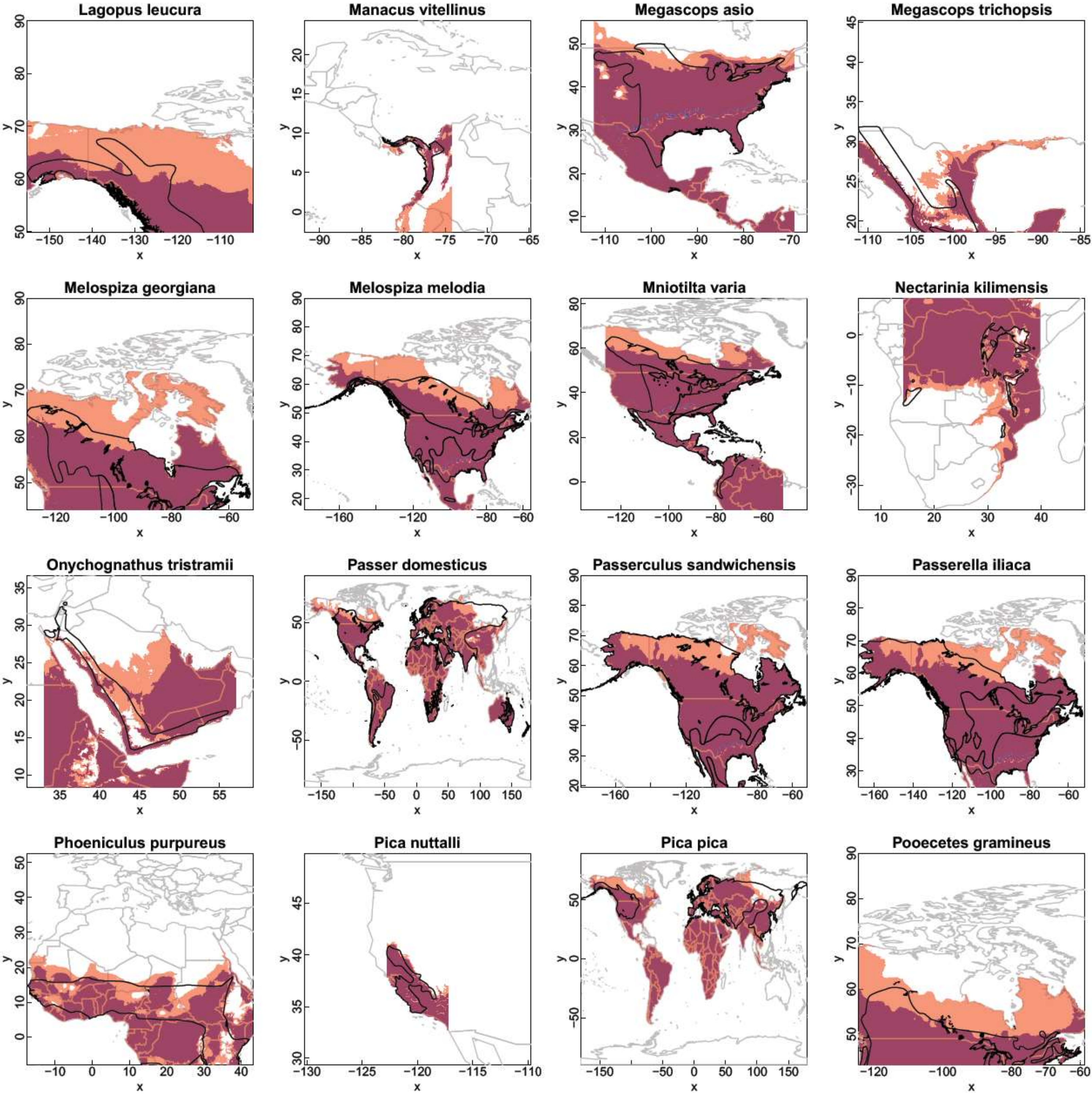
Lagopus leucura
Manacus vitellinus
Megascops asio
Megascops trichopsis
Melospiza georgiana
Melospiza melodia
Mniotilta varia
Nectarinia kilimensis
Onychognathus tristramii
Passer domesticus
Passerculus sandwichensis
Passerella iliaca
Phoeniculus purpureus
Pica nuttalli
Pica pica
Pooecetes gramineus
x
y

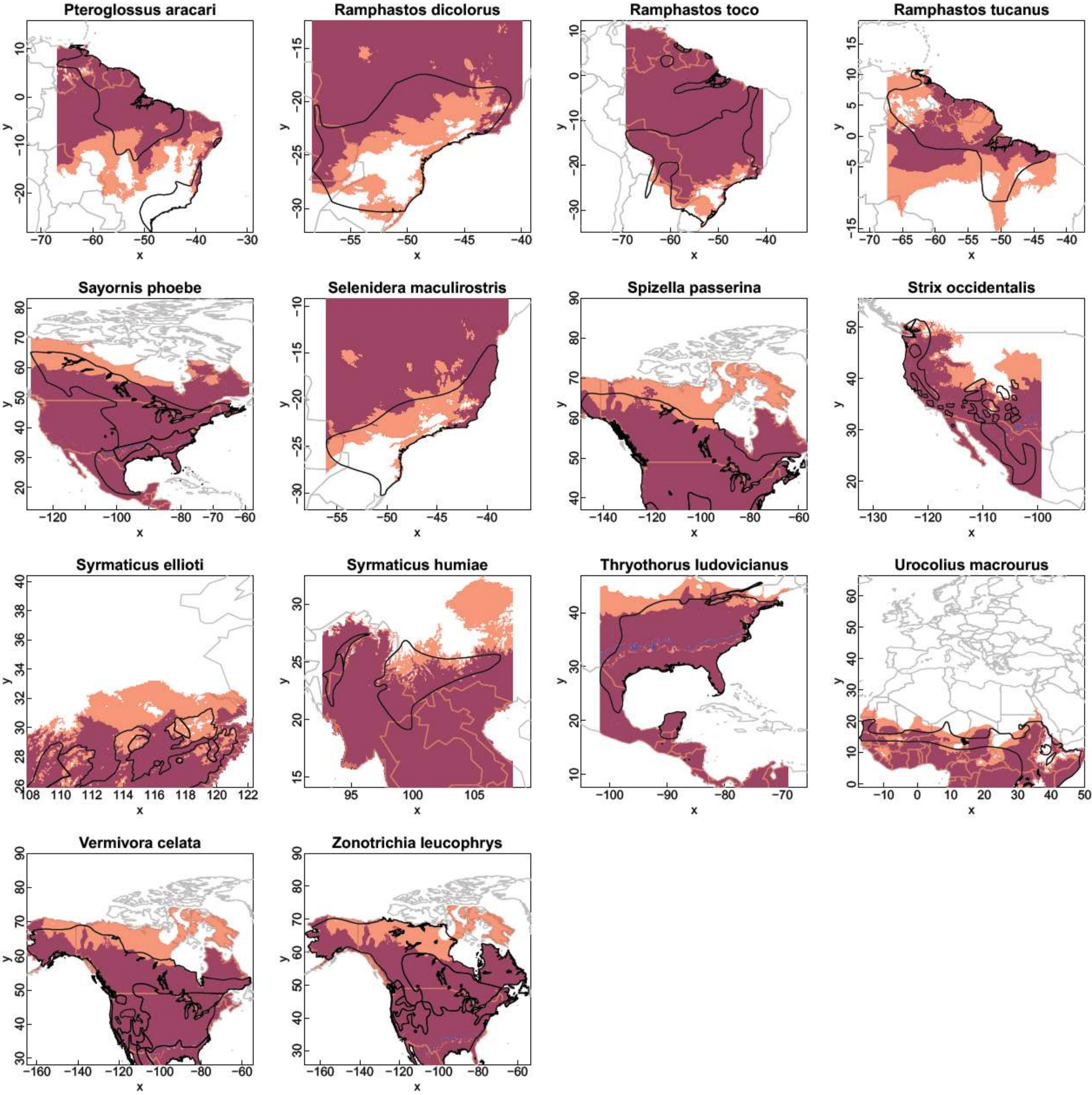
Pteroglossus aracari
Ramphastos dicolorus
Ramphastos toco
Ramphastos tucanus
Sayornis phoebe
Selenidera maculirostris
Spizella passerina
Strix occidentalis
Syrmaticus ellioti
Syrmaticus humiae
Thryothorus ludovicianus
Urocolius macrourus
Vermivora celata
Zonotrichia leucophrys

Figure S8. We depict observed bird cold range boundaries (CRB, black polygons: IUCN range maps) and those projected based on metabolic constraints in current (blue: 1950-2000) and predicted future (red: 2061-2080 from HadGEM2-AO model) climates (a – c). Purple shading indicates portions of the projected range occupancy that persists through climate warming. We note few areas of range contraction (blue) since we are only predicting CRBs (the depicted equatorward extent is not meaningful).

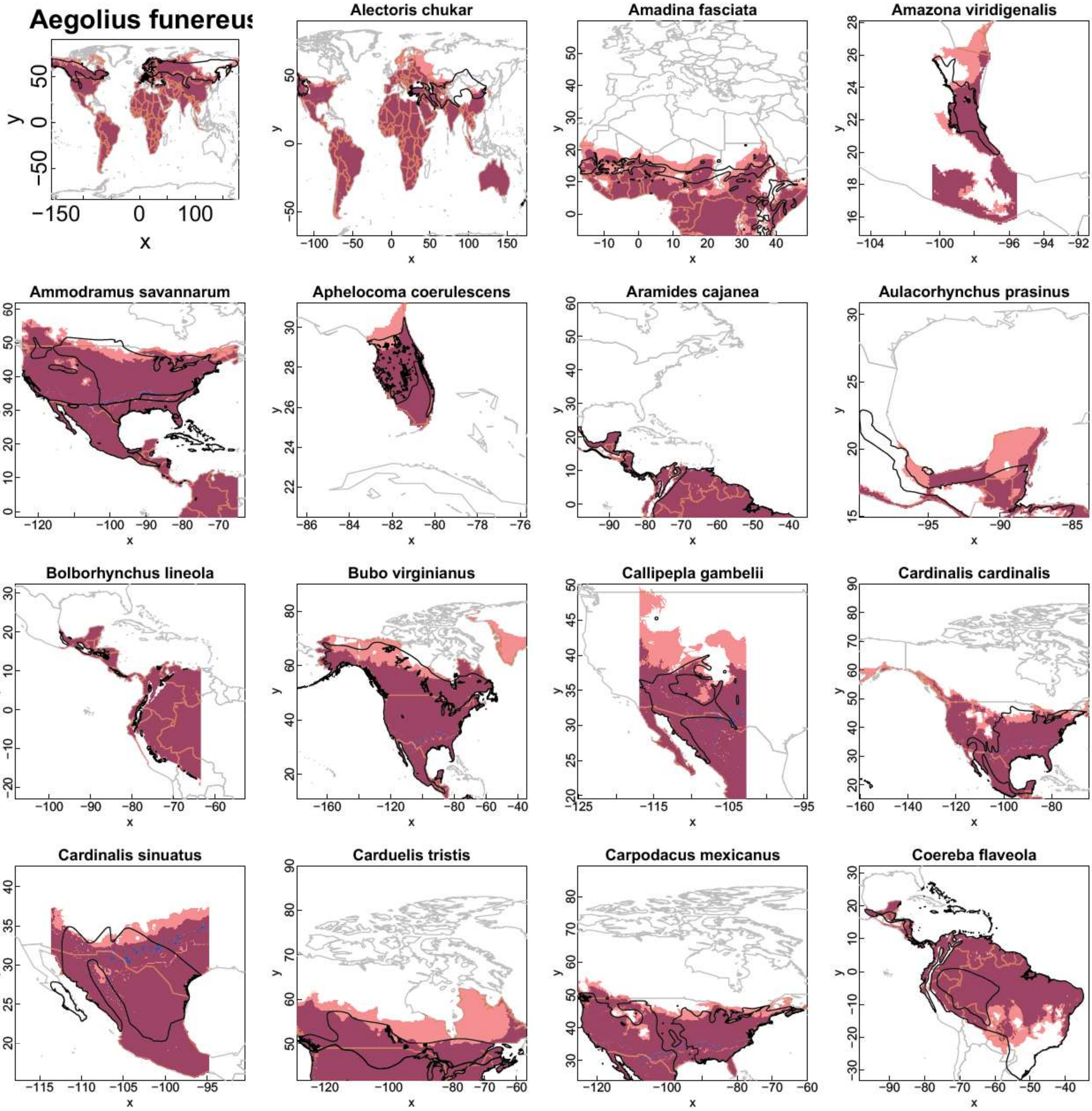
Aegolius funereus
Alectoris chukar
Amadina fasciata
Amazona viridigenalis
Ammodramus savannarum
Aphelocoma coerulescens
Aramides cajanea
Aulacorhynchus prasinus
Bolborhynchus lineola
Bubo virginianus
Callipepla gambelii
Cardinalis cardinalis
Cardinalis sinuatus
Carduelis tristis
Carpodacus mexicanus
Coereba flaveola
x
y

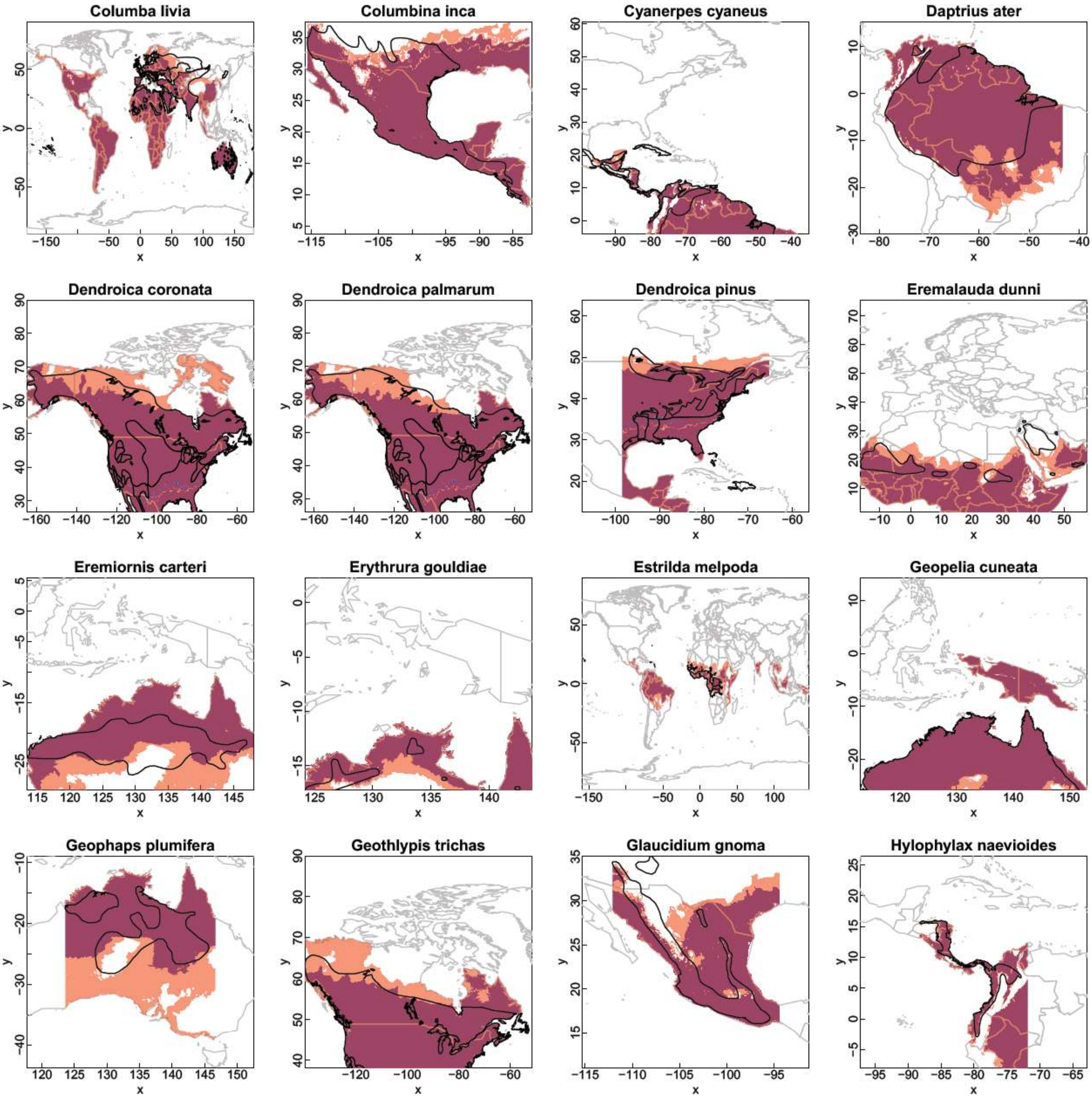
Columba livia
Columbina inca
Cyanerpes cyaneus
Daptrius ater
Dendroica coronata
Dendroica palmarum
Dendroica pinus
Eremalauda dunni
Eremiornis carteri
Erythrura gouldiae
Estrilda melpoda
Geopelia cuneata
Geophaps plumifera
Geothlypis trichas
Glaucidium gnoma
Hylophylax naevioides

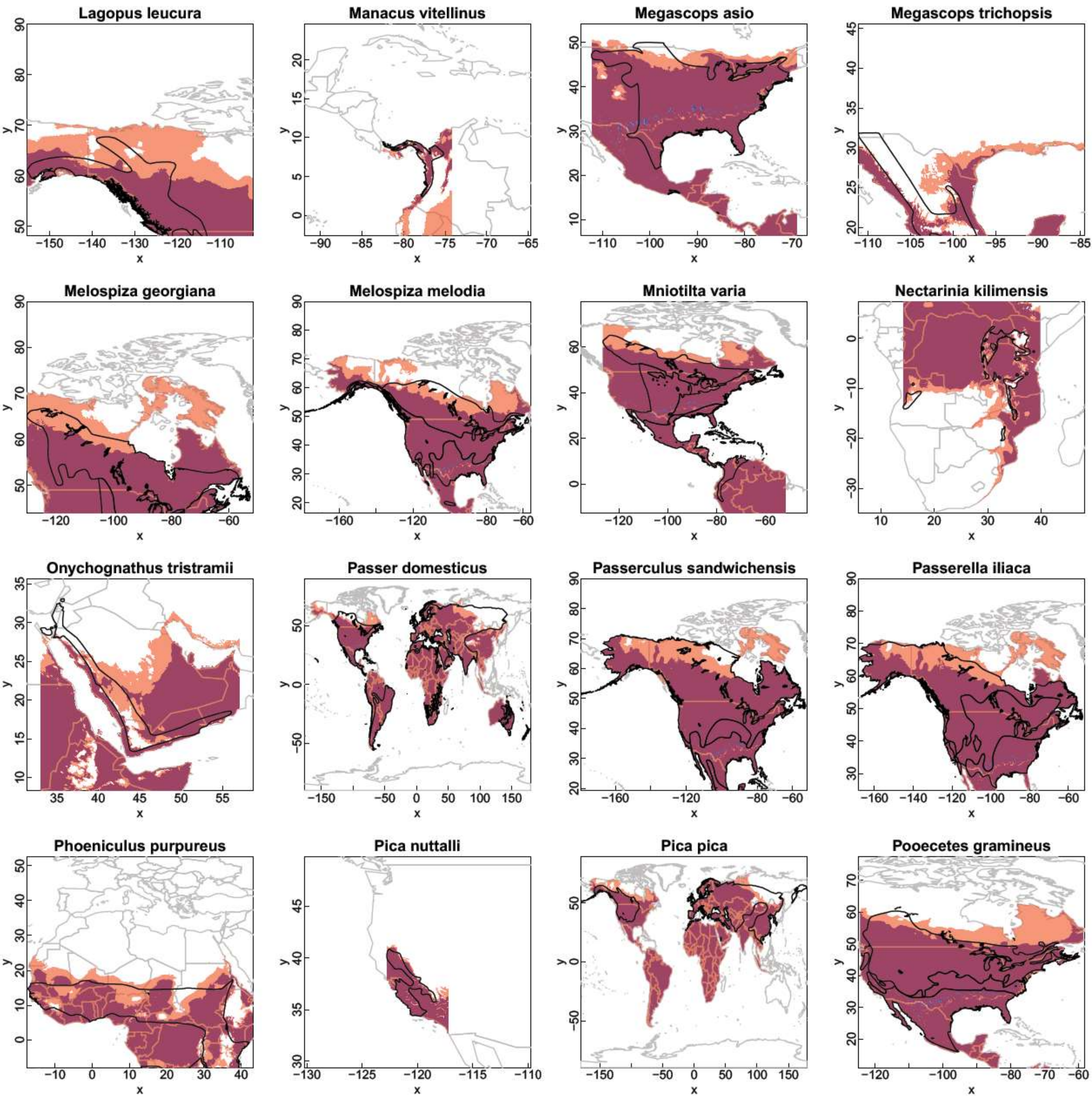
Lagopus leucura
Manacus vitellinus
Megascops asio
Megascops trichopsis
Melospiza georgiana
Melospiza melodia
Mniotilta varia
Nectarinia kilimensis
Onychognathus tristramii
Passer domesticus
Passerculus sandwichensis
Passerella iliaca
Phoeniculus purpureus
Pica nuttalli
Pica pica
Pooecetes gramineus
x
y

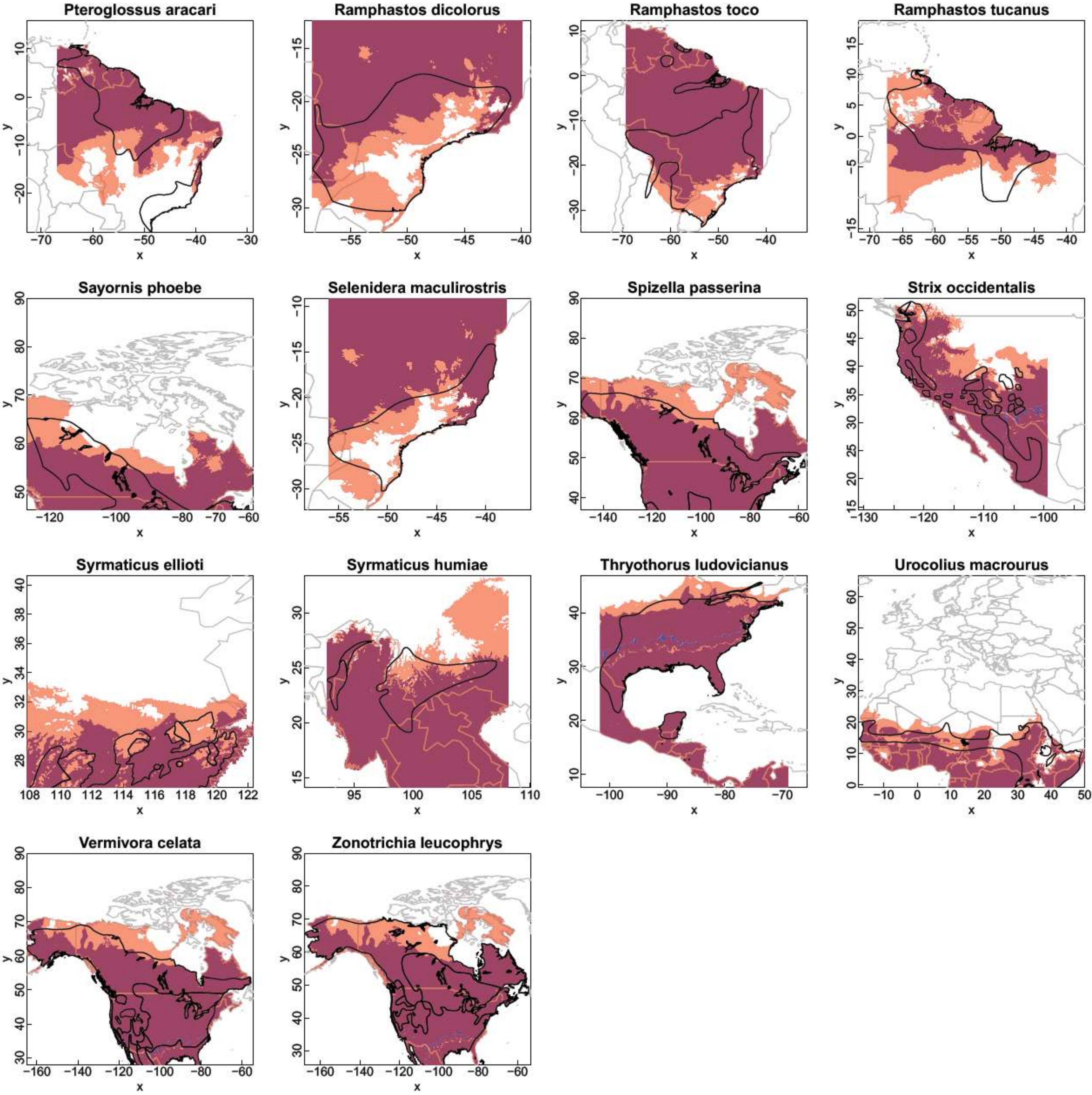
Pteroglossus aracari
Ramphastos dicolorus
Ramphastos toco
Ramphastos tucanus
Sayornis phoebe
Selenidera maculirostris
Spizella passerina
Strix occidentalis
Syrmaticus ellioti
Syrmaticus humiae
Thryothorus ludovicianus
Urocolius macrourus
Vermivora celata
Zonotrichia leucophrys
